# The Elucidation of the Interactome of 16 *Arabidopsis* bZIP Factors Reveals Three Independent Functional Networks

**DOI:** 10.1371/journal.pone.0139884

**Published:** 2015-10-09

**Authors:** Carles Marco Llorca, Kenneth Wayne Berendzen, Waqas Ahmed Malik, Stefan Mahn, Hans-Peter Piepho, Ulrike Zentgraf

**Affiliations:** 1 Center for Plant Molecular Biology (ZMBP), University of Tübingen, Tübingen, Germany; 2 Biostatistics Unit, Institute of Crop Science, University of Hohenheim, Stuttgart, Germany; USDA Agricultural Research Service, UNITED STATES

## Abstract

The function of the bZIP transcription factors is strictly dependent on their ability to dimerize. Heterodimerization has proven to be highly specific and is postulated to operate as a combinatorial mechanism allowing the generation of a large variety of dimers with unique qualities by specifically combining a small set of monomers; an assumption that has not yet been tested systematically. Here, the interaction pattern and the transactivation properties of 16 *Arabidopsis thaliana* bZIPs are examined in transiently transformed *Arabidopsis* protoplasts to deliver a perspective on the relationship between bZIP dimerization and function. An interaction matrix of bZIPs belonging to the C, G, H, and S1 bZIP groups was resolved by Bimolecular Fluorescent Complementation (BiFC) coupled to quantitative flow cytometric analysis, while an extensive GUS reporter gene assay was carried out to determine the effect of different bZIP pairs on the expression of four different known bZIP-targeted promoters. Statistical data treatment and complementary bioinformatic analysis were performed to substantiate the biological findings. According to these results, the 16 bZIPs interact in three isolated networks, within which their members dimerize non-specifically and exhibit a significant level of functional redundancy. A coherent explanation for these results is supported by *in silico* analysis of differences in the length, structure and composition of their leucine zippers and appears to explain their dimerization specificity and dynamics observed *in vivo* quite well. A model in which the bZIP networks act as functional units is proposed.

## Introduction

The regulation of the gene expression is essential for plant growth and differentiation, as it adjusts the proteome to varying needs in response to environmental and developmental cues. Due to the sessile nature of plants, development is especially shaped by the environment as an adaptive response, in contrast to buffered development in animals. Transcriptional control is one of the most important means for regulating gene expression, and in plants is indeed especially complex, encapsulated by the significant expansion of their transcription factor families during evolution [1, 2]. This allows complex network circuitries in which multiple inputs act in parallel in order to provide enhanced adaptive mechanisms [3].

One of the largest groups of transcription factors in plants is the basic region/leucine zipper (bZIP) family whose members regulate critical processes in development and stress responses [4–10]. All members of this family feature a bZIP domain which consists of a basic region (BR) followed by a leucine zipper (LZ), a subtype of coiled coil motif. The BR carries a nuclear localization signal (NLS) and directly interacts with DNA, whereas the LZ mediates bZIP dimerization. Based on the conserved sequence of the BR and other functional motifs outside of the bZIP domain, the *Arabidopsis* bZIPs are sorted in 10 groups (named **A** to **I**, plus **S**), so that bZIPs within the same group are predicted to bind to similar cis-elements and share functional similarities [11].

The functional form of bZIPs is a dimer; hence partner matching plays an essential role in bZIP function. For this reason, bZIP dimerization has been extensively investigated to decipher the forces governing this process [12–18]. Dimerization between bZIPs takes place through their LZs, with the two alpha helices running in parallel and wrapping around each other to form a supercoil. All coiled coil motifs are characterized by the repetition of a seven amino acid sequence, termed heptad, within which the composing amino acid residues are designated by letters from **a** to **g**. The positions **a** and **d** are situated on the same side of the alpha helix and are occupied by hydrophobic residues, while the positions **b**, **c**, and **f** lie on the opposite side of the helix carrying polar amino acids, thus forming an amphipathic helix. The hallmark of the LZ is the presence of leucine residues in the **d** positions of these heptads. The main force driving the dimerization between two LZ motifs is the hydrophobic effect, so that amino acids in the **a** and **d** positions interact with their counterparts in the opposite monomer creating the hydrophobic core that leads to the formation of the supercoil structure. In addition, electrostatic interactions between charged residues in the **e** position of one helix and the **g** position of the other mediate specific pairing dependent on whether or not the **e**-**g** pairs result in attractive or repulsive charges.

Based on these rules, *Arabidopsis* bZIPs were predicted *in silico* to be arranged in many small, independent interacting groups, in contrast to other species which resulted in few interacting groups extensively interconnected among each other [19, 20]. The majority of the experimentally confirmed bZIP dimers are in agreement with these predictions, but bZIP combinations not predicted to interact have rarely been tested. In one study, several unpredicted combinations were identified forming a network [21]. Therefore, the architecture of the *Arabidopsis* bZIP network remains unsolved and it has not yet been demonstrated whether it actually consists of small, isolated bZIP networks, or a meta-network in which these small bZIP networks are linked via inter-network connections.

The notion, that each bZIP monomer defines particular DNA-binding and transactivation properties, led to the suggestion that the dimerization process operates as a combinatorial mechanism generating a large variety of dimers with unique properties. However, even though this is presumed to be a central feature of the bZIP function, studies confirming this assumption are rare. The transcriptional effect of different bZIP combinations has not been analyzed systematically and bZIP research often focuses on only a few dimers, lacking the wider perspective needed to decipher the complexity of bZIP networks. In this paper, we address these questions by dissecting the interaction and function of 16 bZIPs, representing one fifth of the *Arabidopsis* bZIP family and spanning four different bZIP groups.

In *Arabidopsis*, the largest interacting group of bZIPs so far described is the so called C/S1 network, which includes four bZIPs from the C class and five from the S1 class [21, 22]. These bZIPs have been characterized in responses to abiotic stress conditions such as high salt or darkness, carbohydrate homeostasis, and seed development [10, 23–25]. In this work, this network was challenged with bZIPs belonging to the G and H classes, as they are involved in physiologically related processes, such as light signaling and seed development [26–29]. An interaction map among these 16 bZIPs was generated by Bimolecular Fluorescent Complementation (BiFC) in transiently transformed *Arabidopsis* protoplasts and resulted in three separate networks in which their members dimerize promiscuously. This was followed up with GUS reporter gene transactivation assays performed with a selection of 47 bZIP dimer combinations in order to assess their effect on four bZIP-regulated genes: *PROLINE DEHYDROGENASE* (*PDH*), *ASPARAGINE SYNTHETASE 1* (*ASN1*), *CATALASE 2* (*CAT2*), and *RUBISCO SMALL SUBUNIT 1 (RBCS1a*). These assays revealed that members of the same network exhibit partially redundant transactivation properties, but distinct for each promoter. Based on these data, a model is proposed in which *Arabidopsis* bZIPs are organized into small functional networks.

## Material and Methods

### Protoplast transformation

Protoplasts were generated from 3-day-old Arabidopsis Col-0 dark-grown cell suspension culture, and the transformations were carried out in small scale in 96 well plates with round-bottom (Roth) as previously described [30] using a Liquidator^96^ Manual Pipetting system (Steinbrenner GmbH, Germany). The DNA was purified using either the Gene JET Plasmid Midiprep kit or the Maxiprep version (Thermo Scientific), eluting in water and adjusting the final concentration to 1μg/μl.

### BiFC Assay

For the BiFC assay, the coding sequences of the 16 bZIPs were cloned into the pSPYNE-35S/pUC-SPYNE and the pSPYCE-35S/pUC-SPYCE plasmids, the former carrying the N-terminal half of the YFP and the latter the C-terminal part [31]. Protoplasts were transformed with pairwise constructs containing 2 μg of each plasmid (i.e. pUC-SPYNE and pUC-SPYCE), covering the 256 possible combinations between the 16 pUC-SPYNE constructs and the 16 pUC-SPYCE ones, and incubated overnight. The day after, the protoplasts were analyzed by flow cytometry with at least three biological replicates per sample and counting a minimum of 30.000 cells. The fluorescence level was counted as a binomial count of the fluorescent cells from total number of cells in a sample. To estimate differences in fluorescent counts between the 16 bZIPs, a generalized linear mixed model framework (GLMM) was used in SAS [32].

The protoplasts counts were treated as binomial random variables conditional on their means, which were modeled using the logit link in Proc GLIMMIX. The model included fixed effects for the fusion conformations on N-terminal of the YFP (SPC), C-terminal of the YFP (SPN), and their interaction effect; and a random effect for each transformation batch (96-well plate). Additionally, a normally distributed random effect was included for each experimental unit (well) to account for overdispersion. The GLMMs were fitted using the pseudo-likelihood method with the variance function *μ* ^1.7^(1−*μ*)^1.7^, as the usual variance function for binomial distributions (i.e., *μ*(1−*μ*)) did not satisfactorily describe the variability of the data.

The hypotheses tests of interest were conducted using the contrast statement within Proc GLIMMIX, and the resulting *p*-values for each hypothesis test were adjusted to maintain the overall 5% Type I error rate using Tukey’s adjustment method.

### Gus reporter assay

For the GUS reporter assay, the regulatory sequences comprising 1.5 kbp of the upstream region and the 5’UTR until the initial ATG (which was not included) of the four genes were first cloned into p-ENTR/D-TOPO vector, and subsequently sub-cloned into the GUS reporter plasmid pBGWFS7 [33]. The assay was performed by transforming the protoplasts with 2 μg of the pBGWFS7 constructs and a total of 4 μg of effector plasmid (either 2 μg of two different bZIPs for the heterodimers, or 2 μg of one bZIP and 2μg of the empty effector plasmid for the homodimers). In addition, 0.1 μg of a 35S::Luciferase plasmid was co-transformed for normalization purposes. The effector proteins were expressed using a modified pUC-SPYNE plasmid in which the YFP fragment had been removed by site-directed deletion performed with the QuickChange II mutagenesis kit from Agilent (S1 Table).

The day after the transformation, the protoplasts were pelleted by adding 1.7 mL of MMM solution (0.5 M Mannitol, 15 mM MgCl2, 0.1% MES, pH 5.8) to each well and centrifuging 5 min at 400 g. Then, the supernatant was removed using the Liquidator^96^ until 50 μl were left in each well, and these were mixed thoroughly with 50 μl of a 2X Lysis Buffer (Cell Culture 5X Lysis Buffer from Promega and cOmplete Tablets Proteinase Inhibitor without EDTA from Roche). The mix was then incubated for 30 minutes on ice, and centrifuged other 30 minutes at 4000 g, 4°C. The GUS assay was then performed with 10 μl of the supernatant in 90 μl of the GUS Assay Buffer (0.1 mM Tris pH 8.0, 2 mM MgCl_2_, and 2 mM 4-Methylumbelliferyl-β-D-glucuronide (Roth)). The reaction was continuously monitored as previously described [34], with an excitation wavelength of 355 nm and measuring emission at 460 nm. Luciferase activity was quantified for 10 μl of the protein extract in 50 μl of Luciferase reagent (Promega) and the emitted light was measured for 0.1 seconds. All measurements were done in a 96 well plate reader (Tristar LB941 from Berthold).

The GUS activity values are calculated as the quotient of the slope in the GUS assay by the measured light in the Luciferase assay. We fitted linear mixed models on GUS/LUC ratios for each of the four promoters. The models included a fixed effect for bZIP combination and a random effect of batch of measurements (plate), and considering heterogeneous variance grouped by each bZIP. Hypothesis tests were conducted for the four promoters separately between each bZIP combination and the reference control, and the resulting *p*-values were adjusted to maintain an overall 5% Type I error rate using Dunnett’s adjustment method [35].

### Subcellular localization

For the subcellular localization of the bZIPs, their coding-regions were cloned into the pH7WGF2.0 plasmid carrying a C-terminal GFP fusion [33] and expressed in protoplasts together with a pCF205 (35S::mCherry:NLS, KWB) plasmid. Observation was performed in a Leica TCS SP8 Confocal Laser Scanning Microscope.

### qPCR

The total RNA was extracted with the RNAeasy Plant Mini Kit (Qiagen) from 6 week-old *Arabidopsis thaliana* Col-0 plants grown at 20°C under long day conditions (16h light/8h dark) with a light intensity of 100–120 μmol s^-1^ m^-2^. The cDNA synthesis was performed with 0.5 μg of RNA using the Revertaid Reverse Transcriptase (Fermentas). The qPCR were performed with iTAQ universal SYBR green supermix (Bio-Rad) in a final volume of 10 μl, including 2 μl of 1:6 diluted cDNA and the primers at 1 μM concentration.

### Software

Flow cytometry data was processed with FlowJo v10.0.7, and statistical analyses were performed with JMP11 or with SAS 9.4.

## Results

### Generation and testing of a dimerization matrix for 16 bZIP proteins

Because all bZIPs feature a LZ dimerization motif, they could all potentially interact with each other. Conversely, the singular amino acid composition of each LZ determines the structure of the dimerization interface and thereby should give specificity to the interaction. To give insight in the organization of bZIP interaction networks, it is of interest to determine not only which combinations of bZIPs physically interact, but also which ones are restricted. Therefore, we screened for interactions all possible combinations between 16 *Arabidopsis thaliana* bZIPs. Here, we will refer to a whole class of bZIPs by using the group designation letter as a prefix to the “bZIP” acronym. Accordingly, the subset of bZIPs studied is composed by the C-bZIPs (bZIP9, bZIP10, bZIP25, and bZIP63), the G-bZIPs (GBF1, GBF2, GBF3, bZIP16, and bZIP68), the H-bZIPs (Hy5 and HyH), and a subset of the S-bZIPs known as S1-bZIPs (bZIP1, bZIP2, bZIP11, bZIP44, and bZIP53) [11, 21].

**Fig 1:**
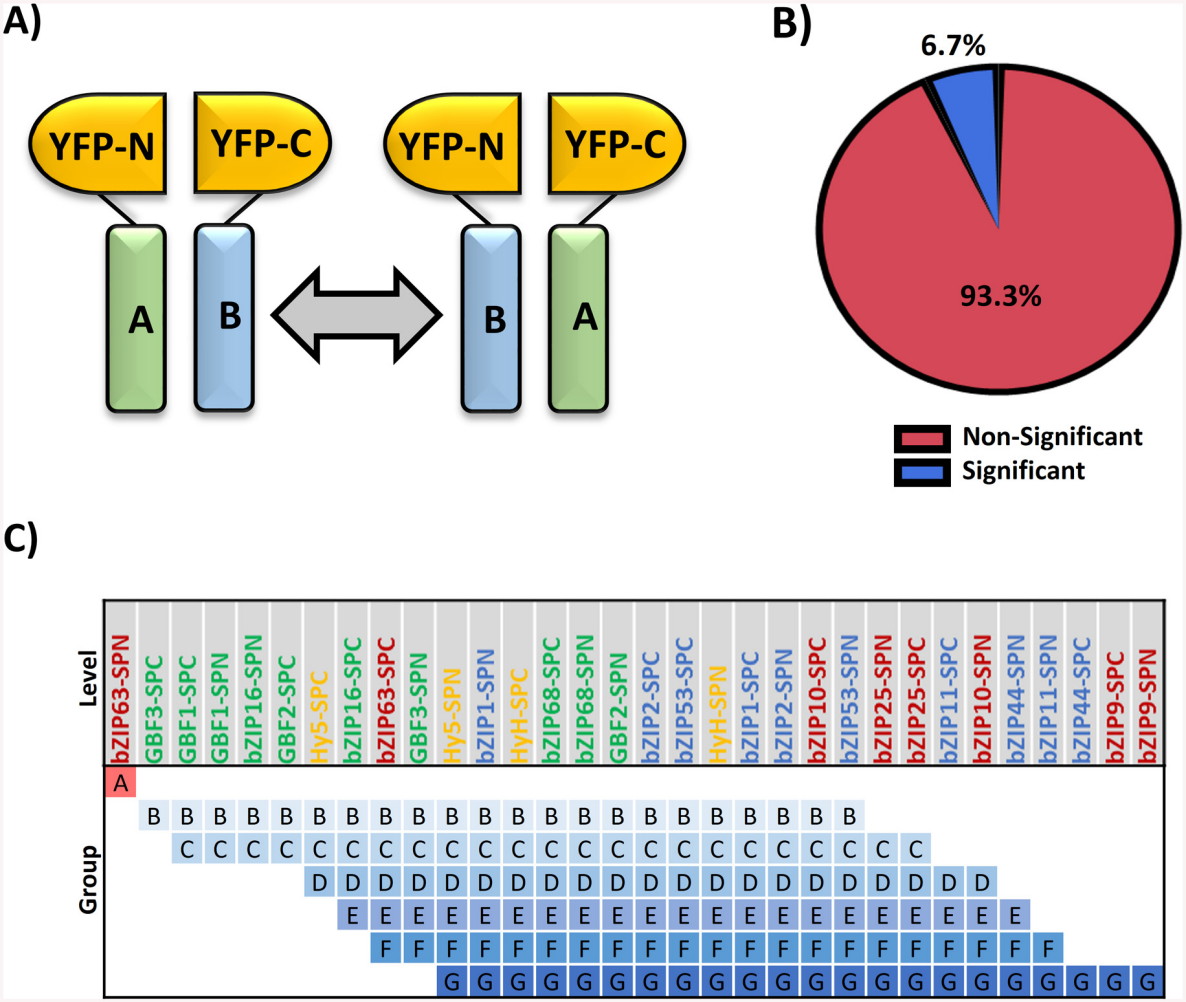
Assessment of the matrix reciprocity. **A)** Each pair of bZIPs was tested in both fusion conformations of the split YFP according to the cartoon. **B)** The statistical comparison of all pairs of equivalent combinations results in 6.7% significant differences. **C)** Connecting Letters Report comparing the means of all the measurements for each bZIP in the two fusion conformations. Levels not connected by a common letter are significantly different (α = 0.05).

Unraveling the interaction patterns of a high number of bZIPs represents a challenging task, as the number of possible combinations to be analyzed is the number of monomers in study squared. Protein-fragment Complementation Assays (PCA) allow the high-throughput analysis of *in vivo* protein-protein interactions providing additional information on the subcellular localization and the strength of the interaction [36–39]. Specifically, the BiFC assay provides a higher sensitivity for weak and transient interactions, since the interacting proteins remain trapped by the reconstitution of the fluorescent protein [37, 40]. Therefore, we chose this method as bZIPs interactions were expected to be weak according to predictions and experimental data available [20, 41]. The BiFC assay was performed using the split Yellow Fluorescent Protein (YFP) system in transformed *Arabidopsis* protoplasts, and a double design was followed as advised [40], so that each bZIP was investigated as a fusion to both the N- and the C-terminal fragments of the YFP (here named SPN and SPC respectively). In other words, all combinations of two different bZIPs were tested twice (Fig 1A). Such a dual analysis reinforces the accuracy of the results, as it is less likely to obtain a same false positive (or negative) result in both fusion conformations. The emitted fluorescence was measured by flow cytometric analysis, because it provides a quantitative approach that improves the discrimination of the false positives [37, 42]. For each combination at least three biological replicates were analyzed, each one with a minimum of 30,000 cells screened. The fluorescence level was assessed as a binomial distribution of the fluorescent cells versus the non-fluorescent cells and a generalized linear mixed model (see methods) was fitted on the whole dataset in order to provide a single BiFC mean for each bZIP combination (S2 Table).

To substantiate the acquired data, the reciprocity between the results generated from equivalent pairs of bZIPs (i.e., the two split YFP fusion conformations) was evaluated. Eight out of the 120 comparisons tested were significantly different, meaning that the two fusion conformations lead to different outcomes (Fig 1B). However, an inspection of these disparate combinations revealed that six of them involved the protein bZIP63 (C class). To understand why bZIP63 concentrates so many dissimilar measurements, we compared the marginal means of the BiFC scores (i.e., the mean of all the BiFC scores involving a given fusion protein). A significant difference was found only for bZIP63-SPN in comparison to any other bZIP protein fusion, meaning that the measured BiFC scores involving this bZIP are inconsistent with the rest of the dataset (Fig 1C). These disparate measurements are probably due to the effect of the different arrangements of the fluorescent protein fragments influencing the signal properties of the BiFC complex [40]. Still, we wanted to determine whether they represent just a shift in the fluorescence intensity, without altering its dimerization pattern. Therefore, the similarity of the interaction profiles between the two bZIP63 conformations was assessed calculating the Pearson’s correlation coefficient, which resulted in a positive and significant correlation (R = 0.835 and p-value <0.001). This indicates that the disparate measured BiFC scores are due to an enhanced fluorescence signal, and not a modification of the relative magnitudes of the different bZIP combinations. So, reciprocity of the bZIP63 values was confirmed. However, the disparity in the values range still invalidated the use of the bZIP63-SPN measurements for comparison purposes, necessitating the exclusion of bZIP63-SPN in some of the analyses below.

### The clustering of the interaction matrix reveals a link between the bZIP dimerization and function

**Fig 2:**
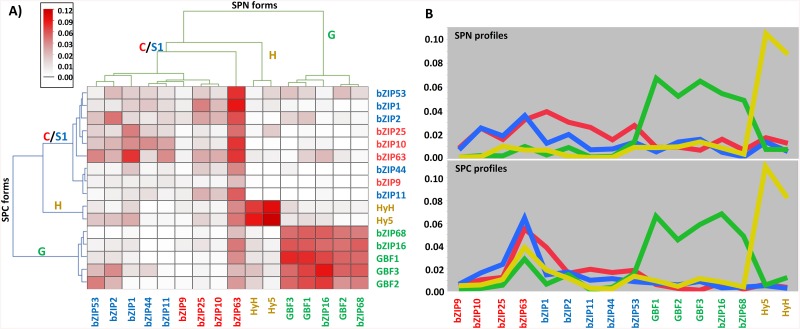
Different bZIP dimerization patterns. **A)** Clustered heat map. The clustering was performed independently for the SPN and the SPC conformations, resulting in three main clusters that correlate to the different bZIP groups in the Jakoby classification, which are indicated by their group defining letter. The main discrepancy between the two outputs is the positioning of bZIP63: although it belongs to the cluster containing the C- and S1-bZIPs in both cases, it forms a single-leafed clade in the bZIP63-SPN conformation, which is due to the discordant values of bZIP63-SPN already mentioned in the text. The increasing intensity of the red color corresponds to the higher BiFC scores, according to the scale. **B)** Interaction profile plot. The graph represents the averaged BiFC scores for each bZIP class: C, G, H, and S1.

In order to uncover similarities and differences among the interaction patterns of the 16 bZIPs, the result of the BiFC assay was summarized as a 16x16 matrix, representing the 120 reciprocal bZIP combinations plus the 16 homodimers. The rows and columns in the matrix containing the adjusted means calculated from the fitted model (see methods) were ordered by hierarchical clustering. This was done for the two fusion conformations independently, both delivered mostly equivalent outcomes, resulting in three main clusters: the first cluster comprised the G-bZIPs, the second the H-bZIPs, and the third the C- and S1-bZIPs together (Fig 2A). The G-bZIPs form the most dissimilar group, according to the linkage distance and the sharp contrast observable in the heat map between the intense scoring intensity of the homotypic dimers (formed by two G-bZIPs monomers) and the faint heterotypic dimers (i.e., formed by one G-bZIPs and a bZIP from another group). Similarly, the H-bZIPs also exhibit an abrupt difference between the homotypic and heterotypic dimers. Overall, the C and S1-bZIPs exhibit a homogeneous profile of low BiFC scores. Still, within the cluster comprising the C- and S1-bZIPs a tendency towards heterotypic interactions is recognizable in the interaction profile plot (Fig 2B). The C-bZIPs averaged BiFC scores are higher in the interactions with the S1-bZIPs and, conversely, the S1-bZIPs exhibit higher values when interacting with the C-bZIPs. Thus these two groups exhibit complementary profiles in spite of being in the same dendogram clade. The H-bZIPs dendogram clade is closer to the C-and S1-bZIPs as they have predominantly flat interaction profiles.

Hence the *in vivo* dimerization analysis revealed the same arrangement as the Jakoby classification highlighting an interesting viewpoint on bZIP function, given that this classification is not based on the LZ, but on the character of the DNA-binding and other functional domains [11]. Therefore, these results provide a link between a given dimerization pattern and particular DNA-binding and functional properties.

### bZIPs with alternative dimerization pattern exhibit different dimerization strengths

**Fig 3:**
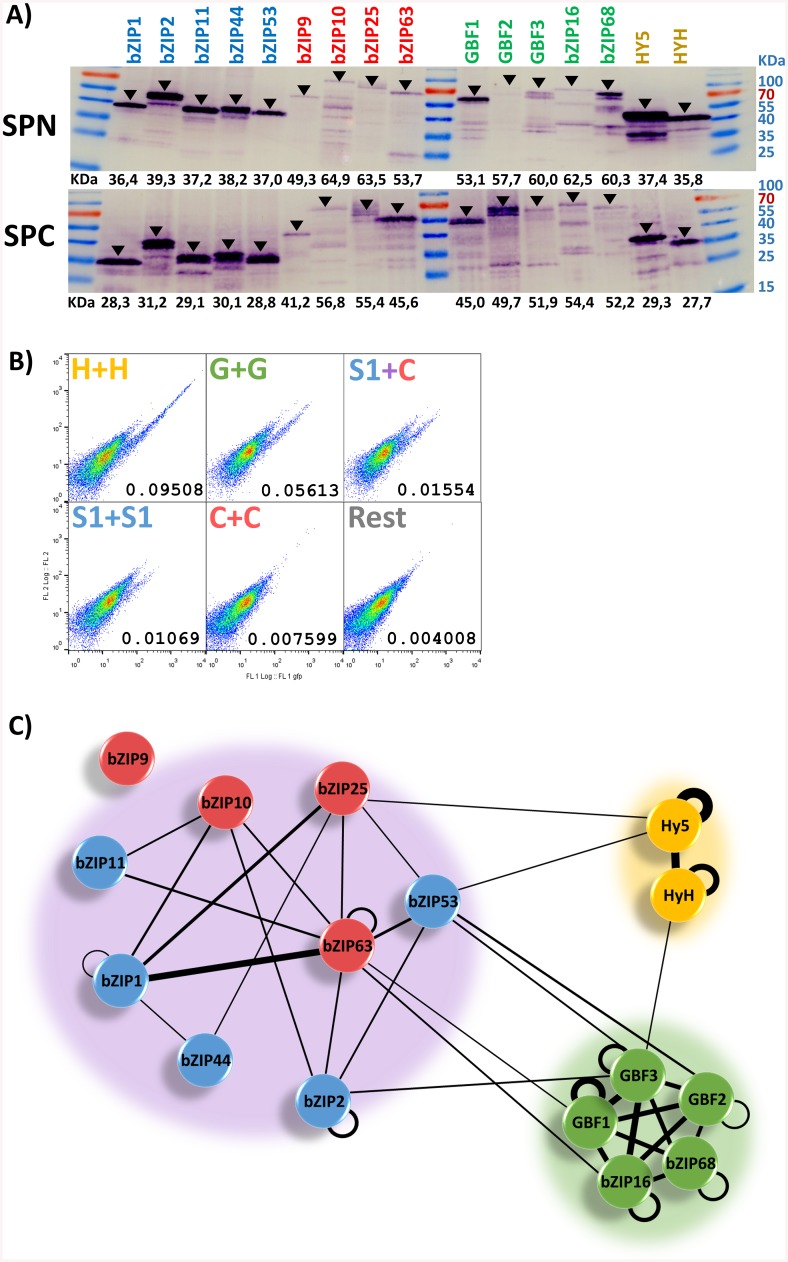
Analysis of the dimerization strength. **A)** Western blot of protein extracts isolated from protoplasts transiently transformed with only one bZIP. Four transformation reactions were pooled and 7 μg of total protein was used in SDS-PAGE. The SPN and SPC conformations were detected with anti-Myc or anti-HA antibodies, respectively. Arrows indicate the bands corresponding to the expected proteins. All bZIPs display an apparent molecular weight greater than the theoretically calculated as previously reported, which has been related to protein disorder [43]. **B)** Flow cytometry outputs representative for each of the six groups analyzed and their adjusted means. **C)** The three bZIP networks identified by analyzing the BIFC scores in a binary fashion with a cutoff of 0.01. The width of the connecting lines is indicative of the BiFC score mean for the bZIPs connected. Homodimers represent only one value, as they cannot be tested in alternative conformations. Combinations including bZIP63 represent only one value, as bZIP63-SPN measurements are omitted. All values are relative to the Hy5 homodimer, which exhibits the highest BiFC score.

As introduced above, complementation assays can be indicative of the strength of the interaction when a quantitative approach is used as the detected signal is positively scalar to the amount of reporter protein reconstituted which in turn depends on the affinity, and thus the half-life of the interacting complex [36–38, 40]. In our results, we found pronounced differences among the calculated BiFC scores, raising the question whether these are related with the affinity of the interacting bZIP pairs. Therefore, we inspected the levels of the fusion proteins in a western blot to assess whether the different BiFC scores could result from a differential expression of certain fusions (Fig 3A). Unequal protein amounts were detected in the transiently transformed protoplasts and these were consistent in both fusion conformations: the H- and S1-bZIPs led to bulky protein bands, whereas the C- and G-bZIPs’ bands were much fainter. However, these variations in the detected proteins do not explain the differences measured in the BiFC scores, for instance, the G-bZIPs produce very high BiFC scores but only low protein levels and, conversely, the S1-bZIPs result in low BiFC scores but huge amounts of detectable protein. Moreover, each bZIP lead to differential signal strengths as the dimerizing partners alternate, so the differences in the measured BiFC scores cannot be attributed to unequal expression of the fusion proteins and, therefore, the BiFC scores are likely to represent a distinctive feature of each bZIP pair tested, so that they can provide a reasonable hint on the affinity of the interaction.

To compare the BiFC scores, bZIP63-SPN values were omitted and the interaction matrix was divided into groups, so that each group included combinations of bZIPs with similar interaction patterns (Fig 2B, S1 Fig). Accordingly, two groups were formed for the G- and H-bZIPs homotypic dimers, while the group comprising the C- and S1-bZIPs was further split in three groups, two of them with the homotypic dimers for each bZIP group (only C-bZIPs and only S1-bZIPs) and the third one comprising the mixed C/S1 heterodimers. In addition, an extra group was formed as a “catchall” to include the remaining combinations between bZIPs which did not cluster together. This last group was the largest one with 132 bZIPs combinations and resulted in the lowest BiFC mean, as most of the negative interactions fell in this group. All group pair comparisons resulted in significant differences (S3 Table). The two groups with the G- and H-bZIPs displayed much higher means than the three groups covering the C- and S1-bZIPs. Within the latter, the heterotypic heterodimers exhibit the highest BiFC score mean, followed by the homotypic S1-bZIP dimers, and the homotypic C-bZIP dimers having the lowest values. This ordering is coincident with a previous quantitative study reporting a higher affinity between heterotypic heterodimers between C- and S1-bZIPs, reflecting their preferential formation [21], thereby substantiating the use of the quantitative BiFC scores as an indicator of the interaction affinity. Based on that, the differences here revealed indicate a much higher stability of the homotypic C- and H-dimers than any dimer arisen among the C- and S1-bZIPs dimers.

### The interaction map shows the 16 bZIPs self-assemble into three networks

To further dissect the data, we generated an interaction map of the 16 bZIPs. For that purpose, a threshold was set to define the BiFC score from which interactions are to be considered as positive. This threshold was set to a value of 0.01 (BIFC-score-normalized) based on the results of the group means comparisons (S3 Table). Our criterion was to consider positive signals as those values with the minimum of their standard error interval lying above the threshold in a consistent manner for both fusion conformations (example in S2 Table). In total, we identified 43 positive interactions out of 136 combinations tested. These are arranged as a graph and forming three main networks of interacting bZIPs in consonance with the clustering output (Fig 3C). The largest network is the so called C/S1 network, within which the heterotypic interactions (involving one bZIP from each group) are more abundant than the homotypic combinations, in agreement with their preferential heterotypic heterodimerization previously described [21, 22]. Here, we identified fewer interactions than in other reports, and strikingly none for bZIP9 (C class). These differences probably arise from different criteria, as we considered the lack of reciprocity as an excluding criterion, making this study more conservative. In contrast to bZIP9, bZIP63 (C class) establishes the most interactions, being able to dimerize with almost all the members in the C/S1 network.

The other two networks consist of only homotypic dimers, either of G-bZIPs or H-bZIPs, therefore they are named the G and the H networks, respectively. Members within these two networks dimerize with themselves with an absolute lack of specificity, consistent with other observations [44–47]. Beyond the three networks, there is a substantial lack of interactions between members belonging to different groups, reflecting the high specificity of the bZIP dimerization consistent with other reports [20, 41]. Only few sporadic dimers are identified interconnecting the different networks. However, a physiological role for such inter-network dimers has not been described so far and appears to be uncertain in light of our further data presented below.

### The bioinformatic analysis of bZIP LZs suggests the involvement of varied forces in bZIP dimerization

**Fig 4:**
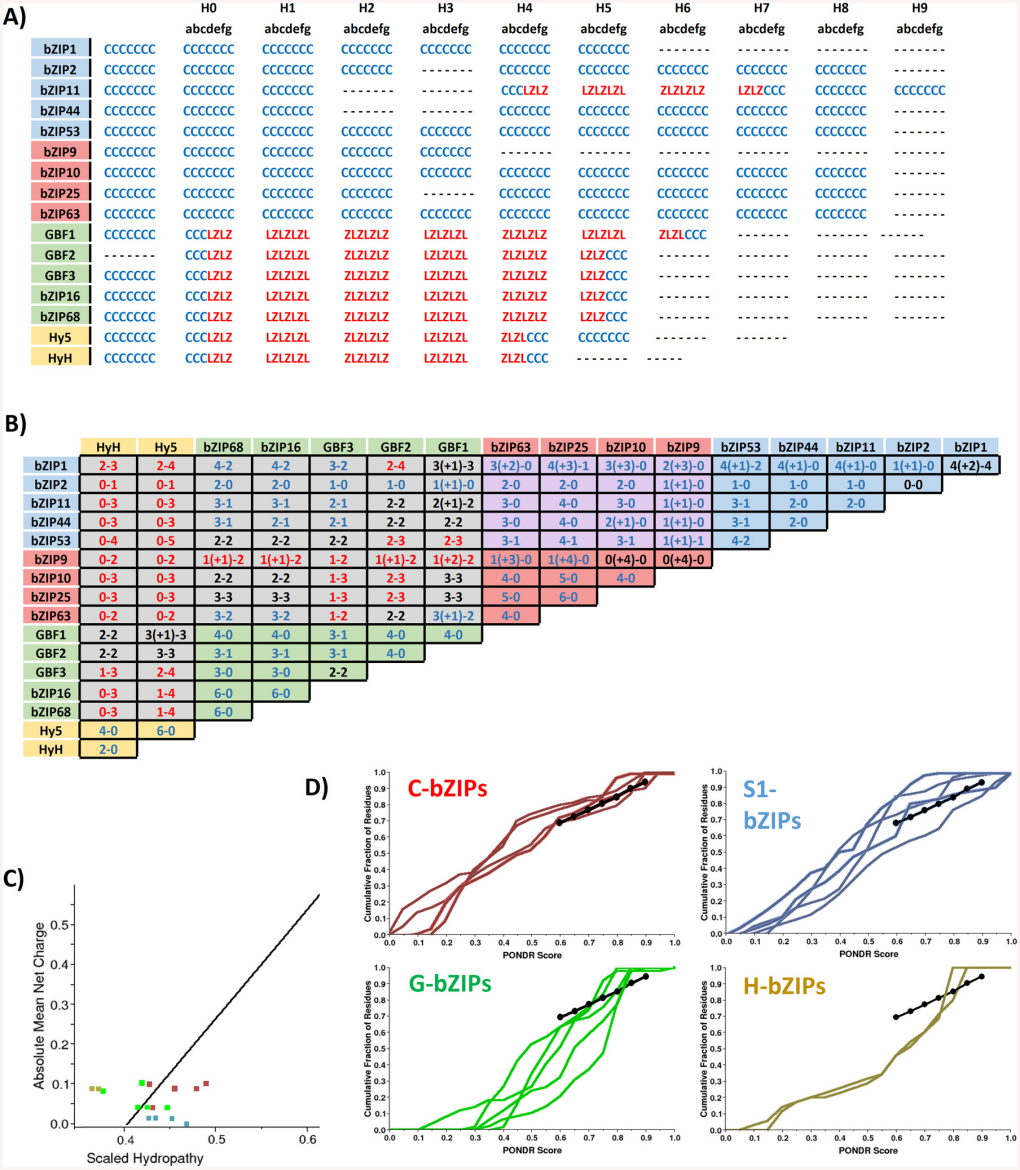
Bioinformatics-based analysis of the bZIPs. **A)** Prediction of the coiled coil (CC, blue) and LZ (LZ, red) motifs for the bZIP domains of the 16 bZIP proteins by the 2ZIP software. The heptads are numbered based on Deppmann *et al*. [19]. Predictions from the COILS program are found in S2 Fig. **B)** Electrostatic interactions calculated between pairs of bZIPs. The numbers indicate the attractive—repulsive interactions between **e**-**g** positions. Interactions that lay outside of the predicted coiled coil region in at least one of the monomers are indicated in parenthesis. Combinations with a positive balance between attractive and repulsive electrostatic interactions are indicated in blue, negative in red, and neutrally balanced in black. The background is colored according to the different bZIP networks. The number of interactions was calculated with DrawCoil 1.0 (www.grigoryanlab.org/drawcoil). **C)** Charge-hydropathy plot to predict protein disorder, in which the line represents the boundary between disorder (above) and order (below). **D)** Cumulative Distribution Functions on the PONDR VL-XT algorithm are shown for C-bZIPs (blue), S1-bZIPs (red), G-bZIPs (green), and H-bZIPs (yellow). The more concave the line is below the boundary represented by the dotted line, the more disordered the protein. Conversely, the more convex it is above the boundary, the more ordered. In C) and in D) the sequence analyzed spans from heptad 0 to heptad 5 in H-bZIPs, to heptad 7 in G-bZIPs, and to heptad 10 in C- and S1-bZIPs.

*In silico* prediction of bZIP proteins is based on the identification of the bZIP signature as a whole, including the BR and the LZ domains [11, 19, 48]. However, because the BR is highly conserved, it could have a higher impact than the LZ in the bZIP annotation process, so that singularities in the LZ could have been overlooked. The LZ motifs of the 16 bZIPs analyzed here were predicted to be different in length: the C- and S1-bZIPs LZs should be longer, with up to 10 heptad repetitions, while the G- and H-bZIPs would feature short LZs with 5 and 4 heptads respectively [11, 19, 20]. Therefore, we analyzed whether the predicted variability in the dimerization strength could be related to the observed differences in the LZ lengths. We estimated the probability of coiled coil and LZ formation with the 2ZIP and COILS programs [49, 50]. Unexpectedly, in the C- and S1-bZIPs, the sequences assumed to be LZs are not predicted to form such motifs, and only a short LZ motif is predicted for bZIP11 (S1 class) between heptads four and seven, according to previous heptad numbering [19]. A coiled coil motif is still predicted in all these sequences, but it does not span along all the putative heptads. The longest predicted coiled coils are those of bZIP63 (C class) and bZIP53 (S1 class), with eight heptads, whereas the shorter one is calculated for bZIP9 (C class), with only three repeats. Interestingly, for bZIP25 (C class) as well as for bZIP44, bZIP2, and bZIP11 (all S1 class) two separated coiled coil motifs are computed, suggesting that these bZIPs carry a bipartite interacting motif or alternate conformations of a singular motif (Fig 4A). These intriguing results reveal a greater heterogeneity in the bZIP dimerization motif than has been anticipated.

A closer observation of the amino acid sequences revealed that several of the **d** positions are not occupied by a leucine residue which is the most stabilizing amino acid in this position and hallmark of the LZ motif. Specifically, these are the **d** positions in the second heptad of the S1-bZIPs and in the third and fourth heptads of any of the C- and S1-bZIPs (S2 Fig). Instead, the **d** positions of these heptads carry amino acids with low stabilizing properties, or even destabilizing, such as the β-branching valine and isoleucine, threonine or alanine [17, 51, 52]. Likewise, the **a** positions in these heptads do not bear the most stabilizing amino acids, which would be β-branching. Moreover, the second heptad of bZIP10 and bZIP25 (both C class) carries an histidine residue which is highly destabilizing [52], and the **a** position of the fourth heptad of all the C- and S1-bZIPs, except for bZIP1 (S1 class), is occupied by tyrosine or phenylalanine, two bulky aromatic amino acids which are rarely found in this position that can produce inter-helical clashes inducing local structural disturbances in the supercoil structure [53]. In addition, some of the solvent-exposed **b**, **c**, and **f** positions along these three two heptads are occupied by highly hydrophobic amino acids such as isoleucine and valine, which are likely to be destabilizing. Altogether, the composition of these three heptads in the C- and S1-bZIPs are likely to weaken the hydrophobic core strength resulting in a packing defect. In contrast, the sequences of the G- and H-bZIPs follow the canonical composition of the LZ with the **d** positions held by leucines and abundant stabilizing residues in the **a** positions, beside that G-bZIPs can form disulfide bridges [13, 14, 46, 52, 54]. Accordingly, all the G- and H-bZIPs are predicted to form LZs along the entire postulated sequence and, consequently, stable and tightly-packed dimers are expected.

Next, we calculated the electrostatic interactions between **e** and **g** positions along the stipulated LZ sequences to assess whether they can play a role determining the dimer stability and, thus, explain the differences observed in the BiFC scores (Fig 4B). In general, all dimers of the three networks are able to form several stabilizing salt bridges and their number ranges from zero to six with no unique distinction in the three networks, so that the stronger interactions in the G and H networks cannot be explained by a greater absolute number of salt bridges. On the other hand, some of these dimers result in repulsive **e**-**g** pairs, which is proposed to be the major excluding force in the partner selection [55]. Nevertheless, in many cases there is only one repulsive pair and several attractive pairs, so that it cannot be excluded that the attractive forces overcome the repulsive ones. The destabilizing **e**-**g** pairs arise in the G and C/S1 networks similarly, so they do not *per se* explain the different dimerization strengths. However, due to the differences in length of their coiled coil motifs, the dimers in the G and H networks form about twice as many salt bridges relative to the number of heptads compared to the C- and S1-bZIPs, which might substantially improve the dimer stability. In addition, the electrostatic interactions are not homogeneously distributed all along the coiled coil motif in the C/S1 network dimers, but instead they accumulate towards the end of the motif after the sixth heptad. Conversely, in the dimers of the G and H networks, the salt bridges cover the core region of their LZs, supporting the idea of their higher stability.

Another aspect reported to affect the dimerization dynamics of the bZIPs is the existence of different degrees of disorder in the LZs. Since the bZIP dimerization is assumed to be coupled to the folding in a two-state transition model from unfolded monomers to the dimeric coiled coil [56], the presence of ordered regions such as preformed secondary structures would reduce the entropic cost of the folding process, lowering the transition state free energy and, thus, facilitating the conversion between the different thermodynamic states [43, 57, 58]. Therefore, we analyzed whether the members of the different bZIP networks exhibit distinctive degrees of disorder. The presence of disorder was estimated by different PONDR algorithms, charge-hydropathy plots and cumulative distribution functions [59], resulting in large disordered regions being predicted in all of the 16 bZIPs. But, narrowing down the region of interest to the dimerization motif, the coiled coil sequences exhibit meaningful differences in their degree of disorder among the bZIPs of different networks. These are predicted to be extensively unstructured for the H-bZIPs and two G-bZIPs (GBF1, bZIP16); and to a lesser extent for the rest of the G-bZIPs. Conversely, the corresponding regions in the C- and S1-bZIP are calculated to be rather ordered (Fig 4C and 4D and S2 Fig). Accordingly, the lower degree of disorder in the C/S1 network bZIPs reduces the entropic penalty associated with the dimerization, and thereby facilitates the monomer-exchange within this network by a similar mechanism as proposed for Opaque-2 bZIP in *Coix* plants [58].

### An extensive reporter-gene assay reveals functional redundancy among the members of the same network

Recall, dimerization is assumed to be a central mechanism regulating the bZIP activity since the combination of different monomers is hypothesized to generate dimers with unique properties as to their DNA-binding and their transactivation potential. Until now, only interactions between bZIP53 (S1 class) and the C-bZIPs have been studied systematically [60]. To get more insight into this assumption, the transactivation potential of different bZIP dimers was systematically analyzed on four selected promoters previously reported to be directly targeted by bZIPs: *PROLINE DEHYDROGENASE* (*PDH*), *ASPARAGINE SYNTHETASE 1* (*ASN1*), *CATALASE2* (*CAT2*), and *RUBISCO SMALL SUBUNIT 1a* (*RBCS1a*) genes [23, 44, 46, 61–63]. The sequences comprising 1.5 kbp upstream of these genes were cloned into the pBGWFS7 vector to control the expression of a GUS reporter gene [33]. The resulting constructs were transformed into protoplasts along with plasmids encoding for the *bZIP* effectors and for a firefly *Luciferase (LUC)* gene for normalization purposes (see methods). Initial set up tests revealed that, unlike the promoters of *PDH*, *CAT2* and *RBCS1a*, the *ASN1* did not lead to basally detectable amounts of GUS activity under our conditions. However, the expression of *ASN1* was induced by certain bZIP combinations.

**Fig 5:**
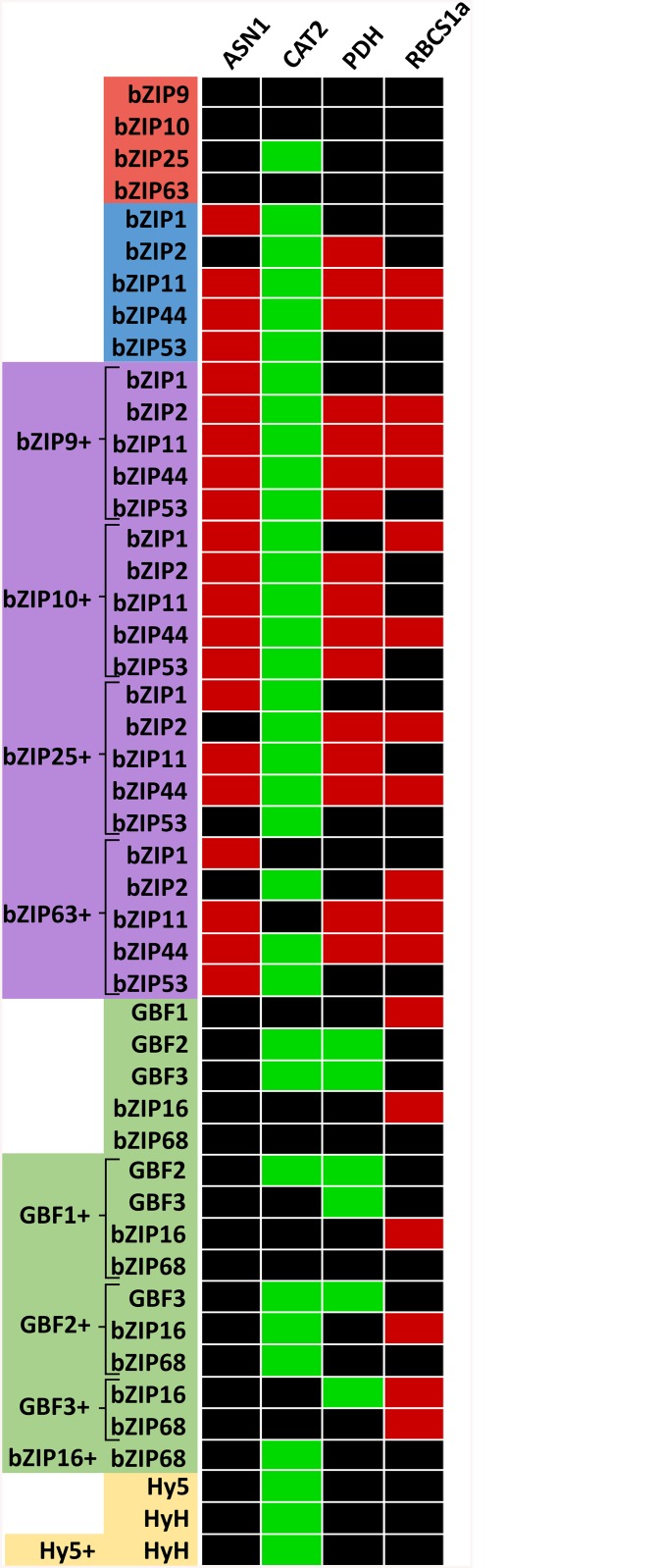
Promoter-reporter transactivation activity of the 16 bZIPs on four different known bZIP target genes. The transactivation potential of different bZIP combinations on the promoters of *ASPARAGINE SYNTHETASE 1*, *CATALASE 2*, *PROLINE DEHYDROGENASE*, and *RUBISCO SMALL SUBUNIT 1*. Red color indicates significant increase, while green color represents significant decrease. Black squares indicate no significant change.

The GUS assays were carried out using the 16 bZIP effectors individually or together with a second bZIP, considering all the G and H network combinations and the heterotypic dimers from the C/S1 networks, as these exhibit the strongest interactions within this network. The results were analyzed for each promoter separately using the calculated mixed model (see methods). The hypothesis tests were conducted between each of the bZIPs combinations and the reference control (R), which in our case was the empty effector protein expression vector. The result of the test indicated whether the measured GUS activity in the assayed bZIP combinations is significantly different to the background level of each promoter (Fig 5, S4 Table and S3 Fig). The interpretation of the results for the single effectors can be made straightforward since for each bZIP (A) there are only two possible outcomes: significant (A≠R) or non-significant (A = R). However for the combinations of two effectors (A and B) up to three dimer populations can arise (AA, AB, and BB) and they can interplay differently with the multiple cis-elements present in the promoter, hence it cannot be distinguished whether the measured GUS activity results from the separate action of the two homodimers, the formation of heterodimers, or a mixture of both situations. The result of the test is, therefore, interpreted comparing the effects of the two single effectors (A and B) with their mixture (M) and focusing only on whether there is a significant change or not, while not contemplating other possible outcomes such as additive or subtractive effects. Based on that, we observed four different outcomes in the assay: i) **no change of state** when the single effectors produce the same outcome as their mixture (A = B = M), ii) **synergy** when there is a significant change in the mixture, but not in any of the bZIPs as single effectors (A = B = R≠M), iii) **dominance** occurs when the single effectors lead to different outcomes (one results in a significant change, but not the other), so that one of them prevails over the other in the mixture. In this case, we name it **effective dominance** when the mixture results in a significant change (A = R and B,M≠R; or B = R and A,M≠R), and **idle dominance** when there is no significant change in the mixture, yet a change was detected for one single effector (A≠R and B,M = R; or B≠R and A,M = R). Remarkably, there are no combinations in which both single effectors result in significant changes, but one increasing the expression and the other decreasing it.

The *ASN1* promoter is known to be activated by the S1-bZIPs [24, 25] and it is also induced in our experiments by bZIP1, bZIP11, bZIP44, and bZIP53 as single effectors. Conversely, none of the other bZIPs induced a significant change as homodimers. As a general trend, effective dominance of the S1-bZIPs over the C-bZIPs was observed, as the co-transformation of these with the C-bZIPs likewise resulted in significant changes in *ASN1*. In the case of bZIP2 (S1 class) synergy was observed because it did not induce a significant change as single effector but did so when combined with C-bZIPs. No changes in *ASN1* expression were detected with respect to co-transfection with the G- and H-bZIPs. The lack of basal expression for this promoter under our conditions could have masked any repressing effects for those bZIPs which did not display an increase in expression.

The *CAT2* promoter was reported to be down-regulated by the G class bZIP GBF1 [63], while none of the other bZIPs have been analyzed so far. In this assay, GBF1 did not result in a significant reduction of *CAT2* expression. Among the other G-bZIPs, only GBF2 and GBF3 were able to significantly reduce the expression of this promoter. In the G-bZIP combinations, GBF2 resulted in effective dominance over the rest of G-bZIPs and, conversely, GBF3 led to idle dominance. Regarding the C/S1 network, the S1-bZIPs as well as bZIP25 (C class) reduced the *CAT2* driven GUS expression, but not the rest of the C-bZIPs. Again, effective dominance of the S1-bZIPs over the C-bZIPs was observed with the exception of bZIP63. Lastly, H-bZIPs decreased *CAT2* expression, yet there was no change of state in combination.

The *RBCS1* promoter carries G-box sequences that are bound by the G- and H-bZIPs [28, 44, 64]. In our experiments, the H-bZIPs induced no significant changes, whereas among the G-bZIPs only GBF1 and bZIP16 were able to stimulate the transcription. These results are inconsistent with the reported roles of GBF1 and Hy5 as repressor and activator respectively [28]. These disagreements could be explained by the functional dependence on the cellular context reported for the G- and H-bZIPs, for which an interplay with other cis- and/or trans-elements is believed be a functional requirement [64–66]. Indeed, it was demonstrated that neither GBF1 nor Hy5 are sufficient to exert their function alone. Besides that, in the G-bZIP combinations there was effective dominance of bZIP16 over GBF2 and GBF3, but idle dominance of GBF2 and GBF3 over GBF1. Among the C- and S1-bZIPs, whose effect on this promoter has not been tested so far, only bZIP11 and bZIP44 (both S1 class) activated the expression as single effectors, and they exhibited effective dominance over the C-bZIPs. In addition, there was synergy between bZIP2 (S1 class) and all the C-bZIPs except bZIP10, which in turn was the only C-bZIP that showed synergy with bZIP1 (S1 class).

The *PDH* promoter is activated by the S1-bZIPs according to several studies, although important contradictions have been reported regarding which of the S1-bZIPs induce the expression and which do not [24, 25, 60, 61]. In our assay, we found significant activation of this promoter by bZIP2, bZIP11, and bZIP44 as single effectors; and they showed again effective dominance over the C-bZIPs. Significantly, among the G-bZIPs, GBF2 and GBF3 resulted in a significant down-regulation, thus displaying an opposite role to the S1-bZIPs.

To sum up these results, a trend towards a functional consistency among the members of the same network is evident, in the sense that those bZIPs which induce significant changes do so in the same direction (increasing or decreasing) and to a similar extent. In other words, no coexistence of activating and repressing functions in the same network is observed for a given promoter. However, not all the members of a network result in significant changes, suggesting that the different bZIPs have differentiated functional requirements. Such conditional activation seems to be a core feature in bZIP function that is mediated by further activating factors or certain conditions such as osmolarity or light conditions for example [25, 61, 64–66].

### The observed bZIP networks likely function ubiquitously *in planta*

**Fig 6:**
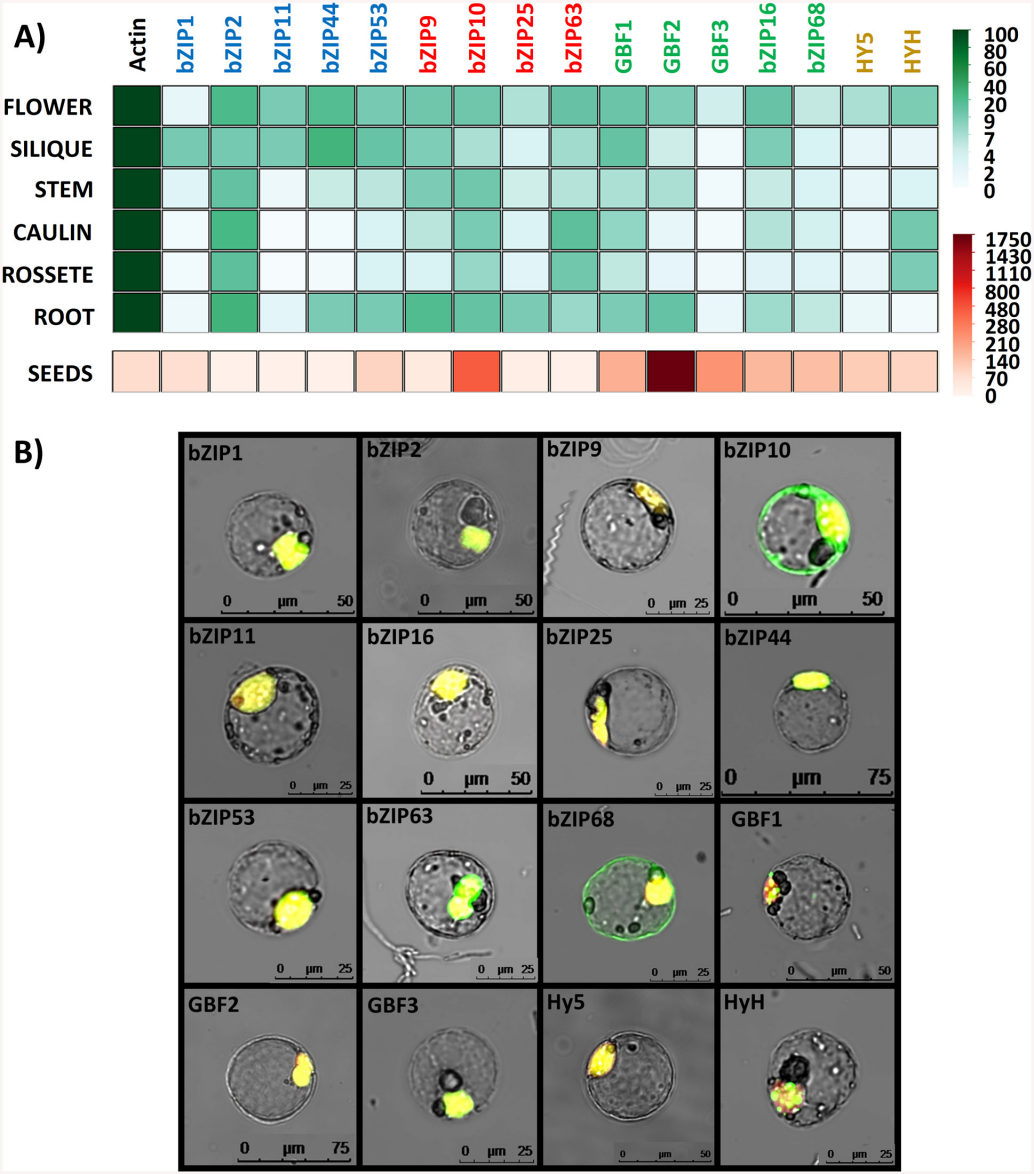
Analysis of the bZIP expression and cellular localization. **A)** Expression analysis of the 16 bZIPs in a 6 week-old plant. Values are expressed in percentage relative to the *ACTIN2* and were calculated as 2^ΔCt. **B)** Subcellular localization of the 16 bZIPs in transformed protoplasts. The bZIPs were expressed as GFP fusions (green) along with a plasmid carrying a mCherry gene fused to a NLS for nuclear staining (red). Represented are the merged pictures (originals as S1 File).

Finally, it was of interest to determine whether the members of the same network are co-expressed together *in planta* as a prerequisite for interaction. Therefore, the expression of all 16 bZIPs was analyzed by qPCR in different plant organs: namely seeds, roots, rosette leaf, cauline leaf, stem, flower and siliques (Fig 6A). According to our data, most of the bZIPs exhibit similarly low levels of expression which are approximately one to two orders of magnitude below *ACTIN2* levels. In seeds, bZIPs display expression levels closer to *ACTIN2* due to decreased expression of this gene in this part of the plant. These measurements are in agreement with the data from public microarray repositories gathered in the Genevestigator database (S4 Fig). Besides that, there are noticeable variations in the expression levels of some bZIPs among the different tissues. For instance, the S1-bZIPs bZIP1, bZIP11, and bZIP44 display very low levels in rosette and cauline leaves, as previously reported [67], and bZIP10 (C-class) and GBF2 (G class) are especially increased in seeds. Conversely, other bZIPs barely change their expression among tissues, including the homogeneously low levels of GBF3 (G class), or the ubiquitous high levels of bZIP2 (S1 class) in agreement with previous descriptions [68].

Another important aspect considered is whether or not the bZIPs are localized to the nucleus in order to be able to exert their function as transcription factors. To determine the subcellular localization of the bZIPs, the 16 bZIPs were expressed as GFP fusion proteins in transiently transformed protoplasts (Fig 6B and S1 File). While the majority of them were found exclusively in the nucleus, a double localization, in the nucleus and in the cytoplasm was observed for bZIP68 (G class), as well as for bZIP10 (C class), congruent to a previous report [69]. In addition, GBF1 (G class) and HyH (H class) formed nuclear speckles, a phenomenon that has been associated in plants with light signaling [70].

These findings indicate that, on the one hand, the three bZIP networks can occur *in planta*, as most bZIPs of each network are expressed simultaneously and are localized in the nucleus (at least partially). On the other hand, the variations in their transcript levels and cellular localization indicate that the abundance of particular bZIPs can be specifically regulated.

## Discussion

### The wider perspective on the bZIP dimerization and function suggests that *Arabidopsis* bZIPs operate as functional units

Inspired by the somatic recombination of immunoglobulins, the observation that many eukaryotic transcription factors form homo- and heterodimers was soon assumed to be a combinatorial mechanism generating a diversified repertoire of functions from a restricted set of regulatory elements [71]. This notion is applied especially to the bZIPs, given that they are obligated dimers and that different bZIP monomers have been reported to possess distinctive DNA-binding affinities and transactivation capabilities. However, this assumption depends on the capability of the bZIPs to form dimers, so that in a scenario in which dimerization is highly restricted, which has been predicted for *Arabidopsis* bZIPs; such a combinatorial mechanism would be very limited. Available *in vivo* studies on bZIPs normally deal with few combinations of related monomers, lacking the comparative perspective required to give insight into the relationship between the bZIP dimerization and function.

The broad analysis of the bZIP dimerization and function presented here brings a new perspective on the manner *Arabidopsis* bZIPs operate: i) bZIPs are organized in small, rather independent networks. ii) Within each network, bZIPs interact promiscuously between themselves. iii) The analyzed properties of the bZIPs such as dimerization strength, transcriptional effect, functional relationships between monomers, and structural features of their LZs are characteristic for members of the same network, but are distinctive for each network.

**Fig 7:**
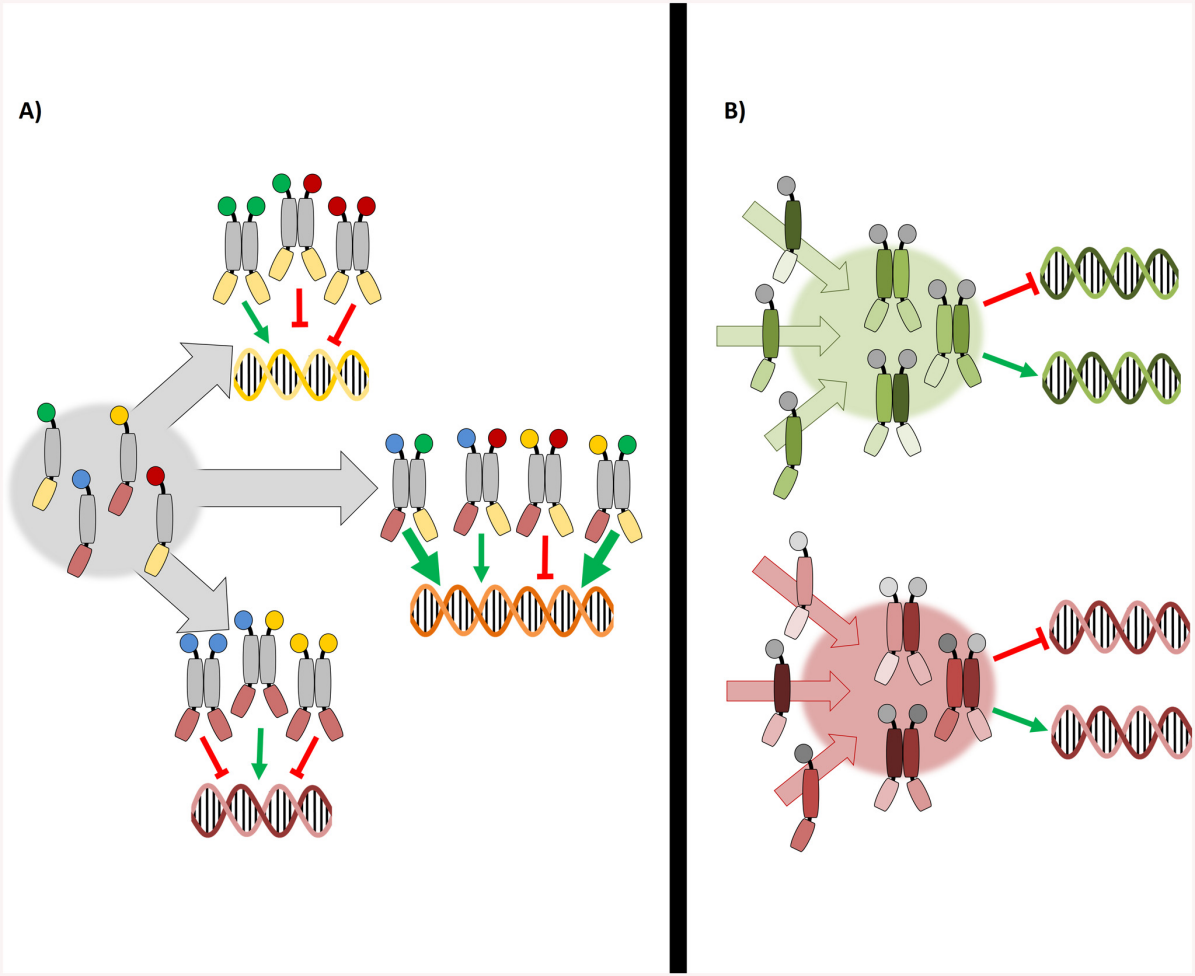
Model of action for the bZIP networks. Diagram in which bZIP monomers are depicted highlighting the three main elements defining their function: the DNA binding (bottom part), the dimerization (middle part), and the transactivation (upper circle). **A)** Classical view of the bZIP dimerization according to which the function lies on the bZIP dimer. Interacting monomers have varied properties regarding transactivation and DNA binding, so that the heterodimerization generates diverse functions. **B)** Proposed model of bZIP networks as functional units. In this case the function is a property of the network itself, since the monomers already share similar transactivation and DNA binding properties. The role of the heterodimerization here is to integrate the different inputs which impinge on the expression of certain bZIPs or regulate their function.

An important consequence of these considerations is that the possibility to generate dimers with new and unique qualities through the heterodimerization is strongly limited, as bZIPs that can dimerize already share a high degree of functional redundancy. Hence, no fundamental changes in the bZIP function are expected directly due to intra-network heterodimerization. This prompts the notion that the bZIP networks operate as functional units. According to that concept, the observed variations in the transcript levels or in the transactivation activities among the bZIPs of the same network serve as varied input possibilities, so that the bZIP heterodimerization operates by integrating multiple inputs to maintain a robust output function, instead of regulating the dimer composition to generate specific functions (Fig 7).

### The differential length of the coiled coil motif suggests a novel mechanism defining the bZIP dimerization specificity

**Fig 8:**
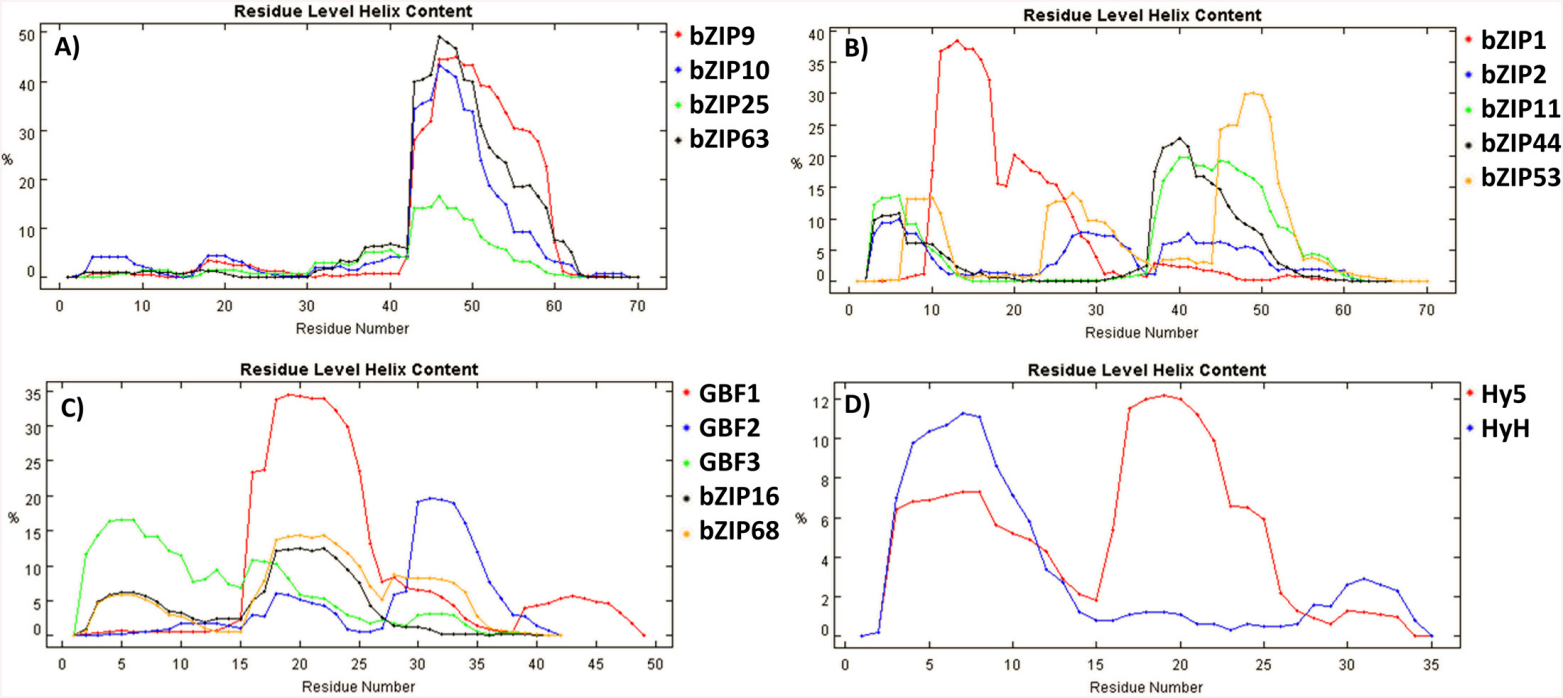
Heptad helicity. The helical content of the coiled coil sequences was estimated starting from heptad 0 with the AGADIR algorithm [76], assuming 278°K, pH 7, and 0.2 M ionic strength. Notice that the scales in the X axes are different, as the bZIPs analyzed feature different numbers of heptads repetitions. **A)** Results for the C-bZIPs along the 10 heptads. **B)** S1-bZIP along 10 heptads. **C)** G-bZIPs along 7 heptads and 8 heptads for GBF1. **D)** H-bZIPs along 5 heptads.

The electrostatic interactions between the **e**-**g** pairs are assumed to be the main forces defining the dimer specificity [20, 55], but they fail to explain some of the observed dimerization specificity in the 16 bZIPs studied, such as the combinations between G- and S1-bZIPs, which do not interact in spite of being predicted to establish exclusively attractive **e**-**g** pairs. Here, we propose a novel mechanism defining the bZIP dimerization specificity based on the differential positioning of certain heptads which can play more prominent roles in the dimerization, particularly those which can act as trigger modules. It is known that coiled coil interactions are often initiated from certain heptads with autonomous helical folding that act as trigger units [72–75]. The sequence of the trigger heptads varies among different proteins, so that there is no consensus sequence, and no trigger heptads have been yet defined for the bZIPs in this study. Still, in the C/S1 network bZIPs, the differential accumulation of salt bridges around the fifth to seventh heptads suggests that these could act as trigger heptads, despite the differences in the amino acid sequences between the two bZIP classes (S5 Fig). In addition, these heptads contain the two most important elements for the dimerization which are a strong hydrophobic core, formed by leucines in their **d** positions and some β-branched amino acids in their **a** positions, and a high helical propensity, according to the estimations of the helical content by the AGADIR algorithm (Fig 8).

As mentioned above, the C- and S1-bZIPs possess three heptads with a suboptimal amino acid composition in regards to the potential dimer stabilization. Significantly, while the proposed trigger heptads of the C- and S1-bZIPs are beyond the reach of the shorter LZs of the G- and H-bZIPs, the heptads with a suboptimal amino acid composition do match the LZs of the G- and the H-bZIPs. Hence, possible dimers between C/S1 network bZIPs and G- or H-bZIPs would result in strongly diminished hydrophobic and electrostatic forces. Conversely, dimers within the same network juxtapose their coiled coil motifs so that their heptads featuring the most stabilizing forces match together. Therefore, we hypothesized that the differential distribution of the forces driving the dimerization along the coiled motif in the C- and S1-bZIPs plays a pivotal role in their dimerization specificity, discriminating interaction between monomers whose trigger heptads do not match. This hypothesis provides a plausible explanation for the absence of dimerization between G-bZIPs and S1-bZIPs in spite of their positive balance of electrostatic interactions, as the calculated salt bridges in these dimers are predicted to accumulate on the second heptad, away from the trigger heptads of the S1-bZIPs.

### The architecture of the bZIP network

A previous study by Deppmann *et al*. on bZIP dimerization across different species concluded that plants and human bZIPs follow different strategies to achieve network complexity [20]. Accordingly, plant bZIPs are organized in many small isolated groups of non-selectively interacting bZIPs, whereas human bZIP form few large groups amply interconnected. The former represents a collection of independent networks, the latter a meta-network structure. However, the identification of several dimers in conflict with those predictions by Ehlert *et al*. questioned the accuracy of such predictions [21] and given that other studies on *Arabidopsis* bZIPs only focus on few closely related monomers, the organization of bZIP networks remained unresolved.

In this work we describe three interacting networks, which are identified as groups of bZIPs that extensively interact between themselves: the G-, H- and the C/S1 network, although C- and S1- belong to different classes of bZIPs. We also found several inter-network dimers connecting these three main networks, what could support a meta-network architecture. However, some features of the observed inter-network interactions weaken the confidence of their identification. First, the formation of the inter-network interactions involving the H-bZIPs (Hy5-bZIP25, Hy5-bZIP53, and HyH-GBF3) cannot be explained on the basis of current knowledge on the bZIP dimerization, as they would result in three or more repulsive **e**-**g** pairs and a double miss-pairing of asparagines in **a** positions, which is highly discriminating [12]. On the other hand, dimers connecting the G and C/S1 networks do match their asparagine residues in the **a** positions and bZIP2-GBF3, bZIP63-GBF1 and bZIP63-bZIP16, are predicted to have a positive balance electrostatic interactions, despite forming repulsive **e**-**g** pairs. However, although the formation of these pairs can be argued based on our *in silico* analysis, we cannot explain the observed degree of specificity allowing the interaction between just these bZIPs and not others (Fig 4B). For instance, dimers between bZIP2 (S1 class) and bZIP16 or bZIP68 (both G class) are not formed in spite of having an additional salt bridge compared to the dimer between bZIP2 and GBF3 (also G class) which does interact. In general, there is a big contradistinction between the abundant and unselective dimer formation involving all members of a given network, and the few and extremely selective inter-network dimers. Given the similarity in the coiled coil features among the members of a same network and their promiscuity, the fact that only few inter-network dimers arise is counterintuitive and their identification should be taken cautiously. The quantitative double-way BiFC assay performed and the statistical treatment bring enough confidence on the formation of these inter-network dimers under the conditions tested although they resulted in low BiFC scores. So the question revolves around whether the inter-network dimers actually occur under physiological conditions. In view of the high intra-network promiscuity, the formation of inter-network dimers would be in unfavorable competition with the intra-network ones, as the latter are expected to form preferentially according to the current knowledge. Therefore, we predict the formation of these inter-network dimers under physiological conditions, if any, will be rather limited, supporting the bZIP organization proposed by Deppmann *et al*. in small independent networks [19]. Notwithstanding, as it has already been noticed, plant bZIPs feature particularities in their coiled coil motifs (such as the heterogeneous lengths and the differential amino acid composition) that make them slightly different from bZIPs in other organisms [20] hinting at a more complex structure defining their dimerization. Therefore, the involvement of yet unknown factors defining the dimer specificity can still exist. Alternatively, we also contemplate that, perhaps, overexpression conditions of the assay forced transient formation of disfavored dimers and these were captured by the trapping effect of the BiFC system. While this is possible, we also keep in mind that we do not see a blanketed overexpression effect—the BIFC levels varied and were not correlated with total protein levels—yet this does not exclude it. In any case, as we only investigated 4 of the 10 bZIP groups, we feel that a further systematic *in vivo* investigation is still necessary to conclude about the architecture of the *Arabidopsis* bZIP network and address the singularities of the combinatorial mechanism of bZIPs in plants. In addition, we also consider that the dimerization specify could be modified by the cellular context, for instance bZIP11 and bZIP44 (both S1-bZIPs) carry a histidine residue in the **g** position of their third heptad, which can be protonated upon a pH decrease leading to alternative **e**-**g** interactions. Therefore, future research on bZIP dimerization should also consider testing the interactions under various conditions such as different pH values, osmotic strengths or oxidative levels.

### Are there alternative dimerization dynamics among the different bZIP networks?

The distinctive structural particularities of the coiled coil motifs predicted for each network, together with the observation of differences in the dimerization strength among the three bZIP networks prompted us to speculate about alternative dimerization dynamics within each network. Dimers within the G and H networks feature canonical LZ motifs with abundant stabilizing interactions but, at the same time, they are extensively disordered as monomers. This suggests that the more costly dimerization-folding process for these bZIPs remains secured once established. Indeed, three of the G-bZIPs carry cysteine residues in their **a** positions, allowing the formation of intermolecular disulfide bridges which should strongly stabilize the dimer [46]. Following this argument, dimers belonging to the G and H networks should be very stable and hard to break apart. Conversely, the bZIPs of the C/S1 network are less disordered, so their folding has less energetic requirements, and they contain heptads with high helical tendency that can act as triggers facilitating their dimerization. Simultaneously, C/S1 dimers feature fewer stabilizing forces, resulting in weaker dimers that can easily be separated back into monomers. Thus, in such dimers, the transition between the dimer-folded and the monomer-unfolded states entails lower energetic penalties, facilitating the transition between states in a similar manner to the proposed folding mechanism for the Opaque-2 bZIP in *Coix* plants [58].

Because dimerization defines function in bZIPs, the proposed scheme implies alternative modes of action for the different networks. According to the reporter gene assay, monomers within the C/S1 network interplay between themselves in a unidirectional fashion, so that S1-bZIPs are sufficient to modify the transcription and have a dominant effect over the C-bZIPs, which are insufficient to alter the gene expression by themselves. Therefore, the mode of action of the C/S1 network can be regarded as a steady exchange of monomers, with the C-bZIPs triggering the dimerization and the S1-bZIPs providing the transactivation capability. Recently, bZIP1 (S1 class) was described to follow a “hit-and-run” mode of action according to which it transiently binds to a large set of cis-elements [77], a model compatible with the dynamic exchange of monomers in the C/S1 network proposed here. Conversely, the function of the G and H networks dimers is broadly influenced by the cellular context, particularly via interactions with other regulatory factors [65, 66, 78]. In fact, the G-bZIPs feature a Proline Rich Domain, which is a protein-protein interaction domain and has been found to be required for their activity [11, 66, 79, 80]. Hence, the mode of action of these dimers would imply the establishment of long lasting dimers that stably bind to their *cis*-element targets, while their transactivation activity can be regulated by other factors. Indeed, the speckle formation observed in these bZIPs reinforces this idea, as they involve the interplay of various components and have been described to be relatively stable structures, independently whether their function in protein degradation or in active transcription processes [70, 81].

## Supporting Information

S1 Fig Scheme of matrix division into the groups to be compared. Elements of the table with the common color belong to the same group. Blue are homotypic combinations of S1-bZIPs, red are homotypic combinations of C-bZIPs, purple are heterodimers between C- and S1-bZIPs, yellow are homotypic combinations of H-bZIPs, green are homotypic combinations of G-bZIPs, light grey are the rest of combinations. Notice that bZIP63-SPN values were not used, they are indicated in dark grey.
(TIF)

S2 Fig **A**) amino acid composition of the buried hydrophobic positions **a** and **d**. Color code: green background indicates optimal positioning of the hydrophobic residues, whereby a distinction is made between the β-branched isoleucine and valine in **a** positions (light green) and the Leucine in **d** positions (dark green). Congruently, the green letters indicate suboptimal, but still hydrophobic residues, in the **d** position Met, Ile, and Val (in decreasing order of stability), and in the **a** positions Met and Leu. Alanine induces a significant decrease in the stability in comparison to the optimal amino acids either in the **a** or in the **d** positions, but it is blue labeled as it is used as a base for determining the relative variations in the stability of the other amino acids. Red letters in these positions indicate destabilizing residues [7, 13, 14, 51, 52, 54]. Besides, background in red and blue are negatively and positively charged residues respectively, yellow is cysteine or Proline, and orange is asparagine in **a** position. **B**) Amino acids in the exposed **b**, **c**, and **f** positions. In green background are residues with a positive hydrophaty index Ile, Val, Leu, Phe, Cys, Met, and Ala [83]. Red, blue, and yellow backgrounds indicate same kind of residues as above. **C**) COILS outputs for the whole amino acid sequences of thee bZIPs indicating the probability of each residue to for a coiled coil. Green, blue, and red lines indicate a window prediction of 14, 21, or 28 residues respectively. The transparent blue bar at the bottom represents the region corresponding to the theoretical LZ starting from the heptad 0 and spanning for 10 heptads in the C- and S1-bZIPs, 7 heptads in the G-bZIPs, and 5 heptads in the H-bZIPs. **D**) PONDR predictions of disorder for the full length bZIPs using the VL-XT algorithm. Values range between 1 (disorder) and 0 (order) and a threshold represented by the horizontal black line is set to 0.5.
(PDF)

S3 Fig Transcriptional effect of the bZIPs on the four selected promoters. **A)** *ASN1*, **B)** *CAT2*, **C)** *RBCS1a*, and **D)** *PDH*. The bars indicate the adjusted mean with the standard error. Red bars are significant increases in the measured GUS activity, green bars are significant decreases, and grey bars are non-significant changes. The blue bars are the control values. All values represent the means of at least 9 biological replicates.
(PDF)

S4 Fig Transcript levels of the bZIP genes estimated from the Genevestigator databases. In all cases the expression levels are represented in Log2 scale. **A**) Scatterplot with the expression across different tissues according to the qPCR experiments. **B)** Levels across the different developmental stages. **C**) Expression of the bZIPs across tissues as in A) but here in boxplot format in order to reflect the high variability in the measurements.
(PDF)

S5 Fig Percent identity matrix for the potential trigger heptads. The fifth, sixth, and seventh heptads of the C- and S1-bZIPs were compared between themselves, revealing that, although these three heptads shared similar properties in regard to the dimer stabilization potential, their amino acid sequences are distant between the two bZIP classes.
(TIF)

S1 File Localization of GFP fusion proteins. Compressed folder (ZIP) with the original confocal images.
(ZIP)

S1 Table Primer list. Excel file with the list of all primers used in this study.
(XLSX)

S2 Table BIFC data. Excel file containing the BiFC assay data, including the raw counts of the BiFC assay, the adjusted BiFC scores, and the comparisons between the reciprocal comparisons.
(XLSX)

S3 Table Comparison of the groups BIFC scores. The groups were tested using the Estimate statement of Proc Glimmix. The resulting p-values were adjusted using the Scheffé method [82].
(PDF)

S4 Table Transactivation assays data. Excel file containing the GUS assay data, including the raw GUS and LUC measurements, the adjusted GUS/LUC means, and the results of the Dunnett’s test.
(XLSX)

## Abbreviations

ASN1: *ASPARAGINE SYNTHETASE 1*
BiFC: Bimolecular fluorescent complementation
BR: Basic region of the bZIP domain
bZIP: Basic region/leucine zipper protein
CAT2: *CATALASE 2*
LZ: Leucine zipper
NLS: Nuclear localization signal
PDH: PROLINE DEHYDROGENASE
RBCS1a: *RUBISCO Small Subunit 1*
SPC: Fusion protein carrying the C-terminal part of the YFP
SPN: Fusion protein carrying the N-terminal part of the YFP
YFP: Yellow fluorescent protein
GFP: Green fluorescent protein
