# Supplementary figures and images for: The Elucidation of the Interactome of 16 Arabidopsis bZIP Factors Reveals Three Independent Functional Networks
Source: PLoS One. 2015 Oct 9;10(10):e0139884. doi: 10.1371/journal.pone.0139884 (PMC4599898; doi:10.1371/journal.pone.0139884)

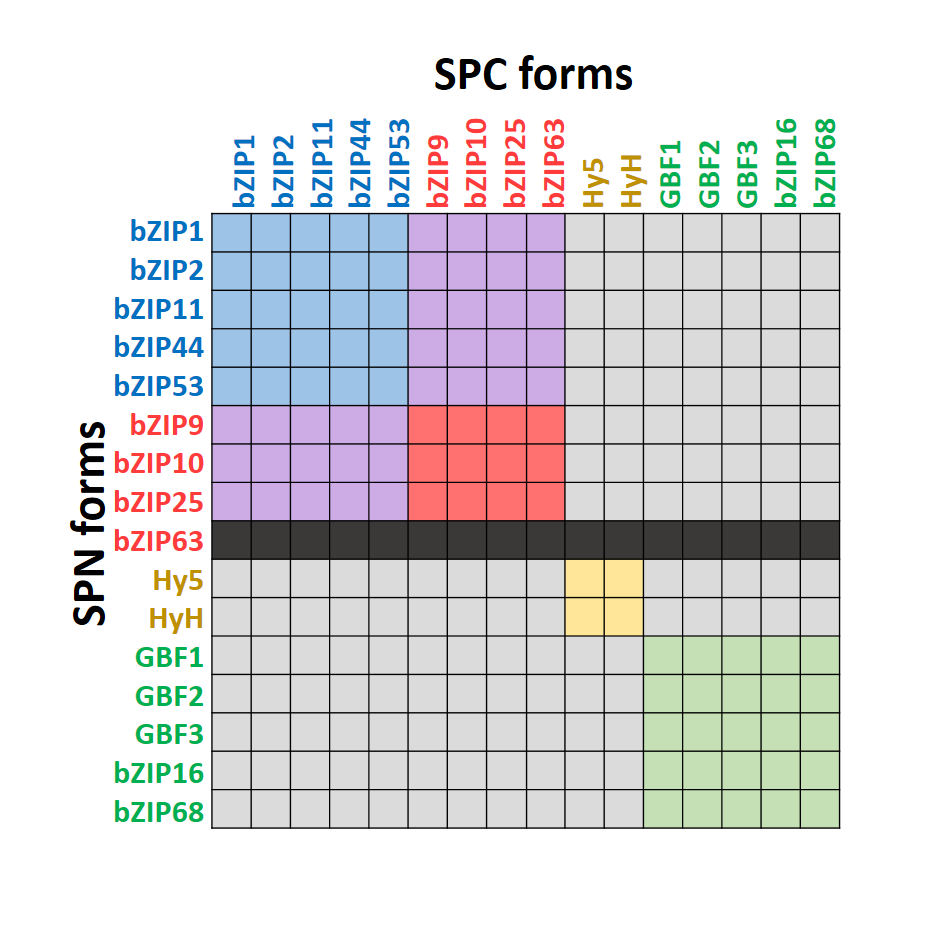

Supplement: S1 Fig — Elements of the table with the common color belong to the same group. Blue are homotypic combinations of S1-bZIPs, red are homotypic combinations of C-bZIPs, purple are heterodimers between C- and S1-bZIPs, yellow are homotypic combinations of H-bZIPs, green are homotypic combinations of G-bZIPs, light grey are the rest of combinations. Notice that bZIP63-SPN values were not used, they are indicated in dark grey. (TIF) [file pone.0139884.s001.tif]

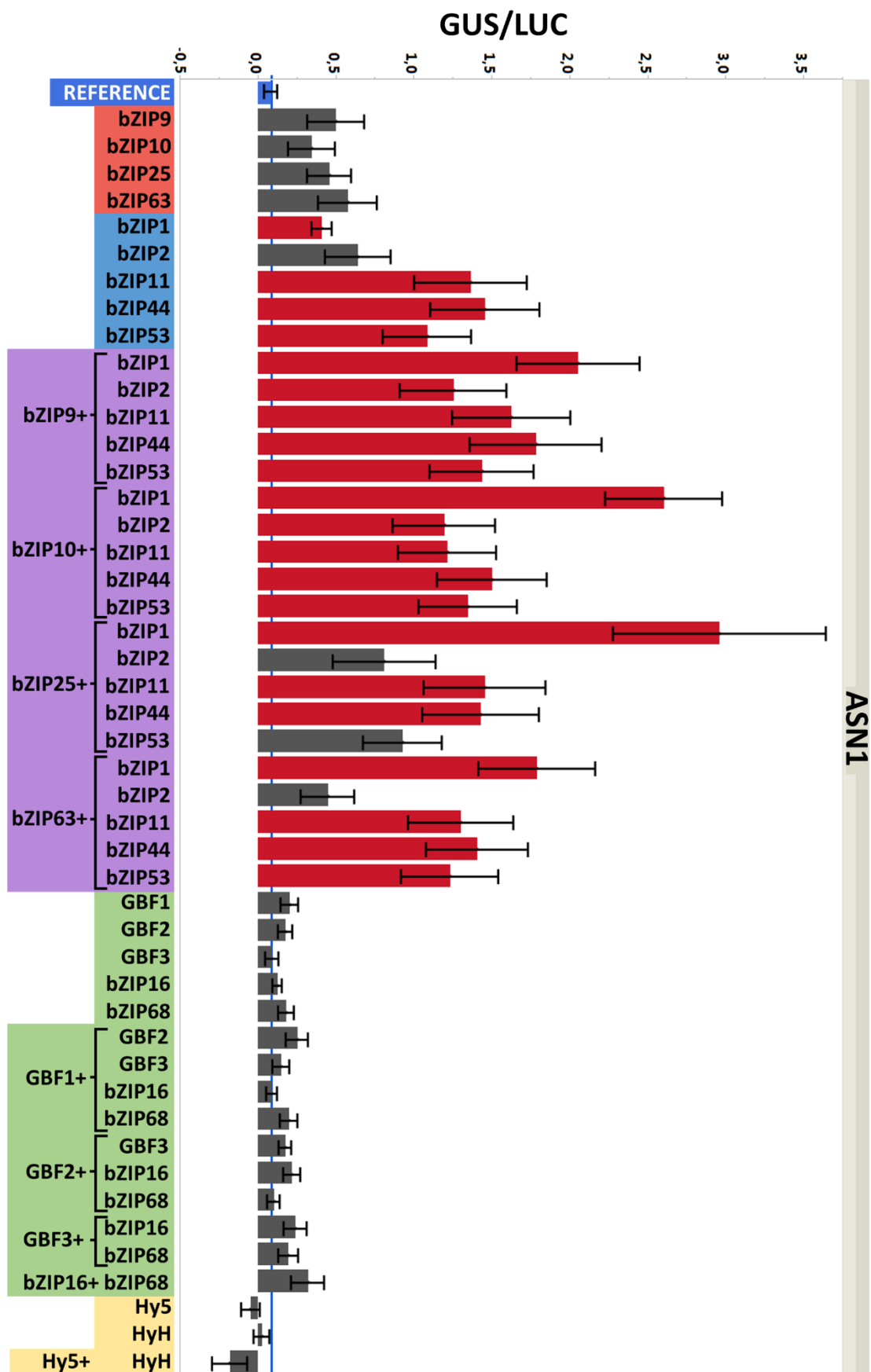

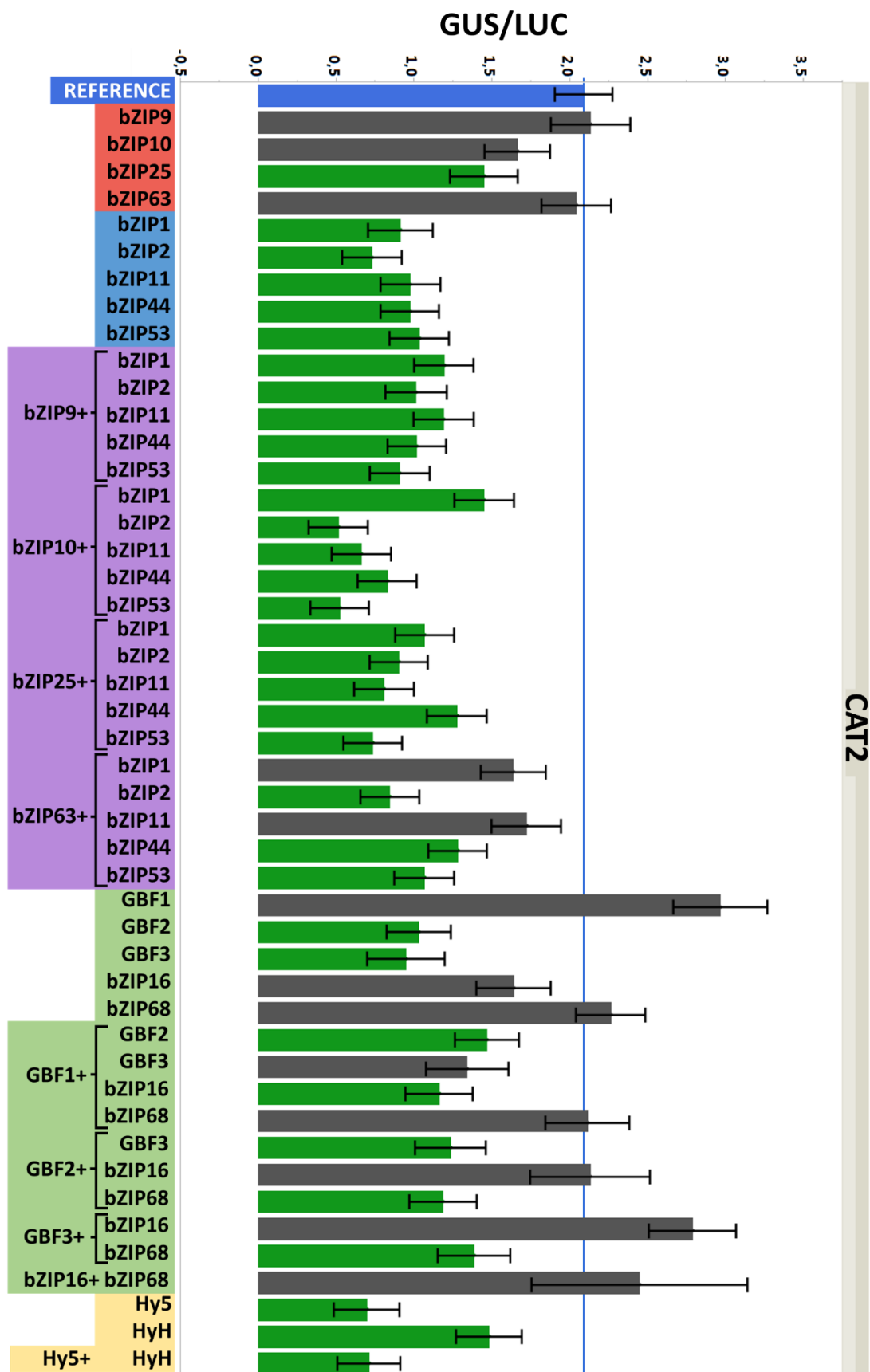

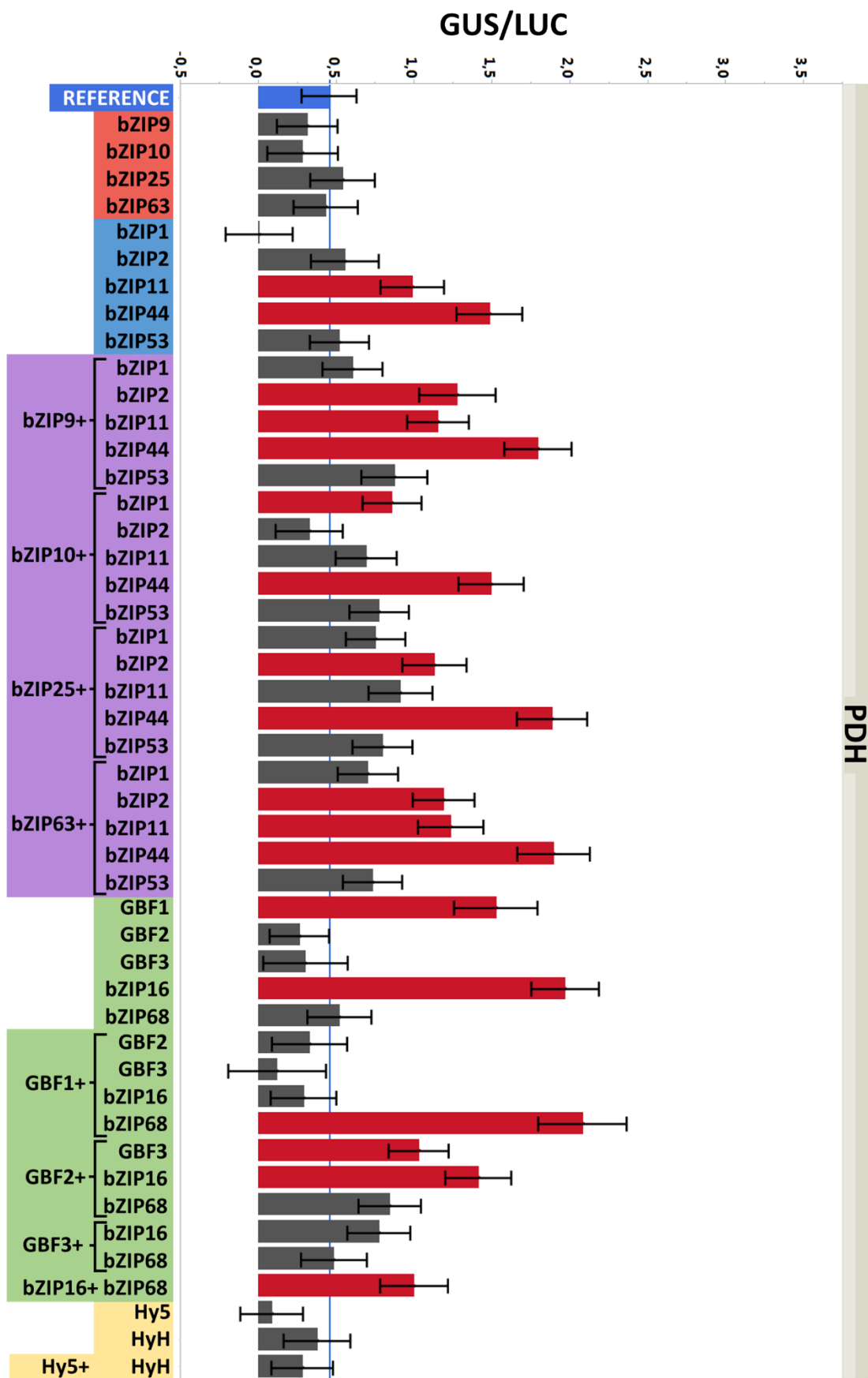

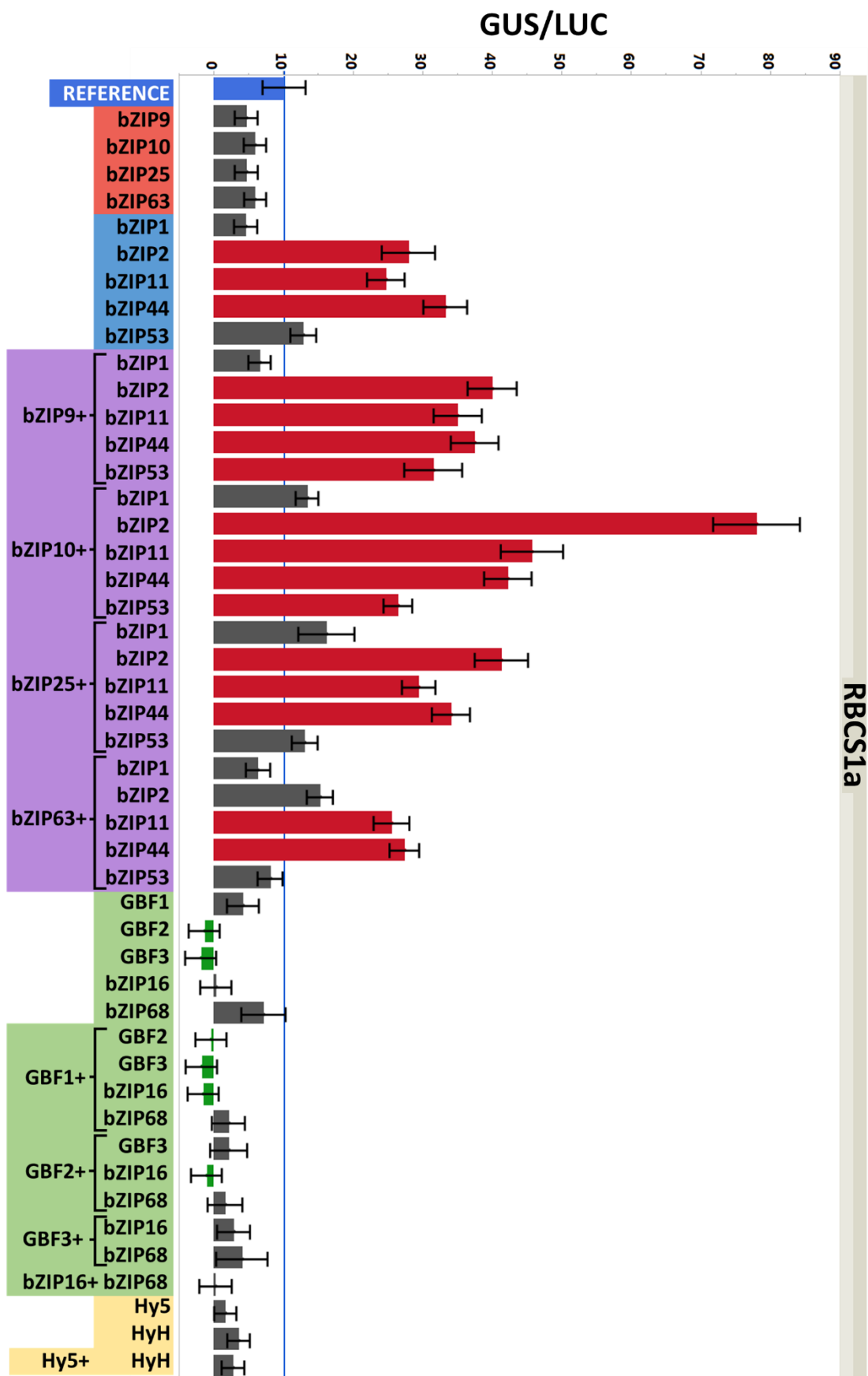

Supplement: S3 Fig — A) ASN1, B) CAT2, C) RBCS1a, and D) PDH. The bars indicate the adjusted mean with the standard error. Red bars are significant increases in the measured GUS activity, green bars are significant decreases, and grey bars are non-significant changes. The blue bars are the control values. All values represent the means of at least 9 biological replicates. (PDF) [file pone.0139884.s003.pdf]

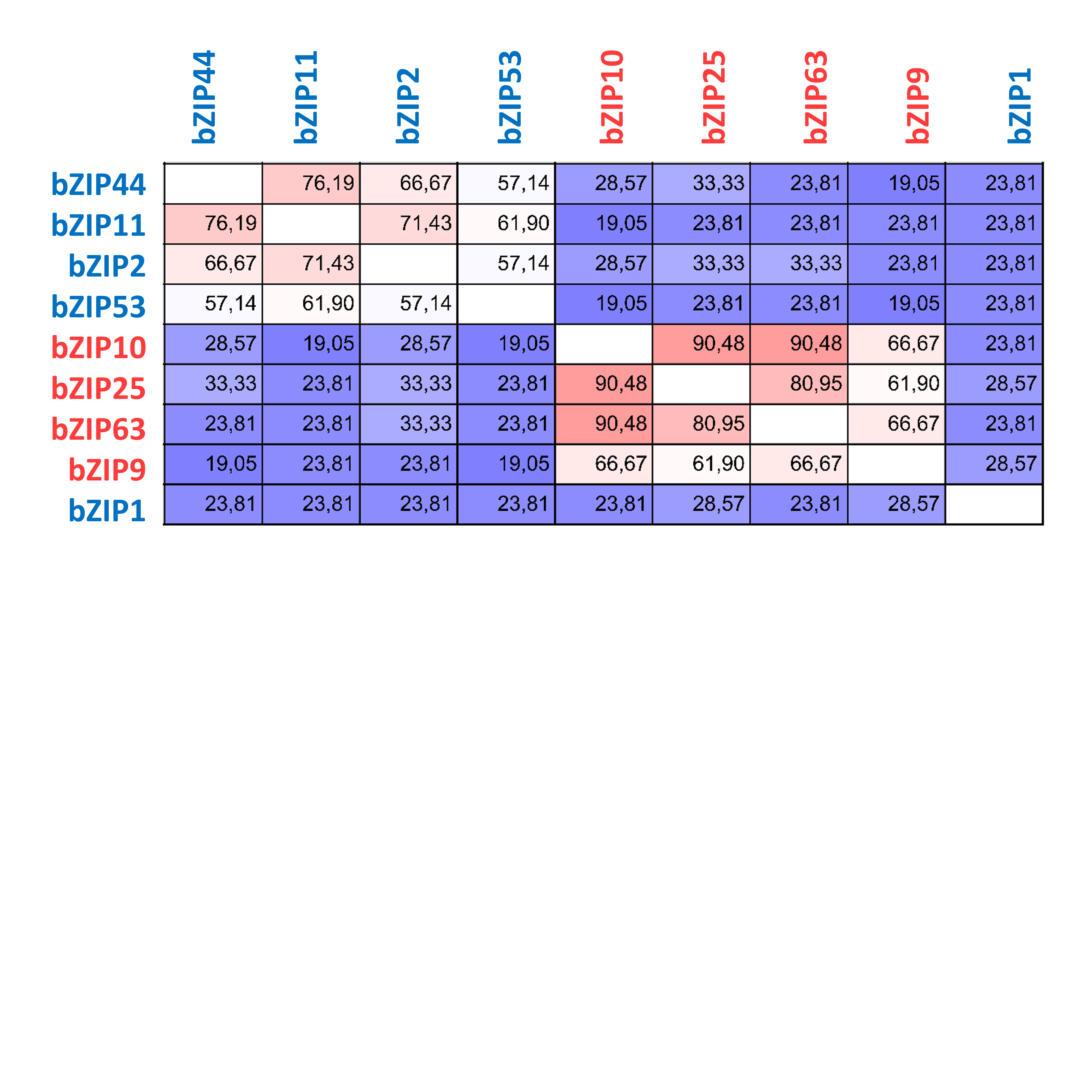

Supplement: S5 Fig — The fifth, sixth, and seventh heptads of the C- and S1-bZIPs were compared between themselves, revealing that, although these three heptads shared similar properties in regard to the dimer stabilization potential, their amino acid sequences are distant between the two bZIP classes. (TIF) [file pone.0139884.s005.tif]

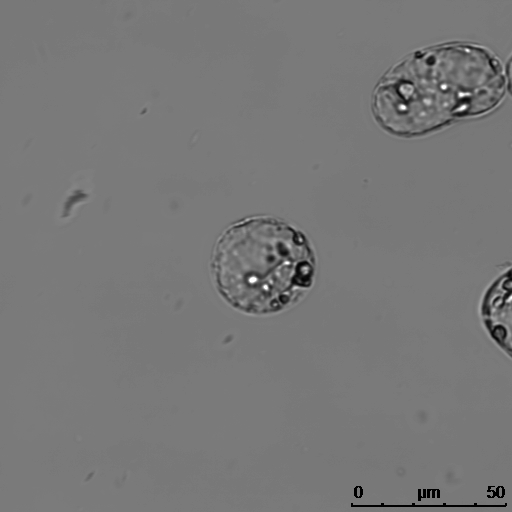

Supplement: S1 File — Compressed folder (ZIP) with the original confocal images. (ZIP) [file pone.0139884.s006.zip › GFP bZIPs/bZIP1 (1).jpg]

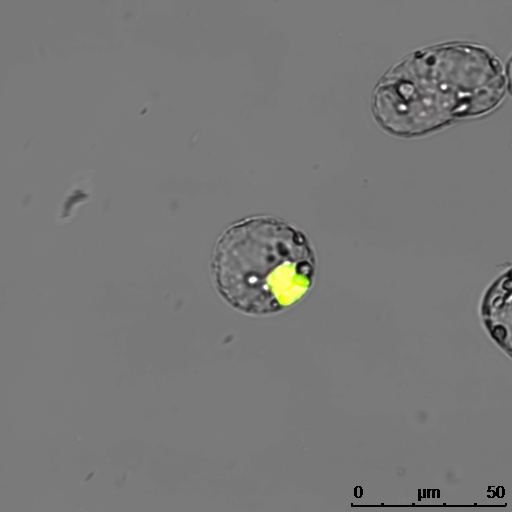

Supplement: S1 File — Compressed folder (ZIP) with the original confocal images. (ZIP) [file pone.0139884.s006.zip › GFP bZIPs/bZIP1 (2).jpg]

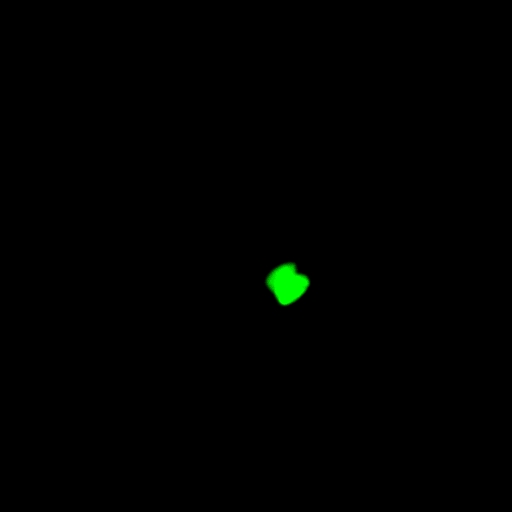

Supplement: S1 File — Compressed folder (ZIP) with the original confocal images. (ZIP) [file pone.0139884.s006.zip › GFP bZIPs/bZIP1 (3).jpg]

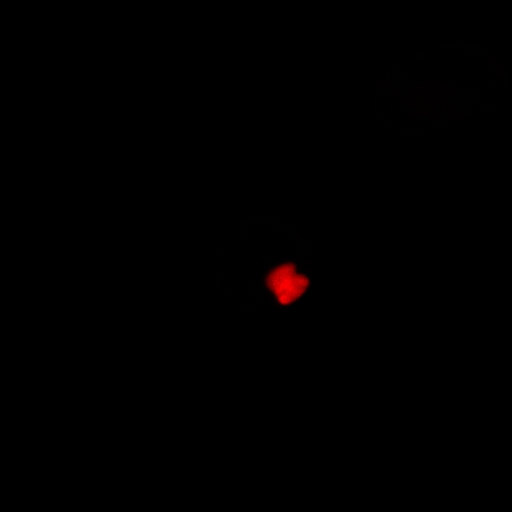

Supplement: S1 File — Compressed folder (ZIP) with the original confocal images. (ZIP) [file pone.0139884.s006.zip › GFP bZIPs/bZIP1 (4).jpg]

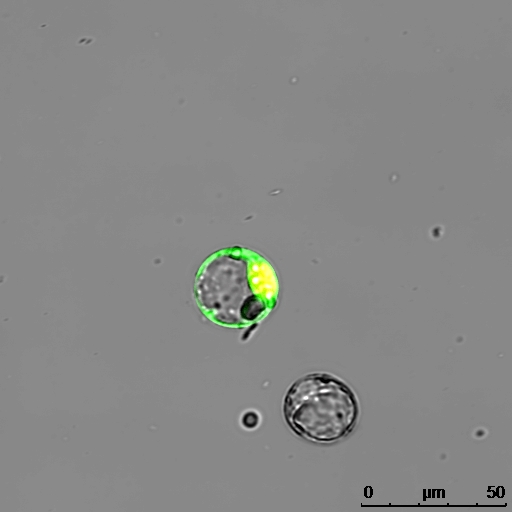

Supplement: S1 File — Compressed folder (ZIP) with the original confocal images. (ZIP) [file pone.0139884.s006.zip › GFP bZIPs/bZIP10 (1).jpg]

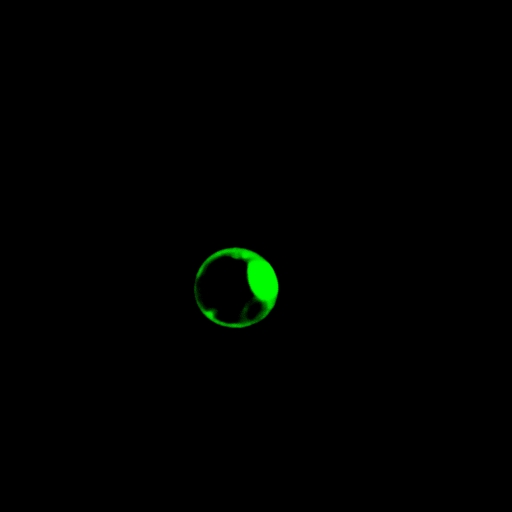

Supplement: S1 File — Compressed folder (ZIP) with the original confocal images. (ZIP) [file pone.0139884.s006.zip › GFP bZIPs/bZIP10 (2).jpg]

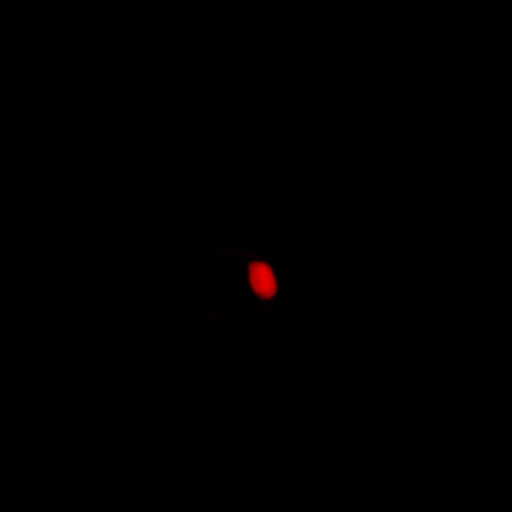

Supplement: S1 File — Compressed folder (ZIP) with the original confocal images. (ZIP) [file pone.0139884.s006.zip › GFP bZIPs/bZIP10 (3).jpg]

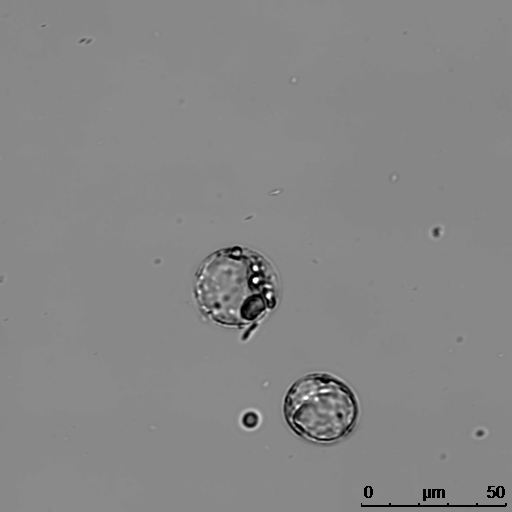

Supplement: S1 File — Compressed folder (ZIP) with the original confocal images. (ZIP) [file pone.0139884.s006.zip › GFP bZIPs/bZIP10 (4).jpg]

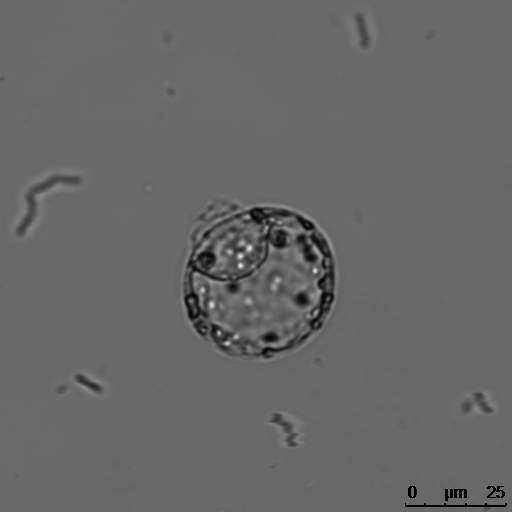

Supplement: S1 File — Compressed folder (ZIP) with the original confocal images. (ZIP) [file pone.0139884.s006.zip › GFP bZIPs/bZIP11 (1).jpg]

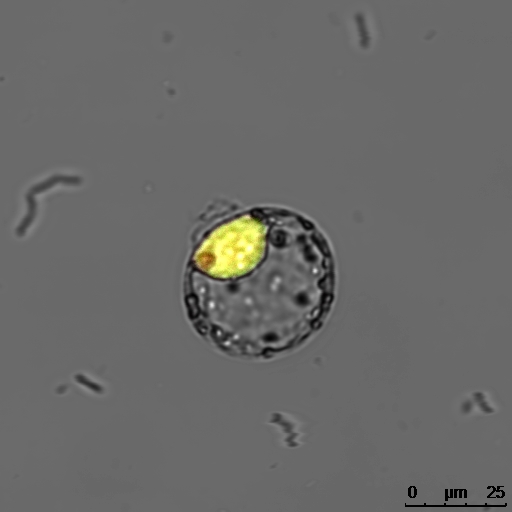

Supplement: S1 File — Compressed folder (ZIP) with the original confocal images. (ZIP) [file pone.0139884.s006.zip › GFP bZIPs/bZIP11 (2).jpg]

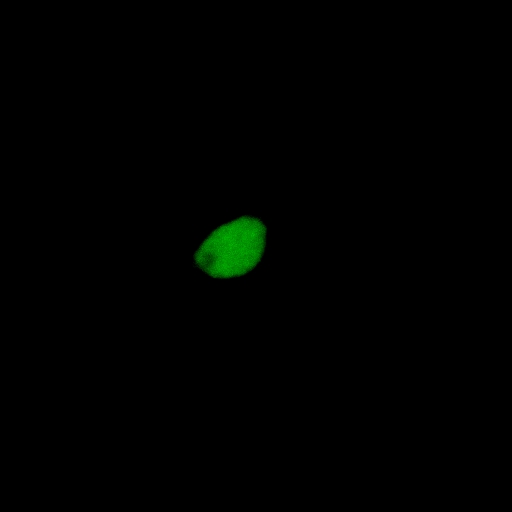

Supplement: S1 File — Compressed folder (ZIP) with the original confocal images. (ZIP) [file pone.0139884.s006.zip › GFP bZIPs/bZIP11 (3).jpg]

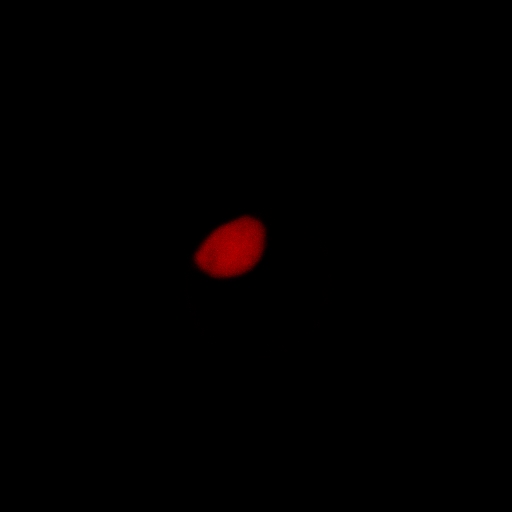

Supplement: S1 File — Compressed folder (ZIP) with the original confocal images. (ZIP) [file pone.0139884.s006.zip › GFP bZIPs/bZIP11 (4).jpg]

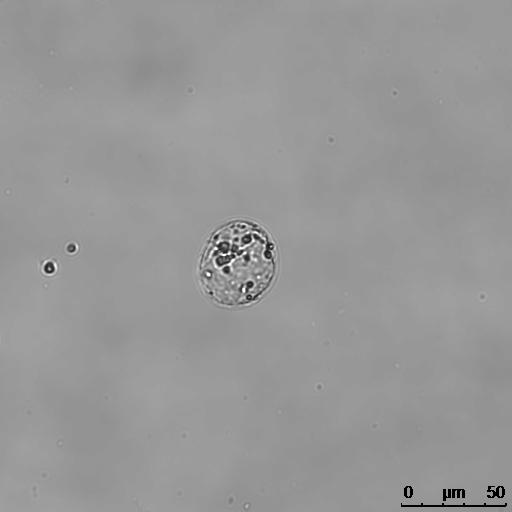

Supplement: S1 File — Compressed folder (ZIP) with the original confocal images. (ZIP) [file pone.0139884.s006.zip › GFP bZIPs/bZIP16 (1).jpg]

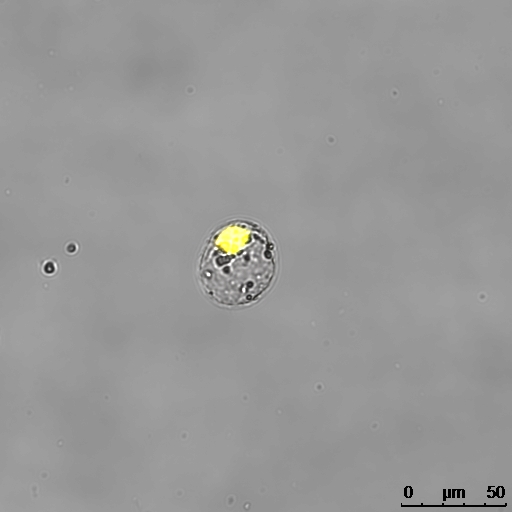

Supplement: S1 File — Compressed folder (ZIP) with the original confocal images. (ZIP) [file pone.0139884.s006.zip › GFP bZIPs/bZIP16 (2).jpg]

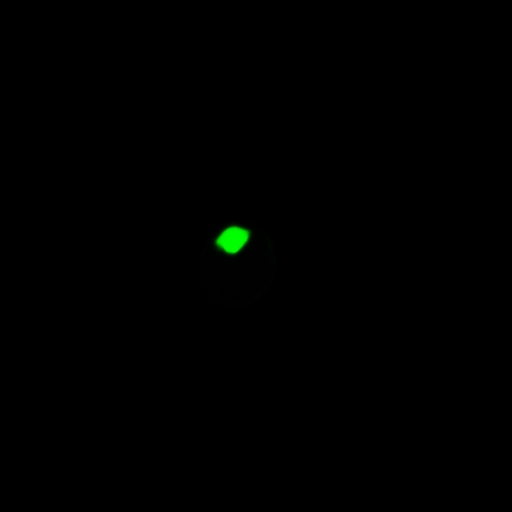

Supplement: S1 File — Compressed folder (ZIP) with the original confocal images. (ZIP) [file pone.0139884.s006.zip › GFP bZIPs/bZIP16 (3).jpg]

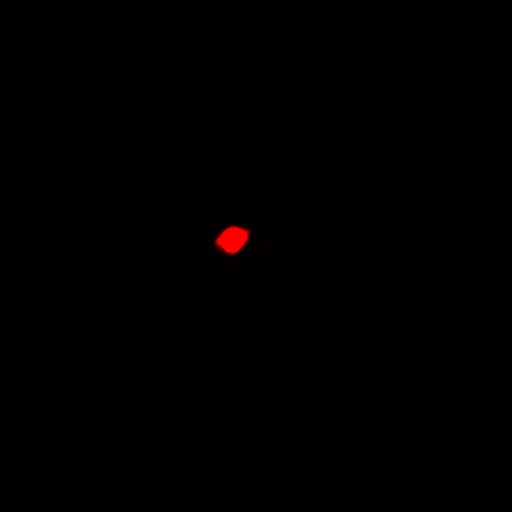

Supplement: S1 File — Compressed folder (ZIP) with the original confocal images. (ZIP) [file pone.0139884.s006.zip › GFP bZIPs/bZIP16 (4).jpg]

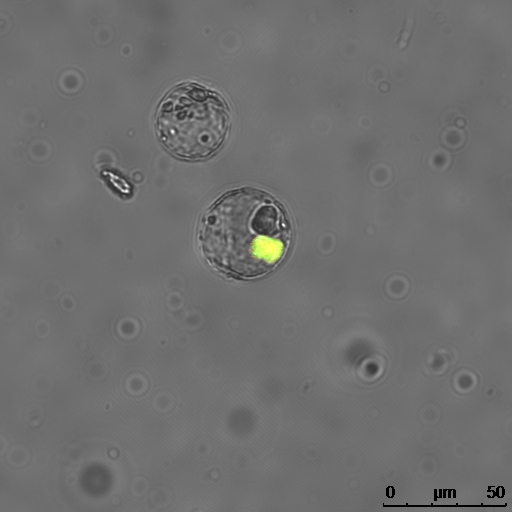

Supplement: S1 File — Compressed folder (ZIP) with the original confocal images. (ZIP) [file pone.0139884.s006.zip › GFP bZIPs/bZIP2 (1).jpg]

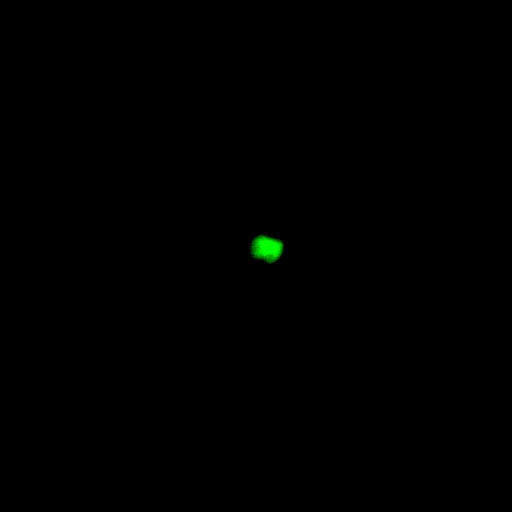

Supplement: S1 File — Compressed folder (ZIP) with the original confocal images. (ZIP) [file pone.0139884.s006.zip › GFP bZIPs/bZIP2 (2).jpg]

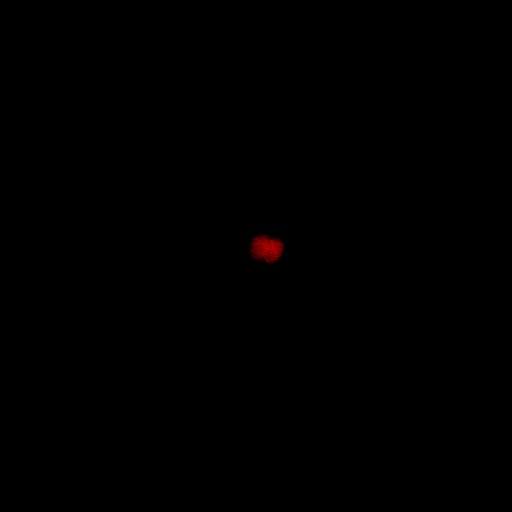

Supplement: S1 File — Compressed folder (ZIP) with the original confocal images. (ZIP) [file pone.0139884.s006.zip › GFP bZIPs/bZIP2 (3).jpg]

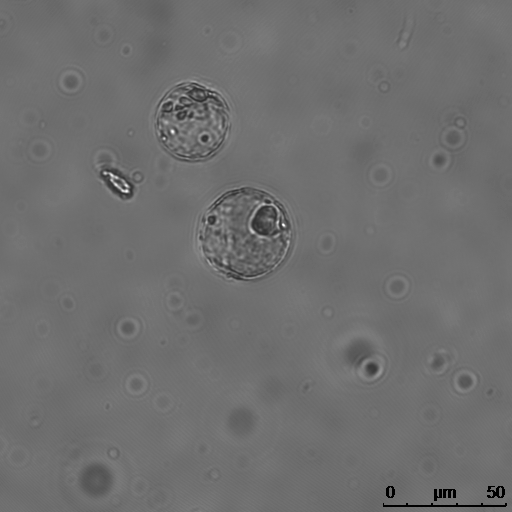

Supplement: S1 File — Compressed folder (ZIP) with the original confocal images. (ZIP) [file pone.0139884.s006.zip › GFP bZIPs/bZIP2 (4).jpg]

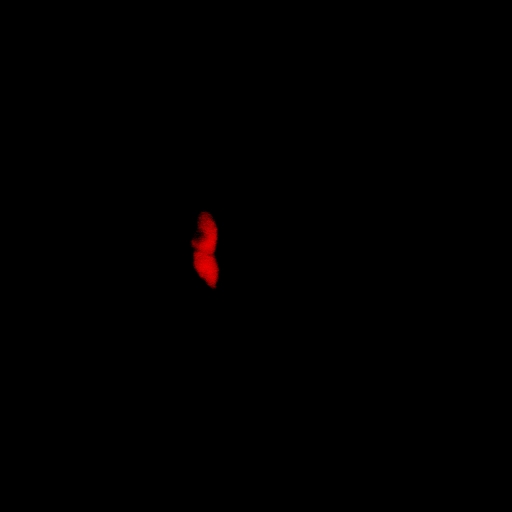

Supplement: S1 File — Compressed folder (ZIP) with the original confocal images. (ZIP) [file pone.0139884.s006.zip › GFP bZIPs/bZIP25 (1).jpg]

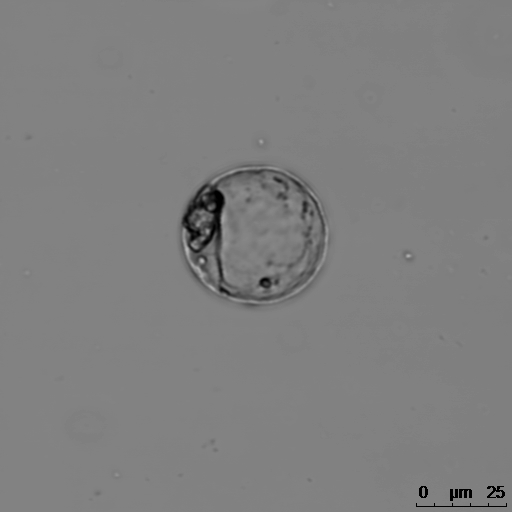

Supplement: S1 File — Compressed folder (ZIP) with the original confocal images. (ZIP) [file pone.0139884.s006.zip › GFP bZIPs/bZIP25 (2).jpg]

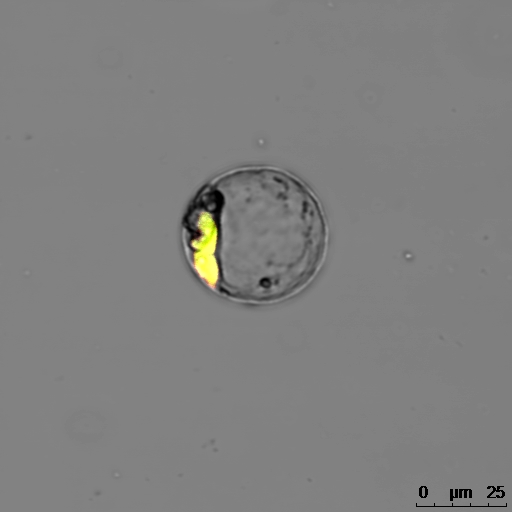

Supplement: S1 File — Compressed folder (ZIP) with the original confocal images. (ZIP) [file pone.0139884.s006.zip › GFP bZIPs/bZIP25 (3).jpg]

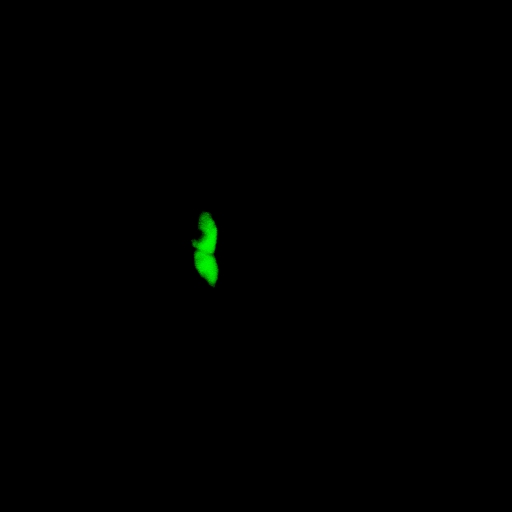

Supplement: S1 File — Compressed folder (ZIP) with the original confocal images. (ZIP) [file pone.0139884.s006.zip › GFP bZIPs/bZIP25 (4).jpg]

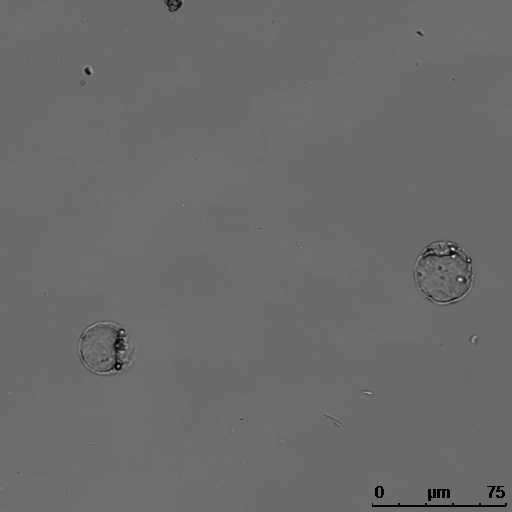

Supplement: S1 File — Compressed folder (ZIP) with the original confocal images. (ZIP) [file pone.0139884.s006.zip › GFP bZIPs/bZIP44 (1).jpg]

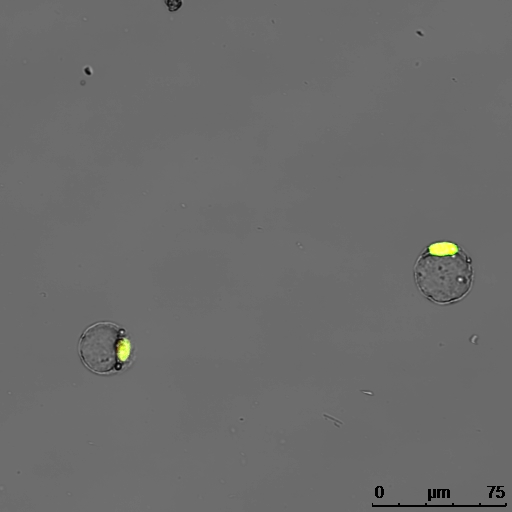

Supplement: S1 File — Compressed folder (ZIP) with the original confocal images. (ZIP) [file pone.0139884.s006.zip › GFP bZIPs/bZIP44 (2).jpg]

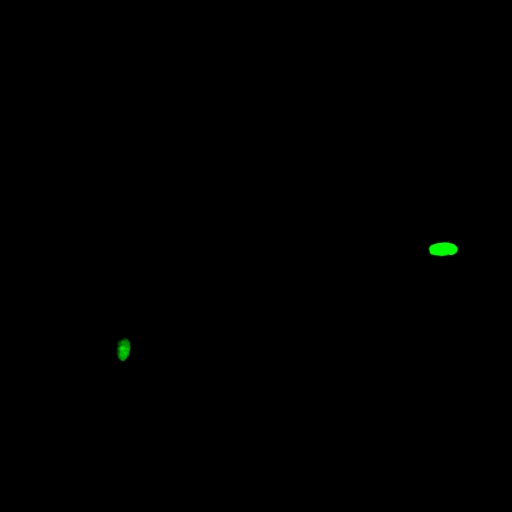

Supplement: S1 File — Compressed folder (ZIP) with the original confocal images. (ZIP) [file pone.0139884.s006.zip › GFP bZIPs/bZIP44 (3).jpg]

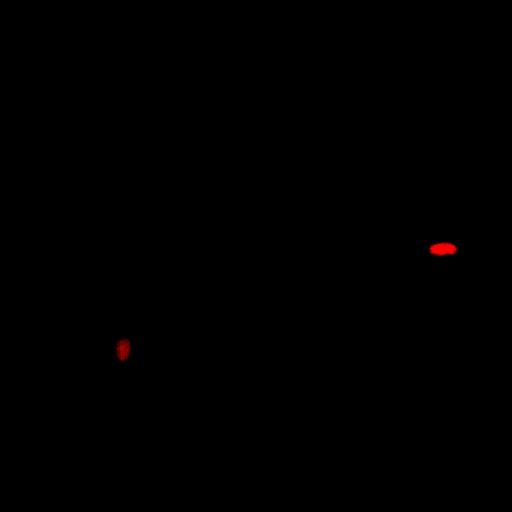

Supplement: S1 File — Compressed folder (ZIP) with the original confocal images. (ZIP) [file pone.0139884.s006.zip › GFP bZIPs/bZIP44 (4).jpg]

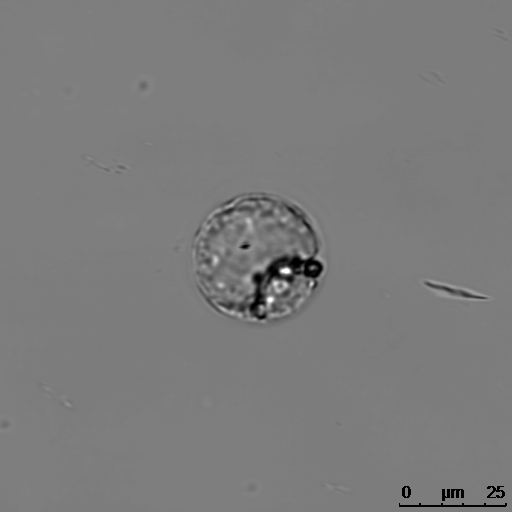

Supplement: S1 File — Compressed folder (ZIP) with the original confocal images. (ZIP) [file pone.0139884.s006.zip › GFP bZIPs/bZIP53 (1).jpg]

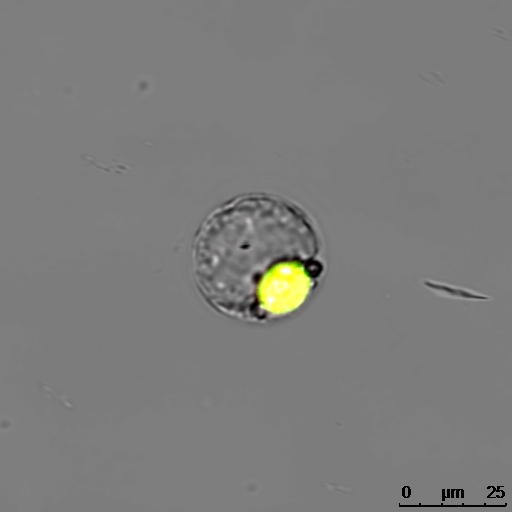

Supplement: S1 File — Compressed folder (ZIP) with the original confocal images. (ZIP) [file pone.0139884.s006.zip › GFP bZIPs/bZIP53 (2).jpg]

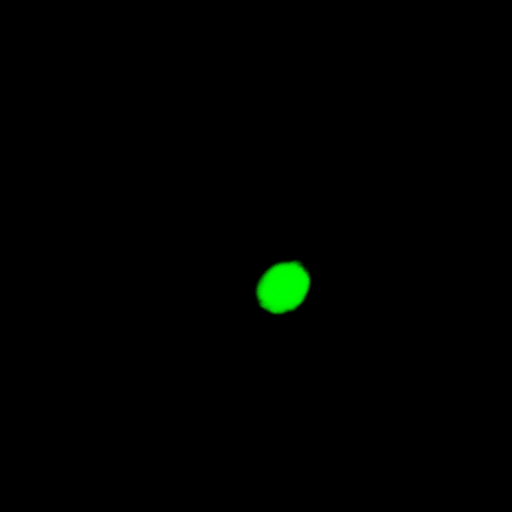

Supplement: S1 File — Compressed folder (ZIP) with the original confocal images. (ZIP) [file pone.0139884.s006.zip › GFP bZIPs/bZIP53 (3).jpg]

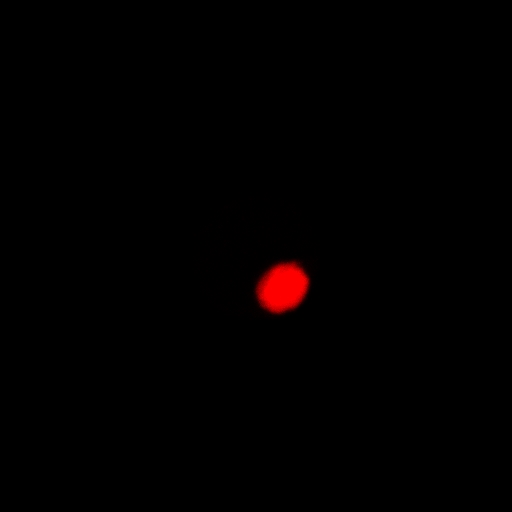

Supplement: S1 File — Compressed folder (ZIP) with the original confocal images. (ZIP) [file pone.0139884.s006.zip › GFP bZIPs/bZIP53 (4).jpg]

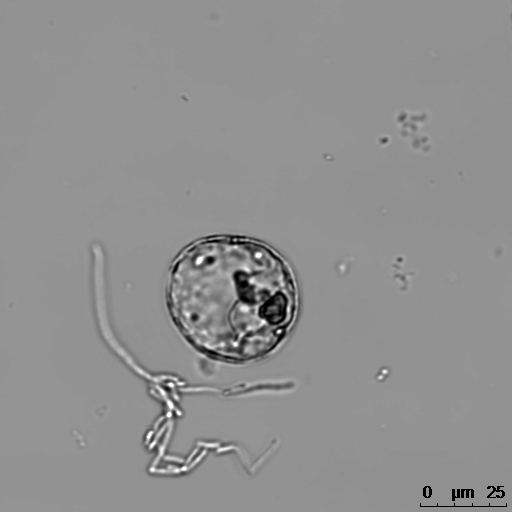

Supplement: S1 File — Compressed folder (ZIP) with the original confocal images. (ZIP) [file pone.0139884.s006.zip › GFP bZIPs/bZIP63 (1).jpg]

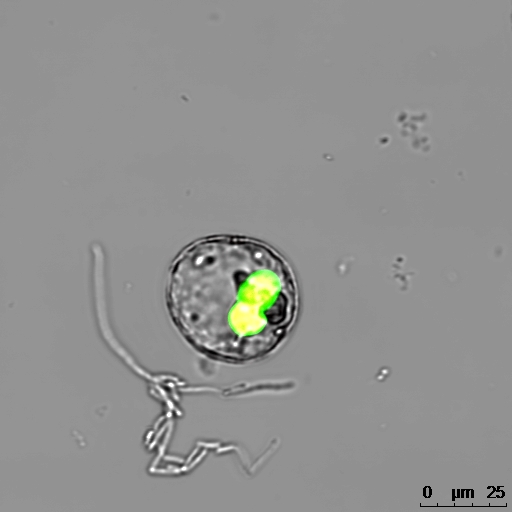

Supplement: S1 File — Compressed folder (ZIP) with the original confocal images. (ZIP) [file pone.0139884.s006.zip › GFP bZIPs/bZIP63 (2).jpg]

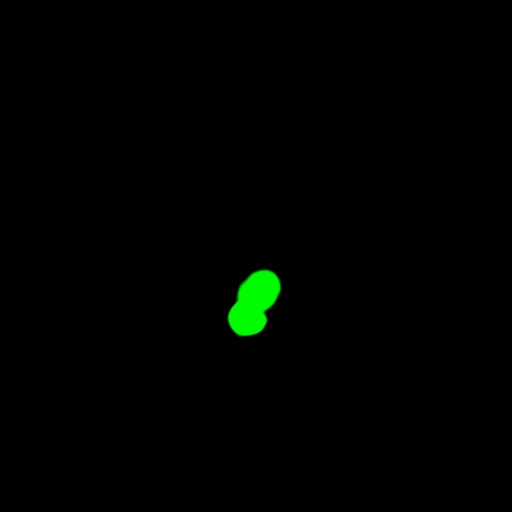

Supplement: S1 File — Compressed folder (ZIP) with the original confocal images. (ZIP) [file pone.0139884.s006.zip › GFP bZIPs/bZIP63 (3).jpg]

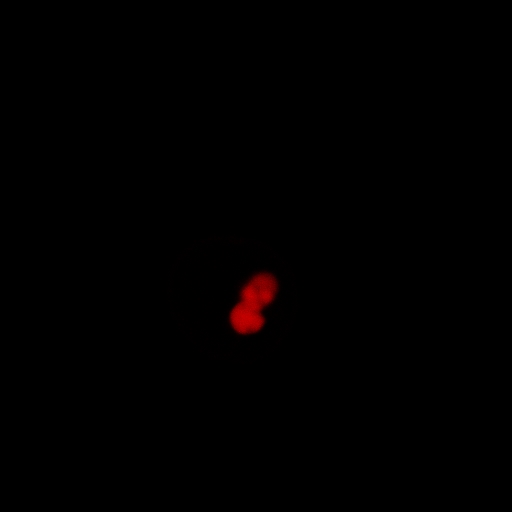

Supplement: S1 File — Compressed folder (ZIP) with the original confocal images. (ZIP) [file pone.0139884.s006.zip › GFP bZIPs/bZIP63 (4).jpg]

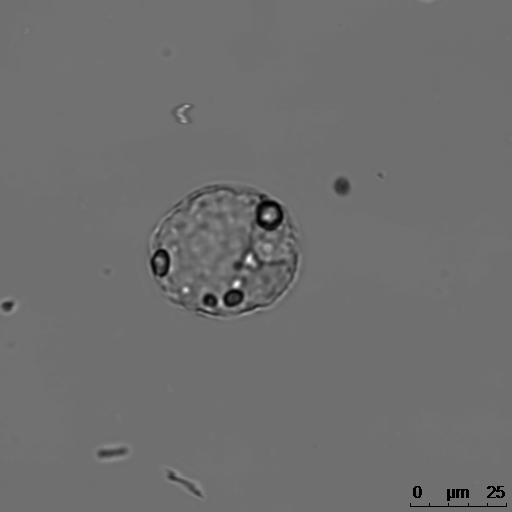

Supplement: S1 File — Compressed folder (ZIP) with the original confocal images. (ZIP) [file pone.0139884.s006.zip › GFP bZIPs/bZIP68 (1).jpg]

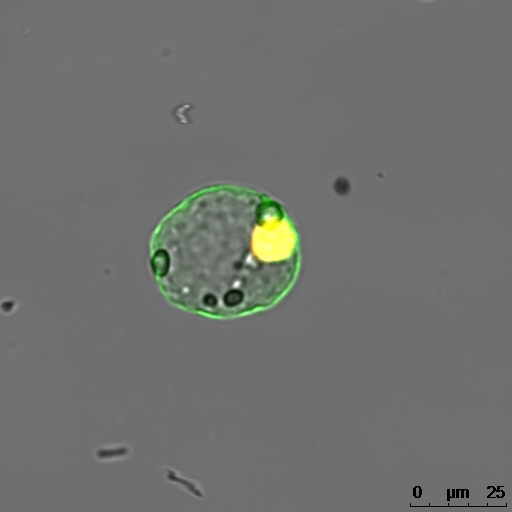

Supplement: S1 File — Compressed folder (ZIP) with the original confocal images. (ZIP) [file pone.0139884.s006.zip › GFP bZIPs/bZIP68 (2).jpg]

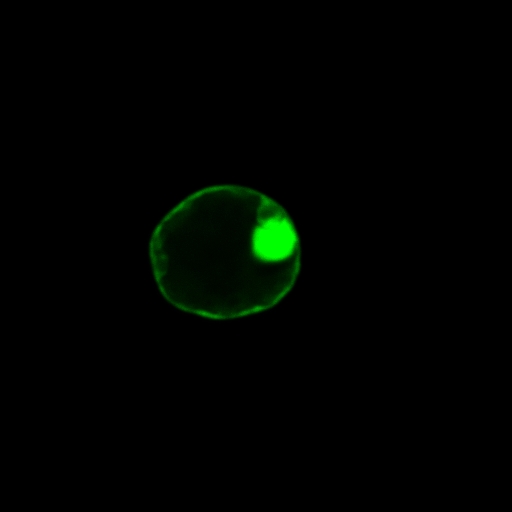

Supplement: S1 File — Compressed folder (ZIP) with the original confocal images. (ZIP) [file pone.0139884.s006.zip › GFP bZIPs/bZIP68 (3).jpg]

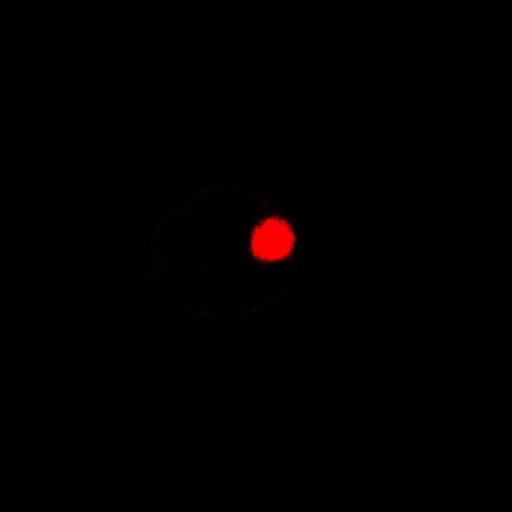

Supplement: S1 File — Compressed folder (ZIP) with the original confocal images. (ZIP) [file pone.0139884.s006.zip › GFP bZIPs/bZIP68 (4).jpg]

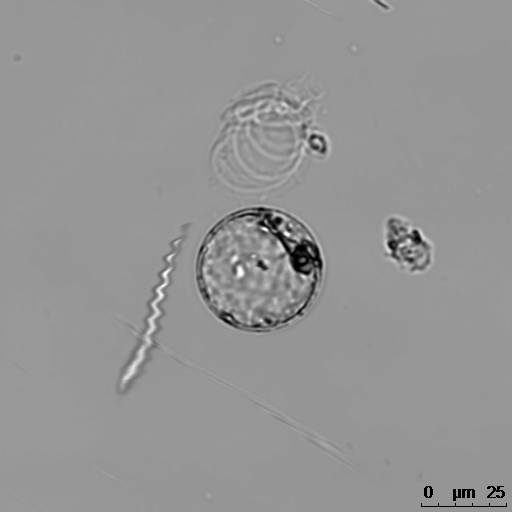

Supplement: S1 File — Compressed folder (ZIP) with the original confocal images. (ZIP) [file pone.0139884.s006.zip › GFP bZIPs/bZIP9 (1).jpg]

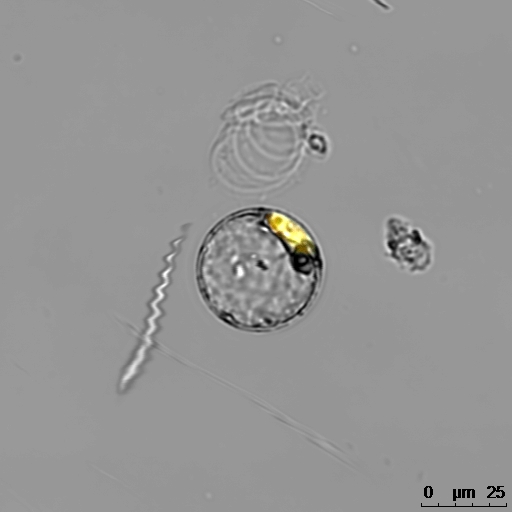

Supplement: S1 File — Compressed folder (ZIP) with the original confocal images. (ZIP) [file pone.0139884.s006.zip › GFP bZIPs/bZIP9 (2).jpg]

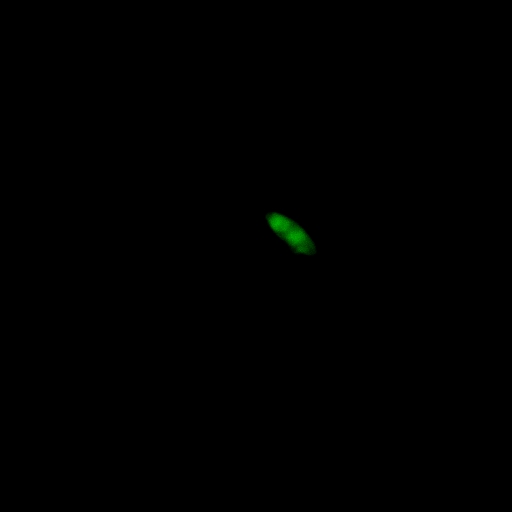

Supplement: S1 File — Compressed folder (ZIP) with the original confocal images. (ZIP) [file pone.0139884.s006.zip › GFP bZIPs/bZIP9 (3).jpg]

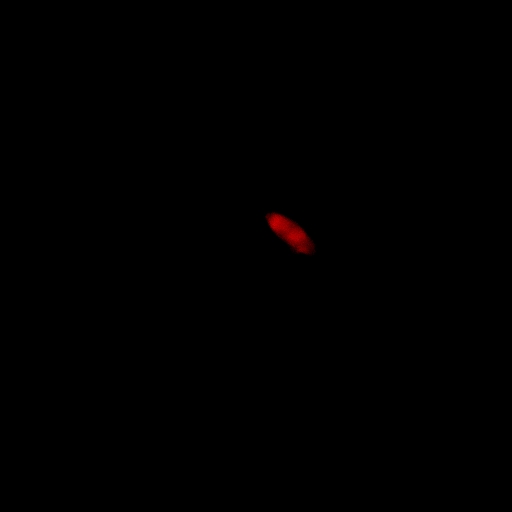

Supplement: S1 File — Compressed folder (ZIP) with the original confocal images. (ZIP) [file pone.0139884.s006.zip › GFP bZIPs/bZIP9 (4).jpg]

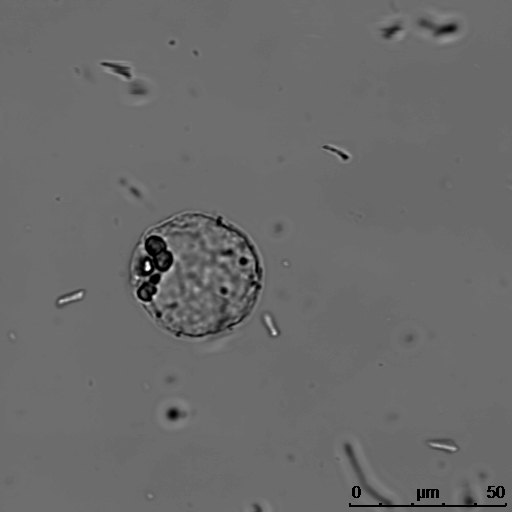

Supplement: S1 File — Compressed folder (ZIP) with the original confocal images. (ZIP) [file pone.0139884.s006.zip › GFP bZIPs/GBF1 (1).jpg]

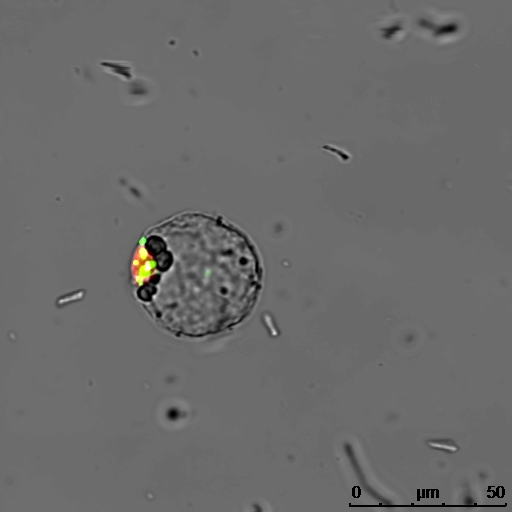

Supplement: S1 File — Compressed folder (ZIP) with the original confocal images. (ZIP) [file pone.0139884.s006.zip › GFP bZIPs/GBF1 (2).jpg]

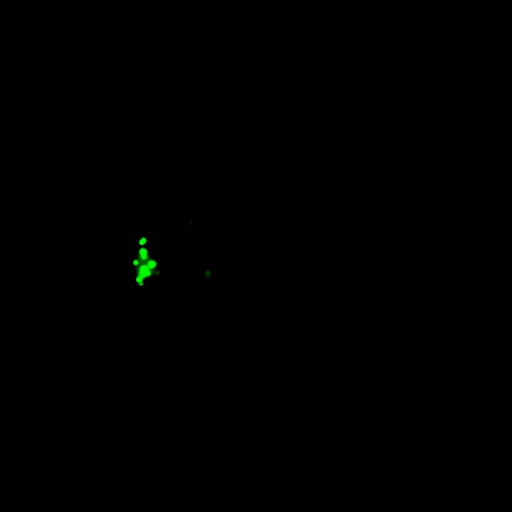

Supplement: S1 File — Compressed folder (ZIP) with the original confocal images. (ZIP) [file pone.0139884.s006.zip › GFP bZIPs/GBF1 (3).jpg]

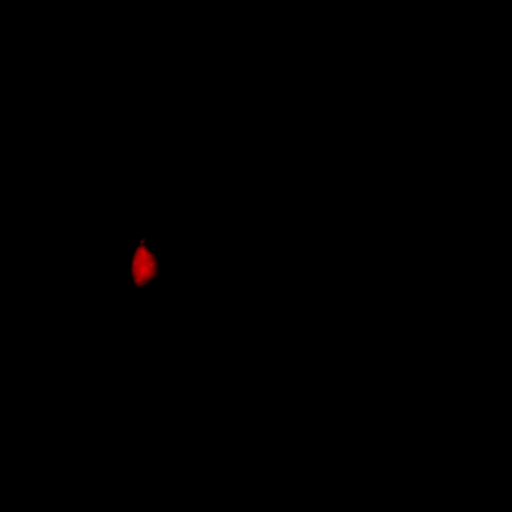

Supplement: S1 File — Compressed folder (ZIP) with the original confocal images. (ZIP) [file pone.0139884.s006.zip › GFP bZIPs/GBF1 (4).jpg]

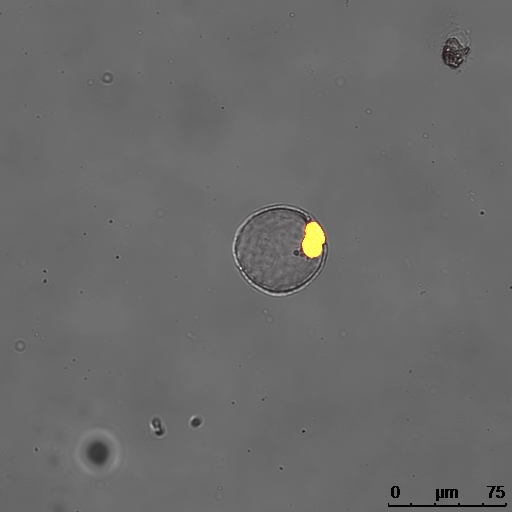

Supplement: S1 File — Compressed folder (ZIP) with the original confocal images. (ZIP) [file pone.0139884.s006.zip › GFP bZIPs/GBF2 (1).jpg]

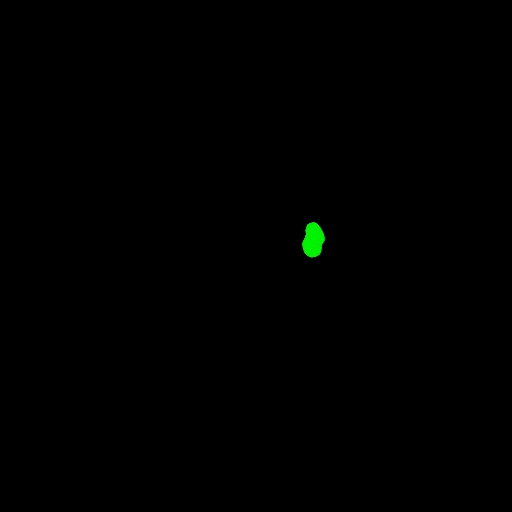

Supplement: S1 File — Compressed folder (ZIP) with the original confocal images. (ZIP) [file pone.0139884.s006.zip › GFP bZIPs/GBF2 (2).jpg]

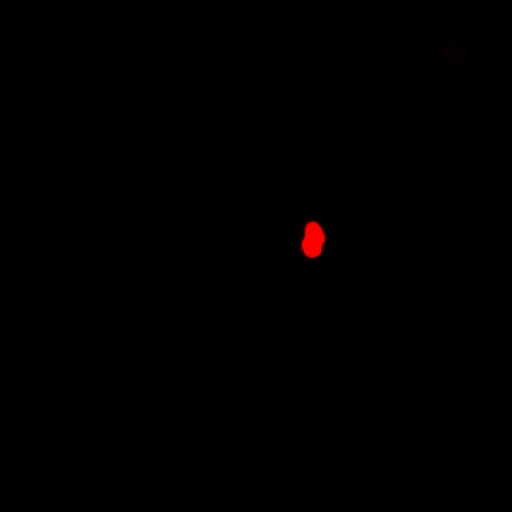

Supplement: S1 File — Compressed folder (ZIP) with the original confocal images. (ZIP) [file pone.0139884.s006.zip › GFP bZIPs/GBF2 (3).jpg]

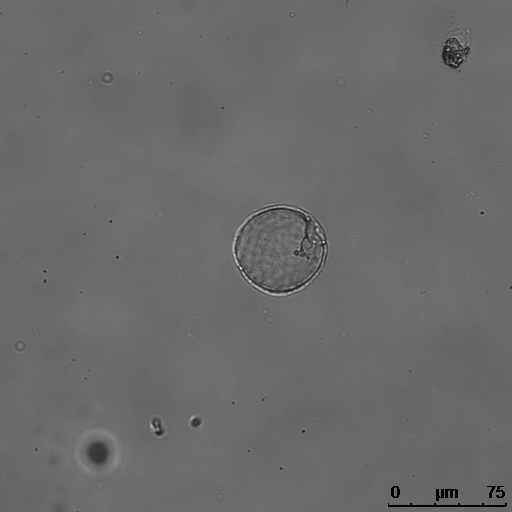

Supplement: S1 File — Compressed folder (ZIP) with the original confocal images. (ZIP) [file pone.0139884.s006.zip › GFP bZIPs/GBF2 (4).jpg]

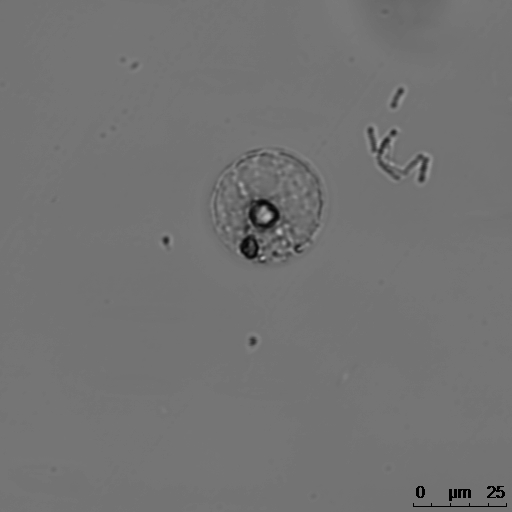

Supplement: S1 File — Compressed folder (ZIP) with the original confocal images. (ZIP) [file pone.0139884.s006.zip › GFP bZIPs/GBF3 (1).jpg]

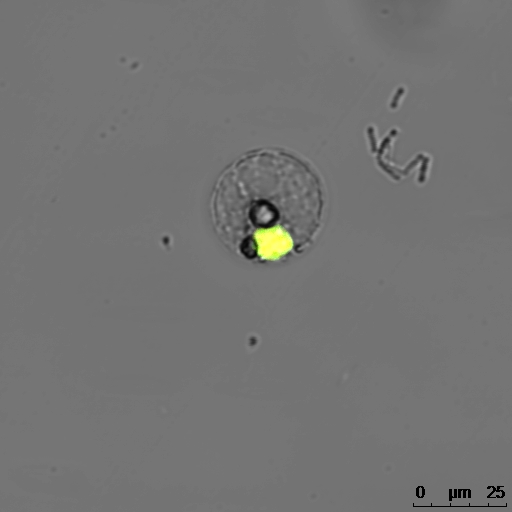

Supplement: S1 File — Compressed folder (ZIP) with the original confocal images. (ZIP) [file pone.0139884.s006.zip › GFP bZIPs/GBF3 (2).jpg]

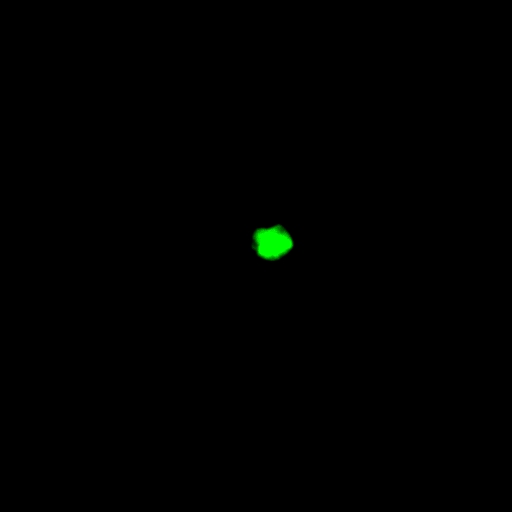

Supplement: S1 File — Compressed folder (ZIP) with the original confocal images. (ZIP) [file pone.0139884.s006.zip › GFP bZIPs/GBF3 (3).jpg]

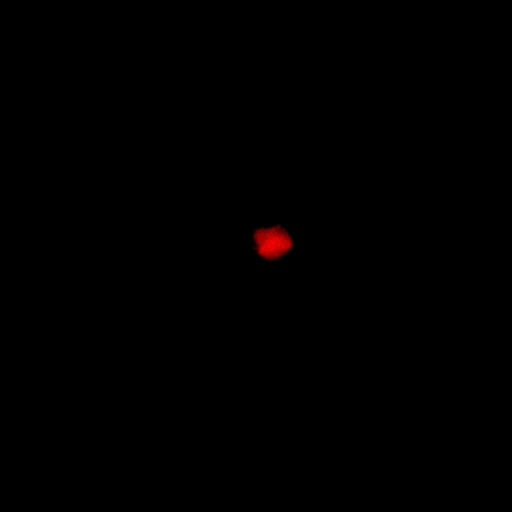

Supplement: S1 File — Compressed folder (ZIP) with the original confocal images. (ZIP) [file pone.0139884.s006.zip › GFP bZIPs/GBF3 (4).jpg]

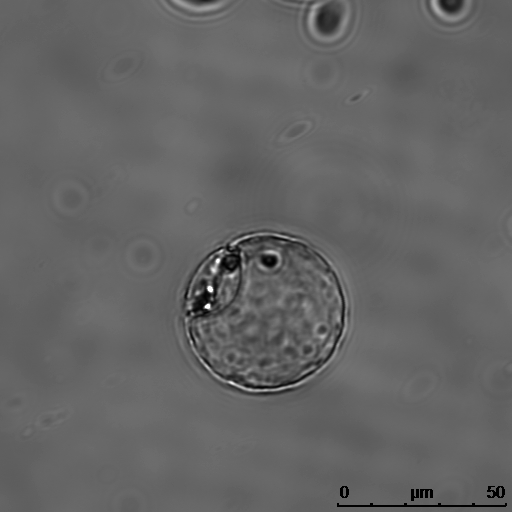

Supplement: S1 File — Compressed folder (ZIP) with the original confocal images. (ZIP) [file pone.0139884.s006.zip › GFP bZIPs/Hy5 (1).jpg]

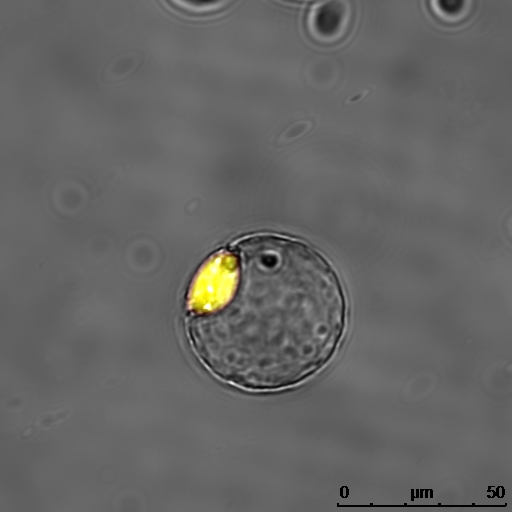

Supplement: S1 File — Compressed folder (ZIP) with the original confocal images. (ZIP) [file pone.0139884.s006.zip › GFP bZIPs/Hy5 (2).jpg]

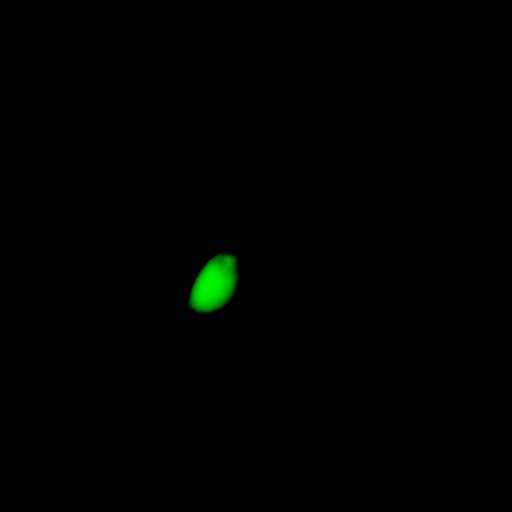

Supplement: S1 File — Compressed folder (ZIP) with the original confocal images. (ZIP) [file pone.0139884.s006.zip › GFP bZIPs/Hy5 (3).jpg]

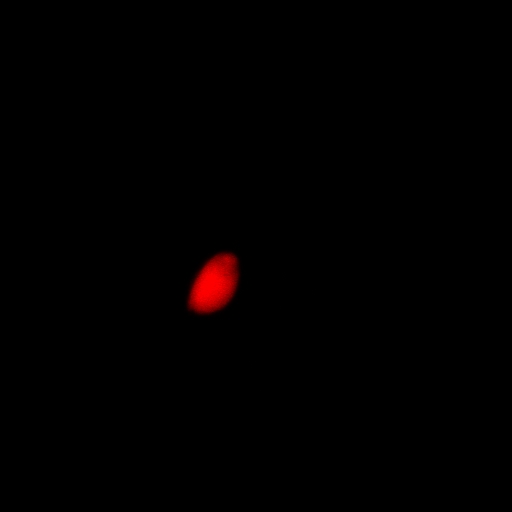

Supplement: S1 File — Compressed folder (ZIP) with the original confocal images. (ZIP) [file pone.0139884.s006.zip › GFP bZIPs/Hy5 (4).jpg]

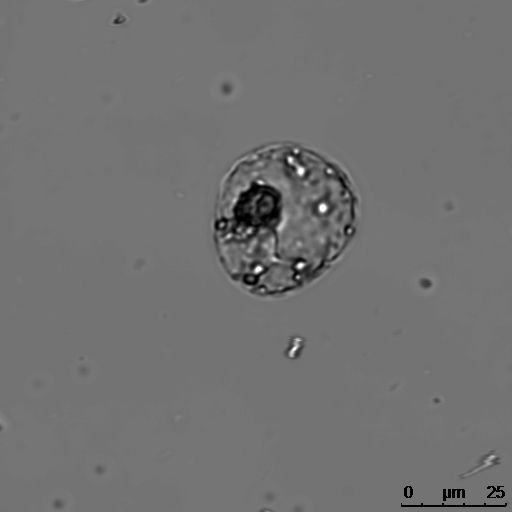

Supplement: S1 File — Compressed folder (ZIP) with the original confocal images. (ZIP) [file pone.0139884.s006.zip › GFP bZIPs/HyH (1).jpg]

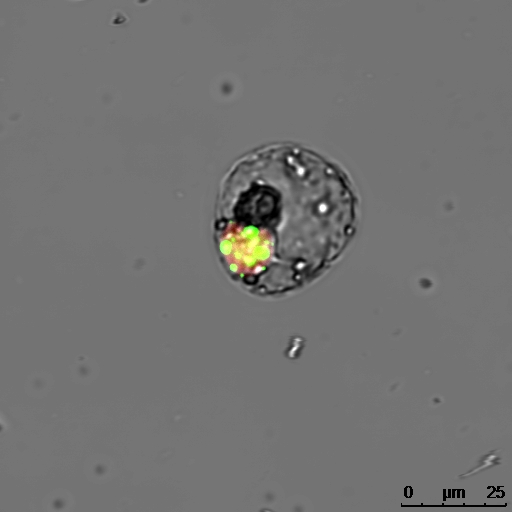

Supplement: S1 File — Compressed folder (ZIP) with the original confocal images. (ZIP) [file pone.0139884.s006.zip › GFP bZIPs/HyH (2).jpg]

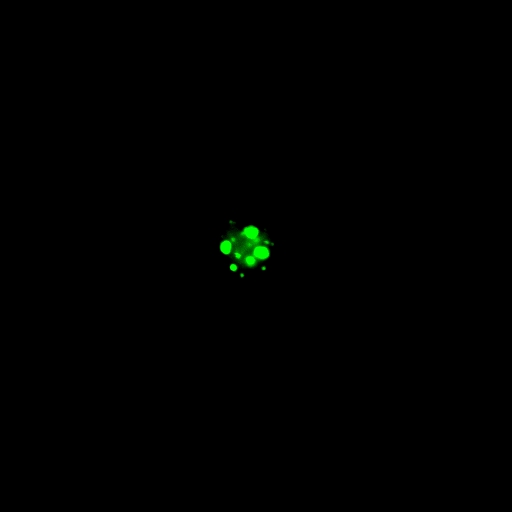

Supplement: S1 File — Compressed folder (ZIP) with the original confocal images. (ZIP) [file pone.0139884.s006.zip › GFP bZIPs/HyH (3).jpg]

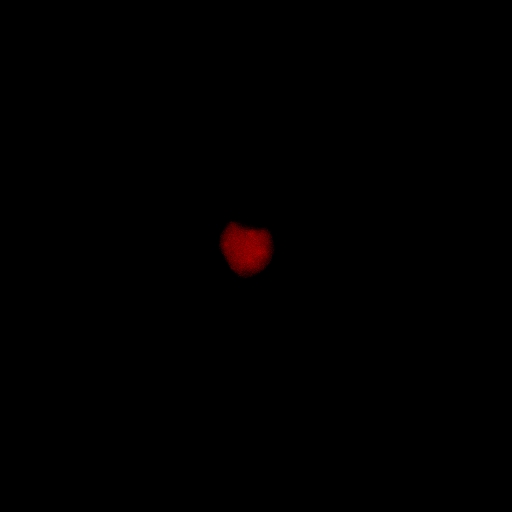

Supplement: S1 File — Compressed folder (ZIP) with the original confocal images. (ZIP) [file pone.0139884.s006.zip › GFP bZIPs/HyH (4).jpg]
