# Supplementary material for: The Elucidation of the Interactome of 16 Arabidopsis bZIP Factors Reveals Three Independent Functional Networks
Source: PLoS One. 2015 Oct 9;10(10):e0139884. doi: 10.1371/journal.pone.0139884 (PMC4599898; doi:10.1371/journal.pone.0139884)
Supplement: S2 Fig — A) amino acid composition of the buried hydrophobic positions a and d. Color code: green background indicates optimal positioning of the hydrophobic residues, whereby a distinction is made between the β-branched isoleucine and valine in a positions (light green) and the Leucine in d positions (dark green). Congruently, the green letters indicate suboptimal, but still hydrophobic residues, in the d position Met, Ile, and Val (in decreasing order of stability), and in the a positions Met and Leu. Alanine induces a significant decrease in the stability in comparison to the optimal amino acids either in the a or in the d positions, but it is blue labeled as it is used as a base for determining the relative variations in the stability of the other amino acids. Red letters in these positions indicate destabilizing residues [7, 13, 14, 51, 52, 54]. Besides, background in red and blue are negatively and positively charged residues respectively, yellow is cysteine or Proline, and orange is asparagine in a position. B) Amino acids in the exposed b, c, and f positions. In green background are residues with a positive hydrophaty index Ile, Val, Leu, Phe, Cys, Met, and Ala [83]. Red, blue, and yellow backgrounds indicate same kind of residues as above. C) COILS outputs for the whole amino acid sequences of thee bZIPs indicating the probability of each residue to for a coiled coil. Green, blue, and red lines indicate a window prediction of 14, 21, or 28 residues respectively. The transparent blue bar at the bottom represents the region corresponding to the theoretical LZ starting from the heptad 0 and spanning for 10 heptads in the C- and S1-bZIPs, 7 heptads in the G-bZIPs, and 5 heptads in the H-bZIPs. D) PONDR predictions of disorder for the full length bZIPs using the VL-XT algorithm. Values range between 1 (disorder) and 0 (order) and a threshold represented by the horizontal black line is set to 0.5. (PDF) [file pone.0139884.s002.pdf]

A)

			0		1		2		3		4		5		6		7		8		9		10		11	
	a	d	a	d	a	d	a	d	a	d	a	d	a	d	a	d	a	d	a	d	a	d	a	d	a	d
**bZIP10**		K	T	L	V	L	H	L	L	M	Y	A	N	L	I	L	V	A	V	V	N	L	S	H	N	P
**bZIP25**		K	M	F	V	L	H	L	L	M	Y	A	N	L	I	L	V	A	V	V	N	H	P	G	S	P
**bZIP63**		K	L	L	V	L	N	L	L	V	F	A	N	L	I	L	V	A	V	L	N	F	P	V	S	S
**bZIP9**		K	L	L	V	L	N	L	L	A	F	A	N	L	V	L	V	A	V	G	S	N	Q	L	S	I
**bZIP44**		K	L	L	V	L	N	I	I	T	Y	I	N	L	V	L	L	L	V	V	S	F	T	G	G	F
**bZIP53**		K	L	L	V	L	N	I	V	A	Y	M	N	L	A	L	L	L	L	V	S	A	P	P	Q	W
**bZIP2**		K	V	L	I	L	N	I	L	T	Y	I	N	L	M	L	L	L	V	V	G	F	Q	G	D	T
**bZIP11**		K	L	L	V	L	N	I	V	T	Y	V	N	L	L	L	L	L	I	L	N	N	M	C	L	L
**bZIP1**		K	M	T	I	L	I	N	C	V	L	V	N	L	K	L	V	L	I	T	L	S	D	D	A	G
**bZIP16**		K	C	L	A	L	N	L	I	L	C	L	N	L	L	F	E	S	D	E	N	G				
**bZIP68**		K	C	L	A	L	N	L	I	L	Y	L	N	L	F	A	E	D	N	E	S	Q				
**GBF1**		K	C	L	V	L	N	L	L	L	C	L	N	I	L	V	E	A	Q	A	D	G				
**GBF2**		K	T	L	V	L	N	L	L	L	S	L	N	I	L	Q	K	N	R	K	V	S				
**GBF3**		K	T	L	V	L	N	L	L	L	S	L	N	L	L	S	K	P	L	V	G	D				
**Hy5**		K	L	L	V	L	N	L	L	L	N	L	L	T	K	G										
**HyH**		K	V	L	A	L	N	L	I	L	N	L	L	T	T	N										

B)

				0			1			2			3			4			5			6			7			8			9			10			11		
	b	c	f	b	c	f	b	c	f	b	c	f	b	c	f	b	c	f	b	c	f	b	c	f	b	c	f	b	c	f	b	c	f	b	c	f	b	c	f
**bZIP10**			E	S	D	T	N	D	G	S	S	K	S	N	H	D	E	V	R	I	A	E	T	A	K	M	E	K	R	G	P	M	G	S	G	N	R	M	T
**bZIP25**			E	N	E	T	G	Q	A	S	T	N	S	D	H	D	A	V	R	I	A	E	T	T	K	M	E	K	R	G	P	L	S	N	M	P	N	T	A
**bZIP63**			A	S	E	T	S	Q	V	S	K	K	T	D	Q	N	D	V	R	V	A	E	T	A	K	M	E	K	R	G	P	M	H	Q	I	T	L	P	T
**bZIP9**			E	V	D	T	D	S	G	S	T	K	I	D	Q	R	S	T	R	V	S	E	T	V	K	L	D	A	R	L	S	L	L	T	H	P	H	S	S
**bZIP44**			K	D	D	A	T	H	K	A	Q	A	A	V	Q	V	T	A	D	I	A	L	E	H	Q	S	E	D	F	S	S	G	M	G	Q	F	G	L	G
**bZIP53**			K	G	D	N	T	L	N	A	K	E	D	E	K	I	E	S	N	V	A	S	E	D	R	S	S	E	M	E	G	Q	D	E	I	S	N	P	M
**bZIP2**			K	D	D	A	N	Q	N	R	Q	N	T	V	Q	M	K	A	S	V	A	E	E	T	Q	S	E	D	L	S	A	G	V	I	D	G	D	R	G
**bZIP11**			K	D	D	A	N	H	K	T	E	T	S	I	Q	L	T	A	S	V	A	D	E	H	Q	S	D	E	F	S	N	N	N	G	M	N	V	G	C
**bZIP1**			K	E	D	H	S	S	R	K	E	E	R	A	Q	D	S	T	A	G	S	I	W	S	S	D	N	A	T	L	T	Q	G	C	V	Q	N	A	A
**bZIP16**			A	D	E	Q	A	V	E	T	N	A	N	N	S	E	E	T	T	S	D	S	L	P	G	I	D	H	Q	D	Q	T	A						
**bZIP68**			A	D	E	Q	A	V	G	S	S	A	N	N	S	E	E	A	S	S	N	S	S	S	G	G	D	E	Q	Q	T	R	V						
**GBF1**			A	E	Q	Q	Q	S	N	Q	S	D	Q	Q	S	D	K	S	N	S	D	Q	R	G	A	V	L	N	A	S	G	E	N						
**GBF2**			A	E	Q	V	S	A	A	M	S	S	G	G	N	E	K	L	E	A	D	K	A	T	T	E	I	V	D	N	S	G	T						
**GBF3**			A	E	E	R	A	A	A	M	A	S	N	N	E	D	K	G	A	T	D	K	C	P	R	V	N	S	R	N	A	G	A						
**Hy5**			A	S	E	N	E	D	N	S	E	E	S	S	N	Q	M	H	K	N	G	R	G																
**HyH**			V	S	D	S	E	E	N	D	Q	E	S	S	N	T	M	K	I	N	P	D	D																

C)

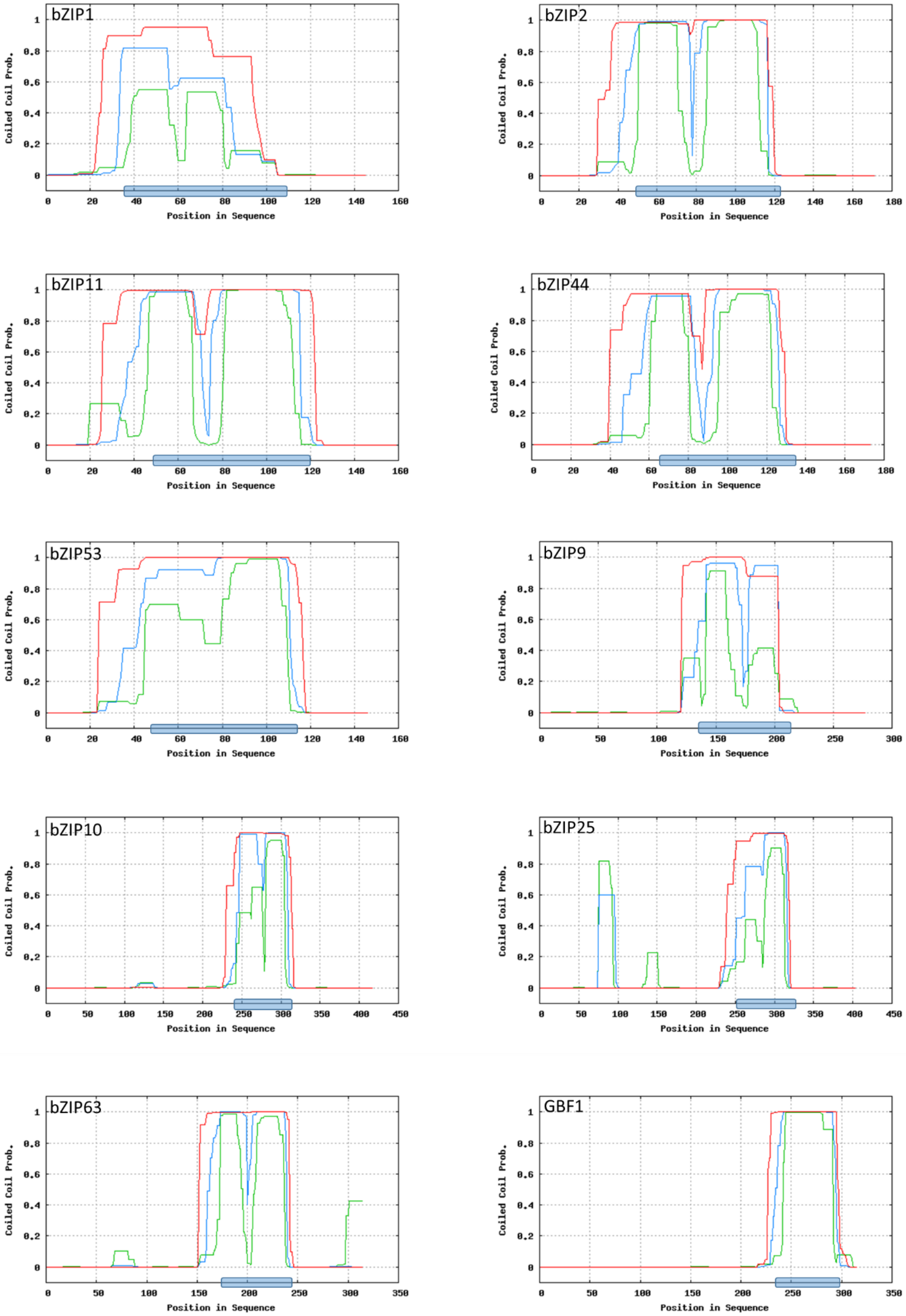
bZIP1
bZIP2
bZIP11
bZIP44
bZIP53
bZIP9
bZIP10
bZIP25
bZIP63
GBF1
Coiled Coil Prob.
Position in Sequence

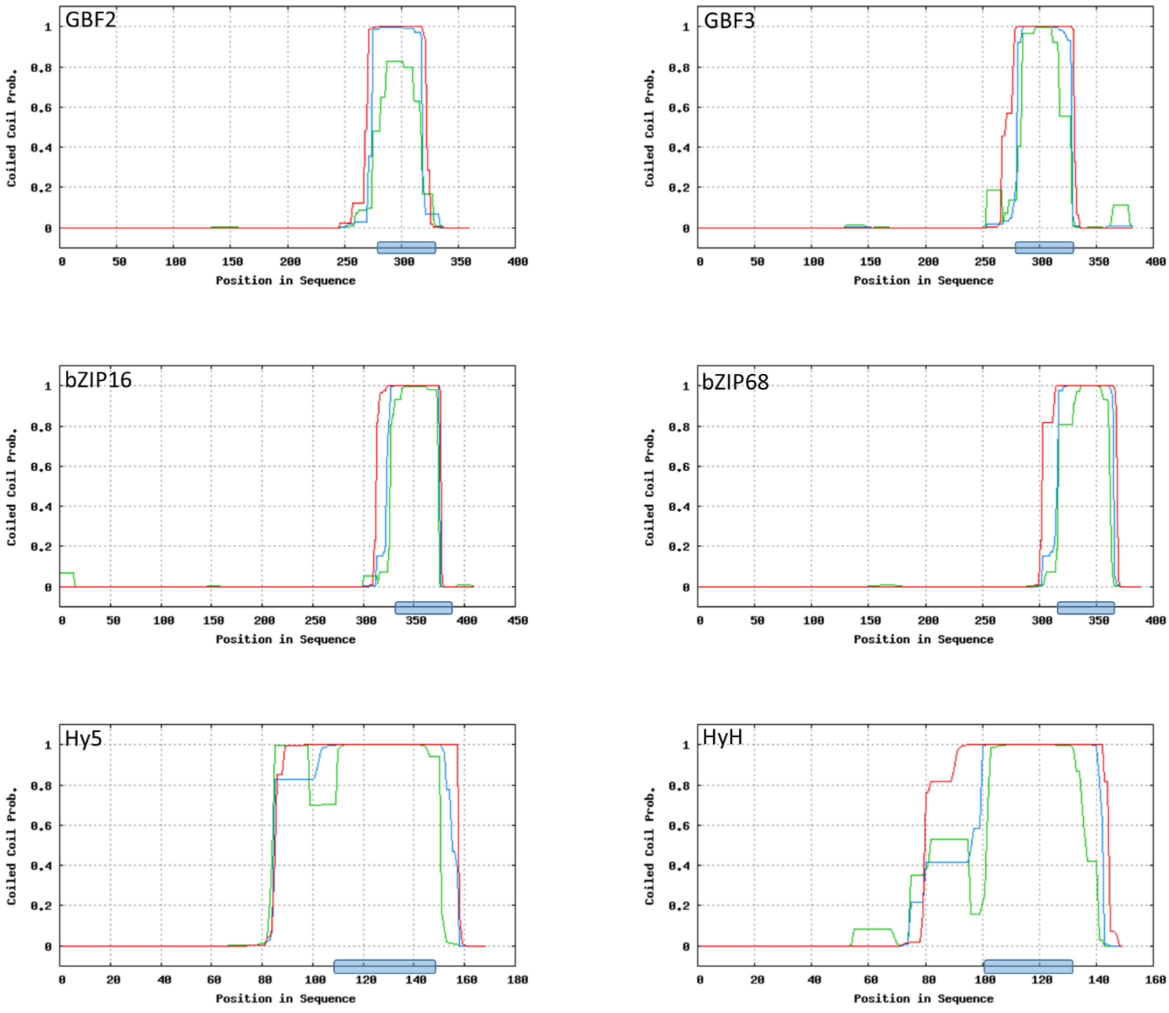
GBF2
GBF3
bZIP16
bZIP68
Hy5
HyH
Coiled Coil Prob.
Position in Sequence

D)

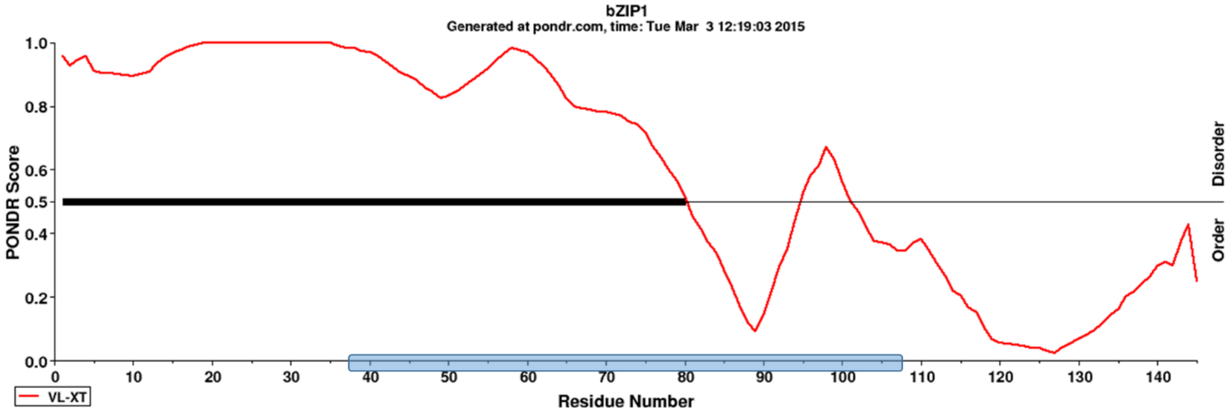
bZIP1
Generated at pondr.com, time: Tue Mar 3 12:19:03 2015
PONDR Score
Residue Number
Disorder
Order
VL-XT

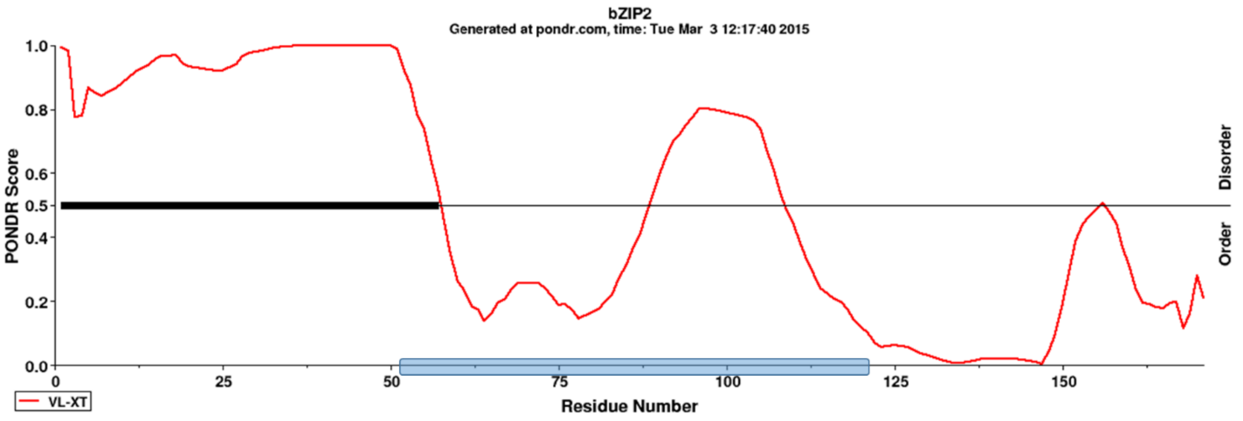
bZIP2
Generated at pondr.com, time: Tue Mar 3 12:17:40 2015
PONDR Score
1.0
0.8
0.6
0.5
0.4
0.2
0.0
0
25
50
75
100
125
150
Residue Number
VL-XT
Disorder
Order

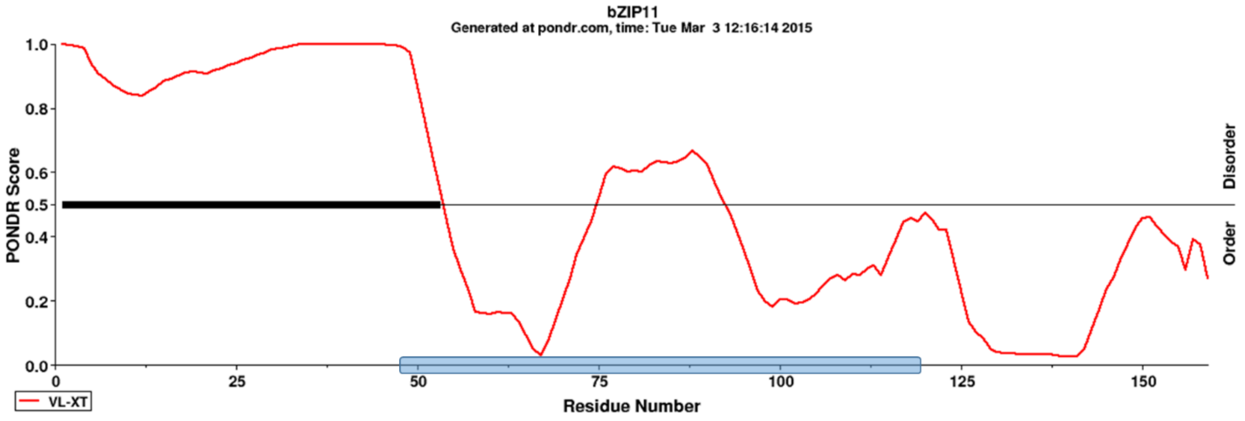
bZIP11
Generated at pondr.com, time: Tue Mar 3 12:16:14 2015
PONDR Score
1.0
0.8
0.6
0.5
0.4
0.2
0.0
0
25
50
75
100
125
150
Residue Number
VL-XT
Disorder
Order

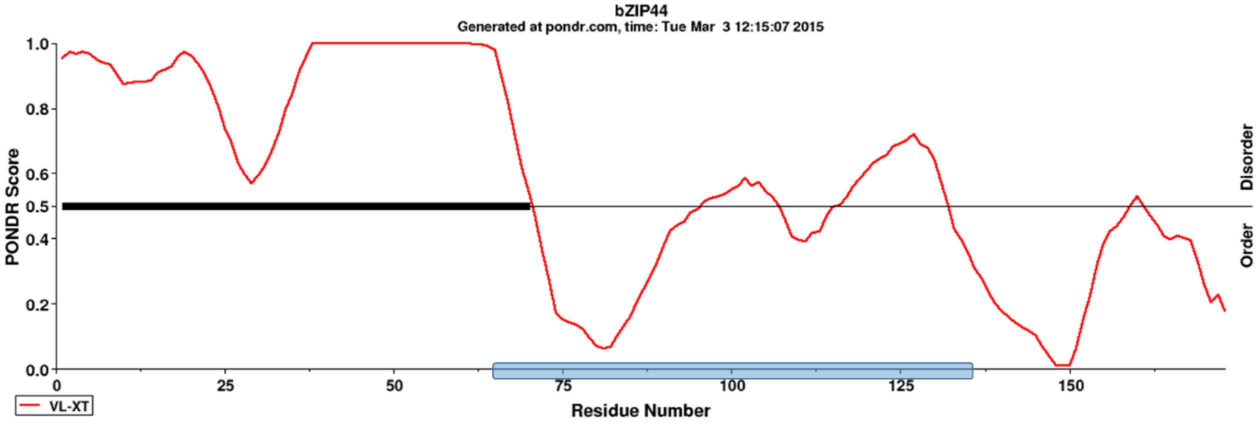
bZIP44
Generated at pondr.com, time: Tue Mar 3 12:15:07 2015
PONDR Score
1.0
0.8
0.6
0.5
0.4
0.2
0.0
0
25
50
75
100
125
150
Residue Number
VL-XT
Disorder
Order

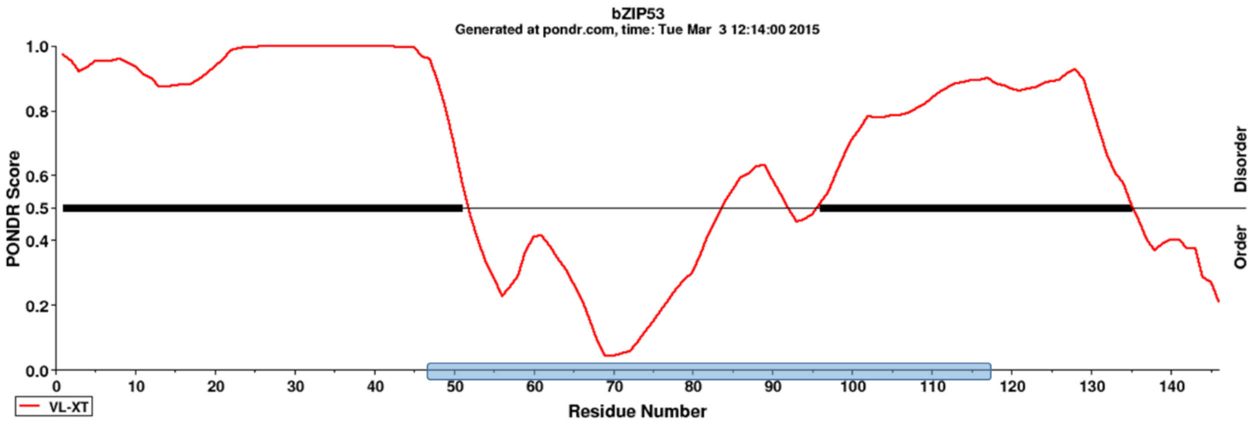
bZIP53
Generated at pondr.com, time: Tue Mar 3 12:14:00 2015
PONDR Score
1.0
0.8
0.6
0.5
0.4
0.2
0.0
0
10
20
30
40
50
60
70
80
90
100
110
120
130
140
Residue Number
VL-XT
Disorder
Order

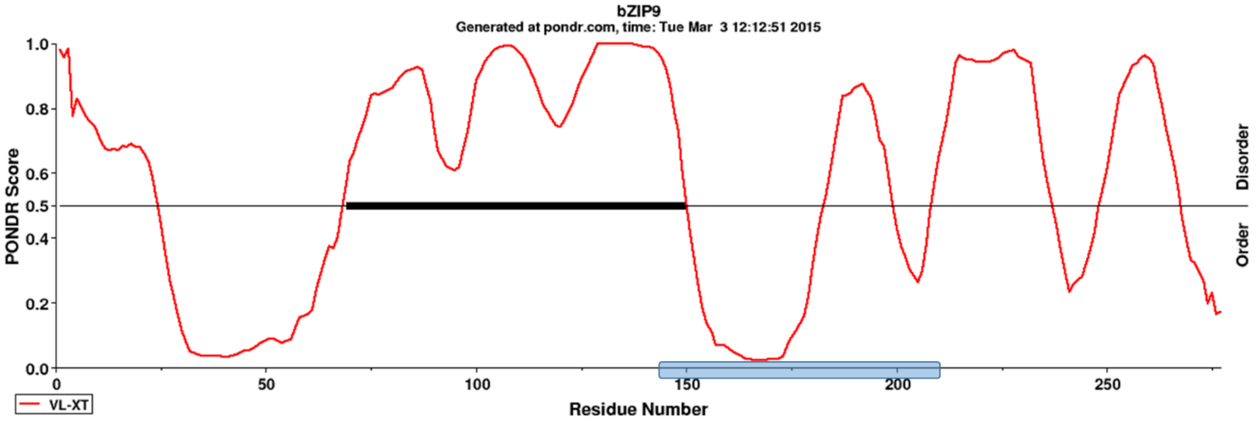
bZIP9
Generated at pondr.com, time: Tue Mar 3 12:12:51 2015
PONDR Score
Residue Number
Disorder
Order
VL-XT

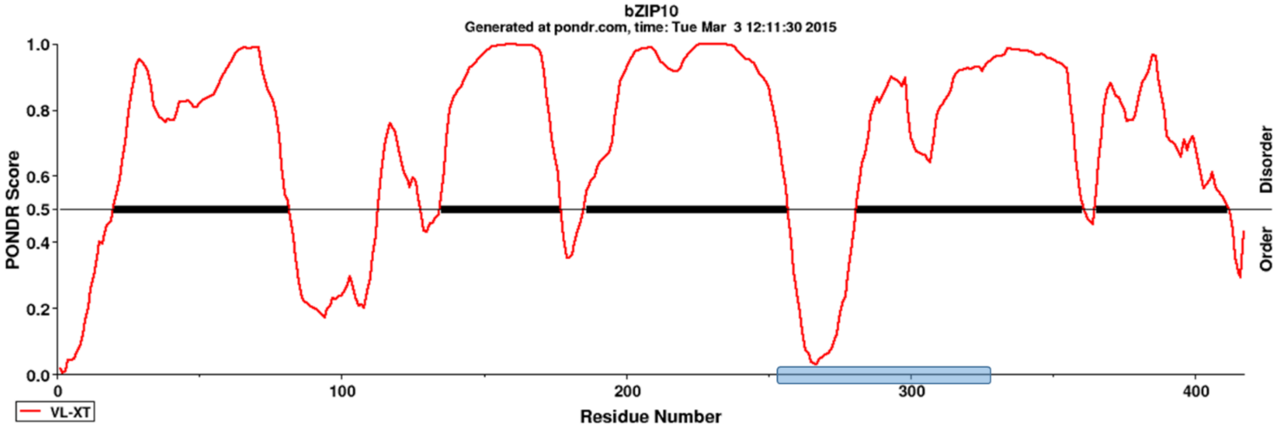
bZIP10
Generated at pondr.com, time: Tue Mar 3 12:11:30 2015
PONDR Score
Residue Number
Disorder
Order
VL-XT

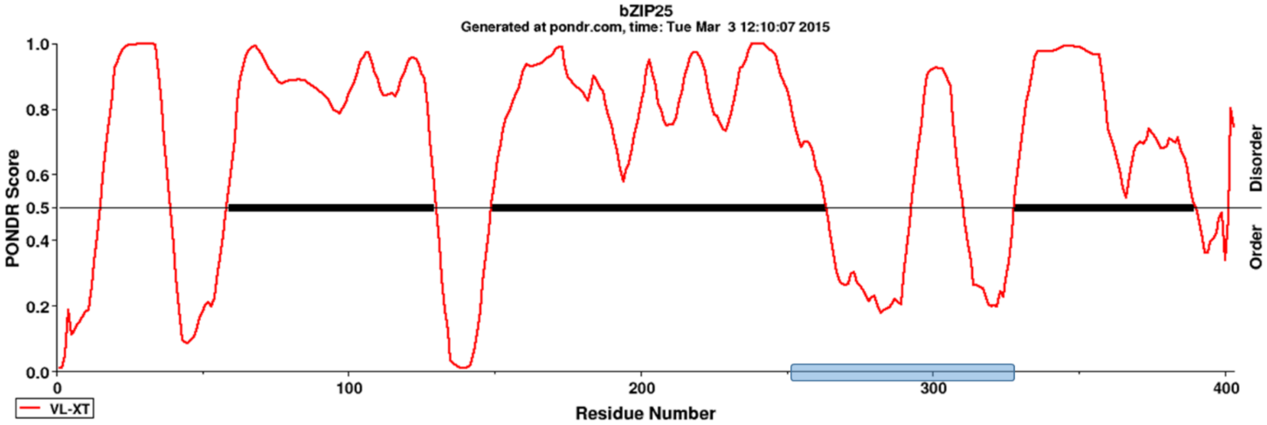
bZIP25
Generated at pondr.com, time: Tue Mar 3 12:10:07 2015
PONDR Score
Residue Number
Disorder
Order
VL-XT

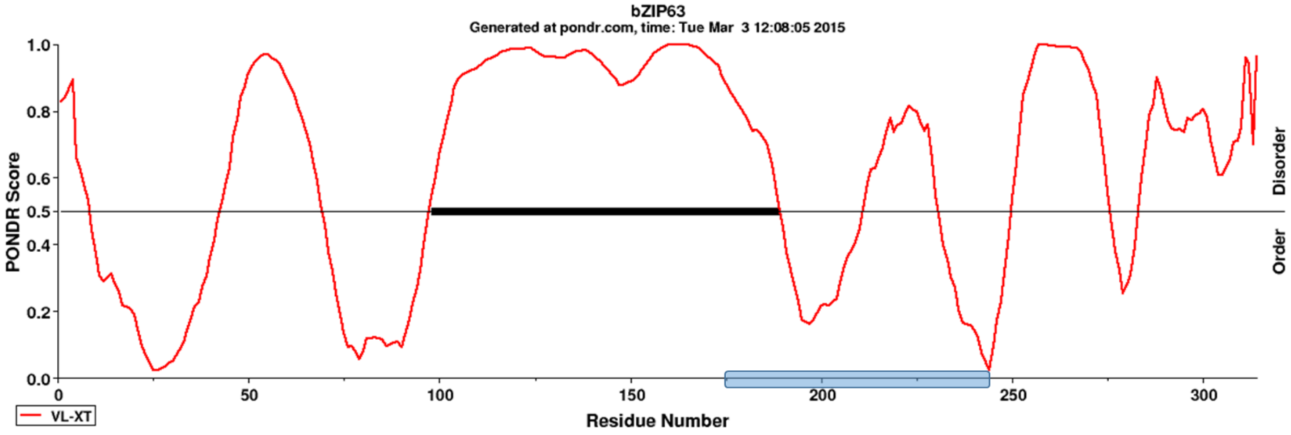
bZIP63
Generated at pondr.com, time: Tue Mar 3 12:08:05 2015
PONDR Score
Residue Number
Disorder
Order
VL-XT

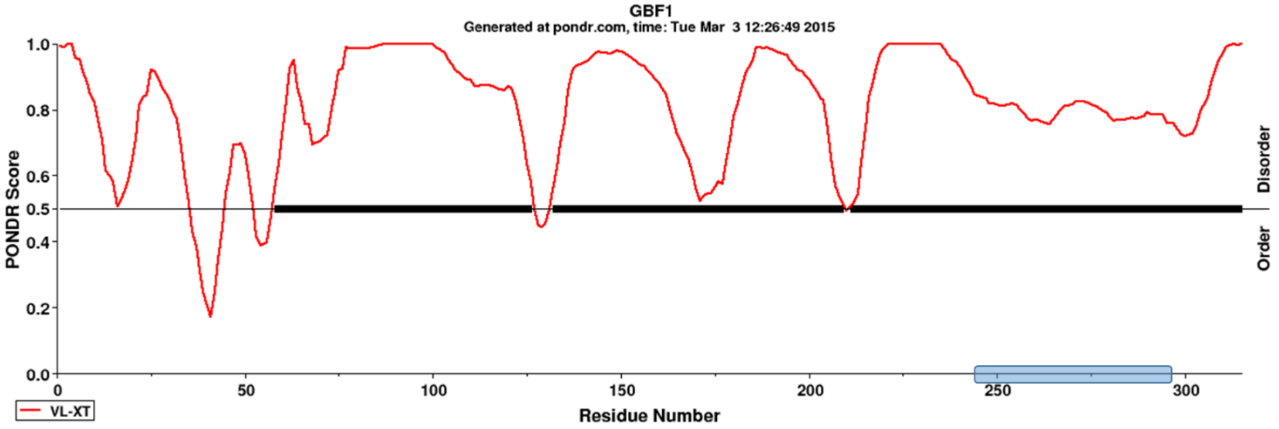
GBF1
Generated at pondr.com, time: Tue Mar 3 12:26:49 2015
PONDR Score
Residue Number
Disorder
Order
VL-XT

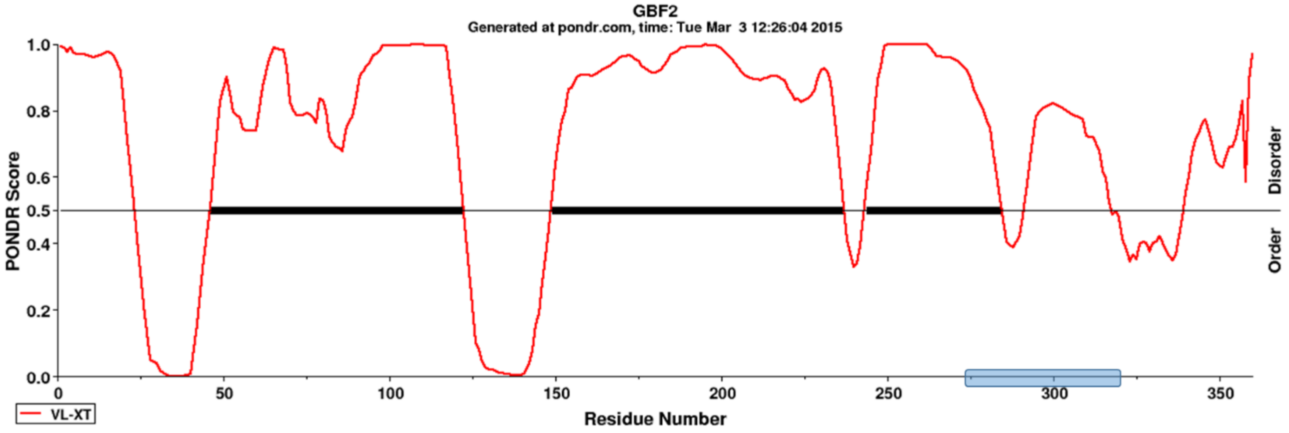
GBF2
Generated at pondr.com, time: Tue Mar 3 12:26:04 2015
PONDR Score
Residue Number
Disorder
Order
VL-XT

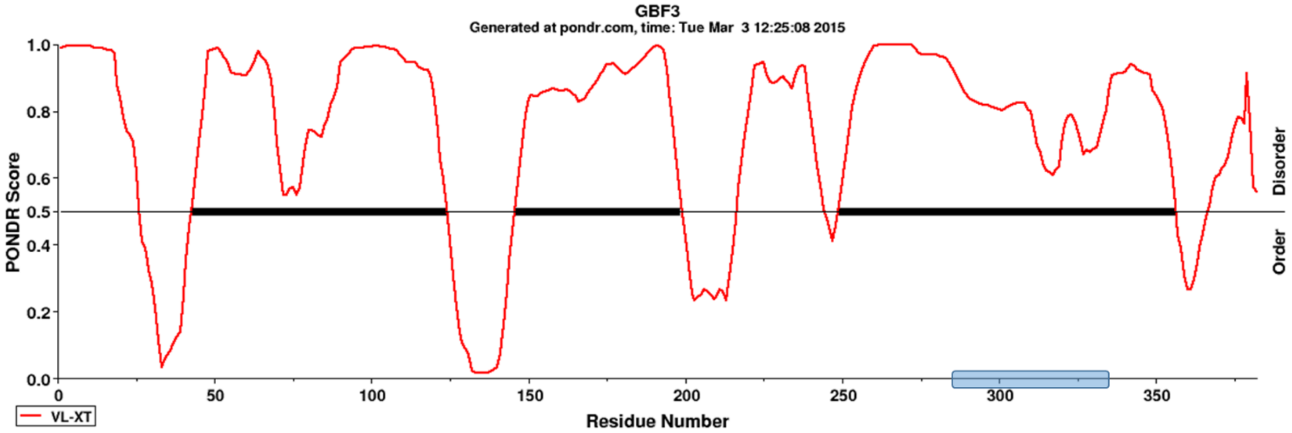
GBF3
Generated at pondr.com, time: Tue Mar 3 12:25:08 2015
PONDR Score
Residue Number
Disorder
Order
VL-XT

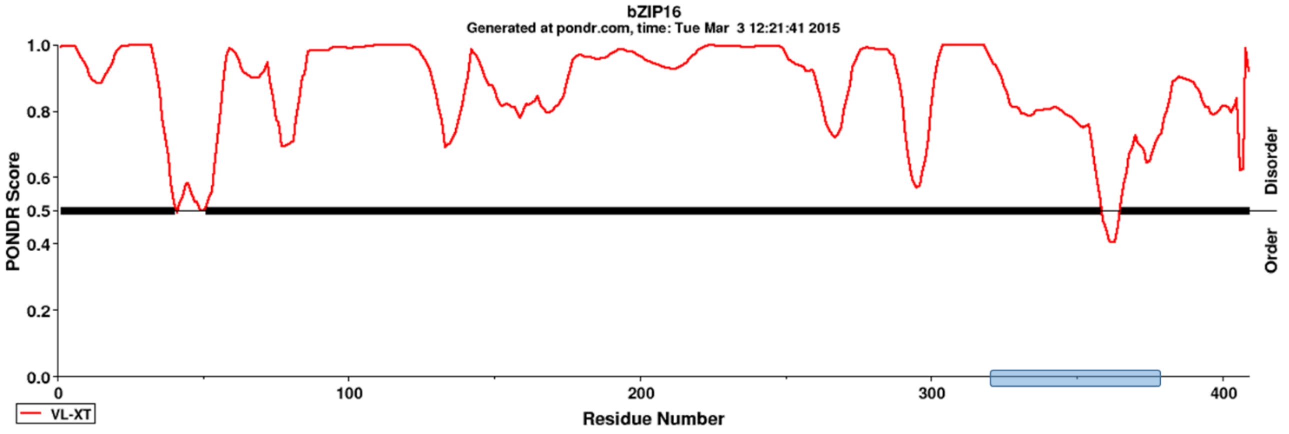
bZIP16
Generated at pondr.com, time: Tue Mar 3 12:21:41 2015
PONDR Score
Residue Number
Disorder
Order
VL-XT

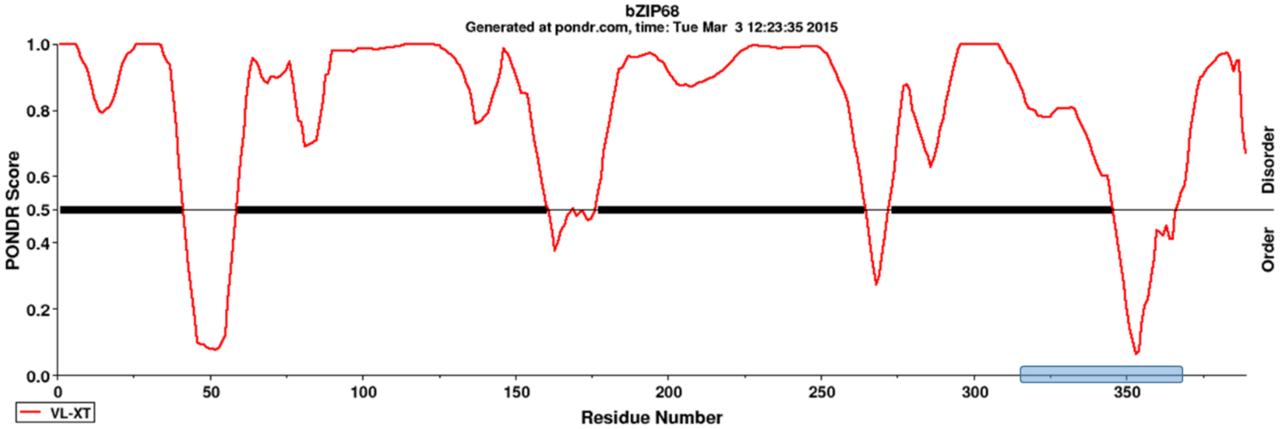
bZIP68
Generated at pondr.com, time: Tue Mar 3 12:23:35 2015
PONDR Score
Residue Number
Disorder
Order
VL-XT

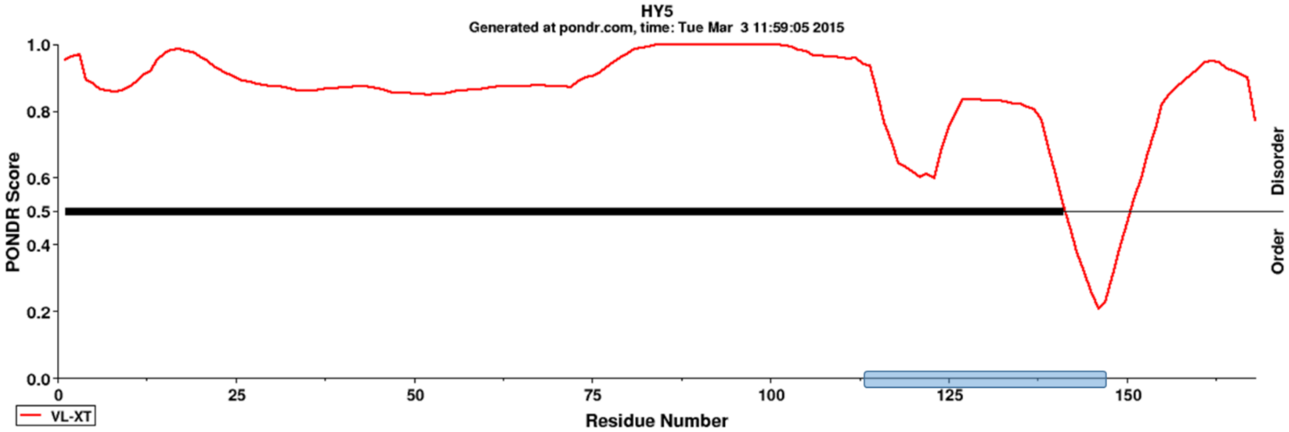
HY5
Generated at pondr.com, time: Tue Mar 3 11:59:05 2015
PONDR Score
Residue Number
Disorder
Order
VL-XT

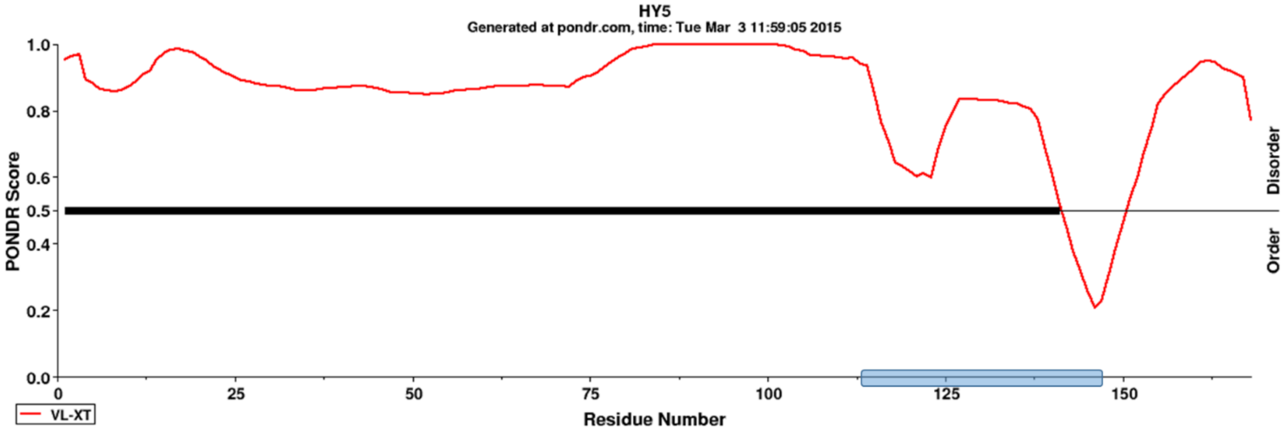
HY5
Generated at pondr.com, time: Tue Mar 3 11:59:05 2015
PONDR Score
Residue Number
Disorder
Order
VL-XT
