# Supplementary material for: The Elucidation of the Interactome of 16 Arabidopsis bZIP Factors Reveals Three Independent Functional Networks
Source: PLoS One. 2015 Oct 9;10(10):e0139884. doi: 10.1371/journal.pone.0139884 (PMC4599898; doi:10.1371/journal.pone.0139884)
Supplement: S4 Fig — In all cases the expression levels are represented in Log2 scale. A) Scatterplot with the expression across different tissues according to the qPCR experiments. B) Levels across the different developmental stages. C) Expression of the bZIPs across tissues as in A) but here in boxplot format in order to reflect the high variability in the measurements. (PDF) [file pone.0139884.s004.pdf]

A)

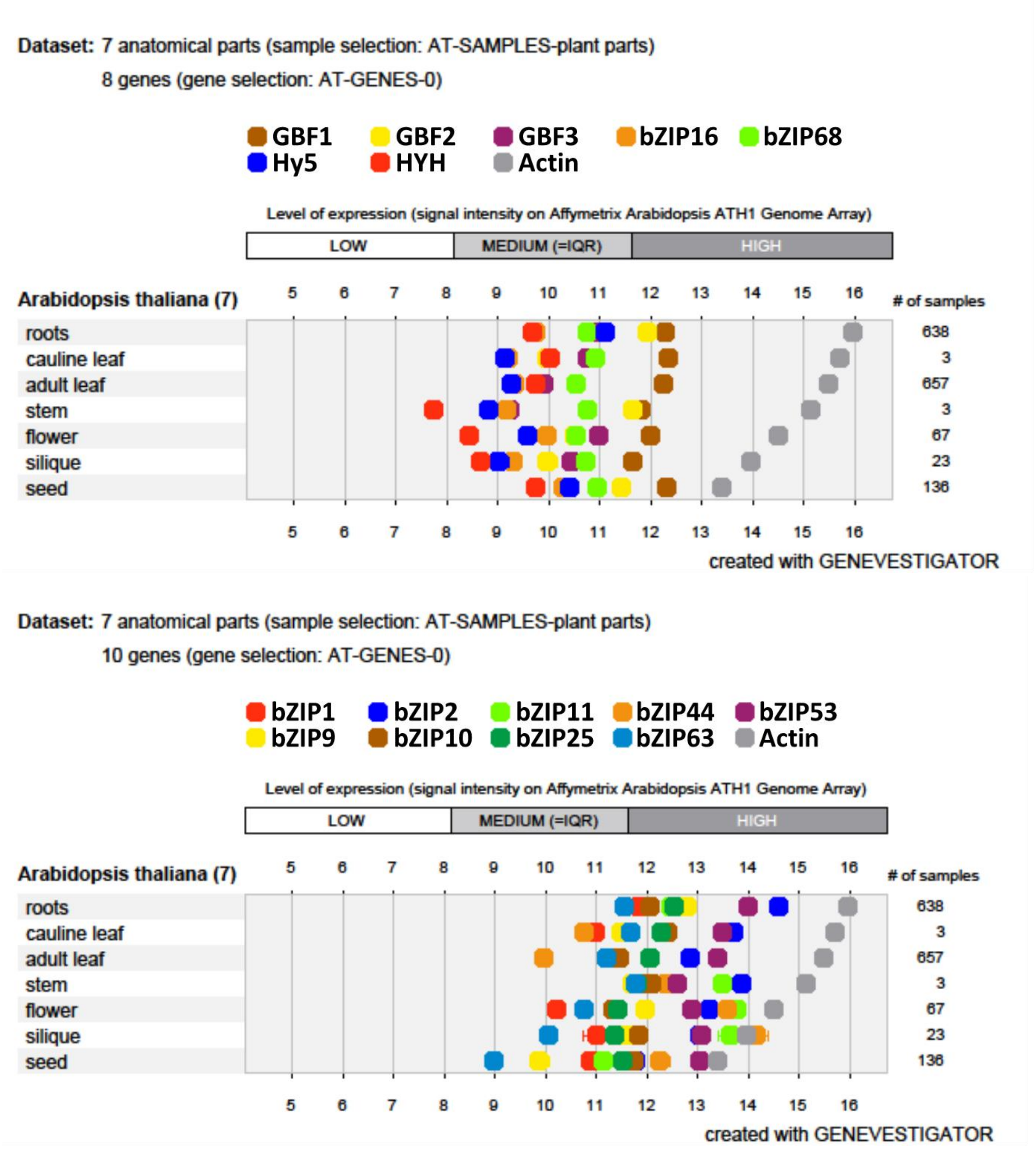
Dataset: 7 anatomical parts (sample selection: AT-SAMPLES-plant parts)
8 genes (gene selection: AT-GENES-0)
GBF1
GBF2
GBF3
bZIP16
bZIP68
Hy5
HYH
Actin
Level of expression (signal intensity on Affymetrix Arabidopsis ATH1 Genome Array)
LOW
MEDIUM (=IQR)
HIGH
Arabidopsis thaliana (7)
5 6 7 8 9 10 11 12 13 14 15 16
# of samples
roots 638
cauline leaf 3
adult leaf 657
stem 3
flower 67
silique 23
seed 136
created with GENEVESTIGATOR
Dataset: 7 anatomical parts (sample selection: AT-SAMPLES-plant parts)
10 genes (gene selection: AT-GENES-0)
bZIP1
bZIP2
bZIP11
bZIP44
bZIP53
bZIP9
bZIP10
bZIP25
bZIP63
Actin
Level of expression (signal intensity on Affymetrix Arabidopsis ATH1 Genome Array)
LOW
MEDIUM (=IQR)
HIGH
Arabidopsis thaliana (7)
5 6 7 8 9 10 11 12 13 14 15 16
# of samples
roots 638
cauline leaf 3
adult leaf 657
stem 3
flower 67
silique 23
seed 136
created with GENEVESTIGATOR

B)

Dataset: 10 developmental stages (sample selection: AT-SAMPLES-plant parts)
8 genes (gene selection: AT-GENES-0)

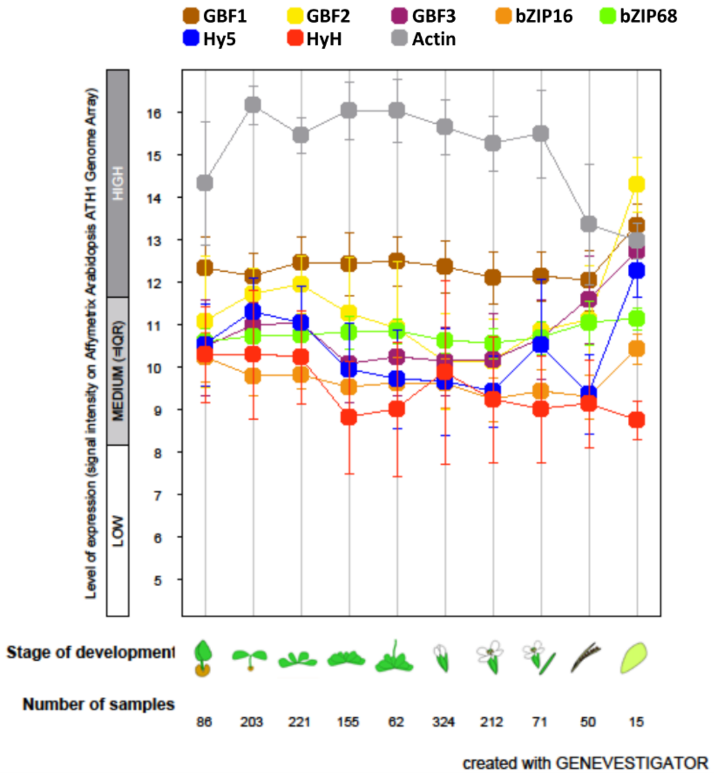

Dataset: 10 developmental stages (sample selection: AT-SAMPLES-plant parts)
10 genes (gene selection: AT-GENES-0)

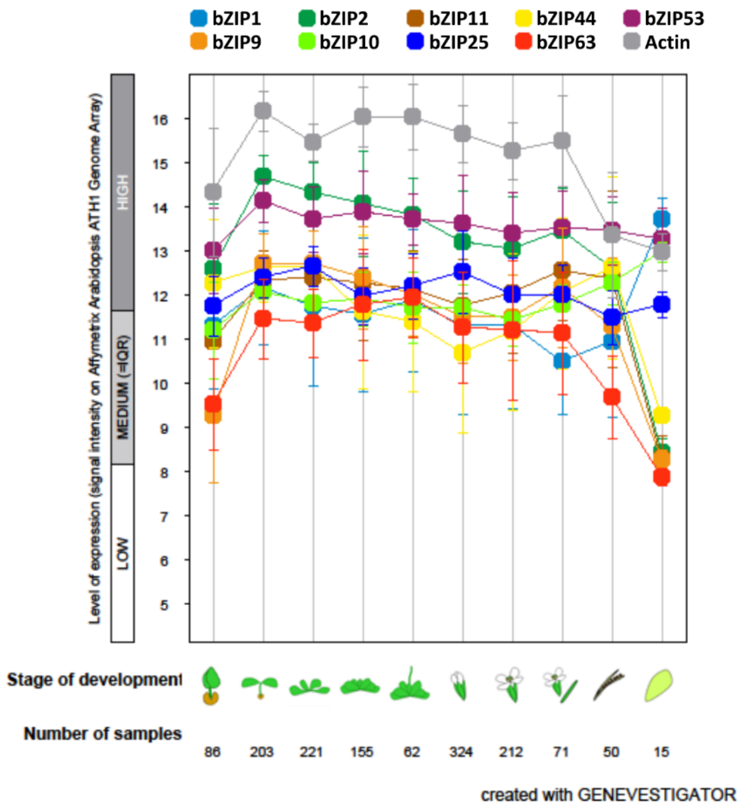

C)

Actin

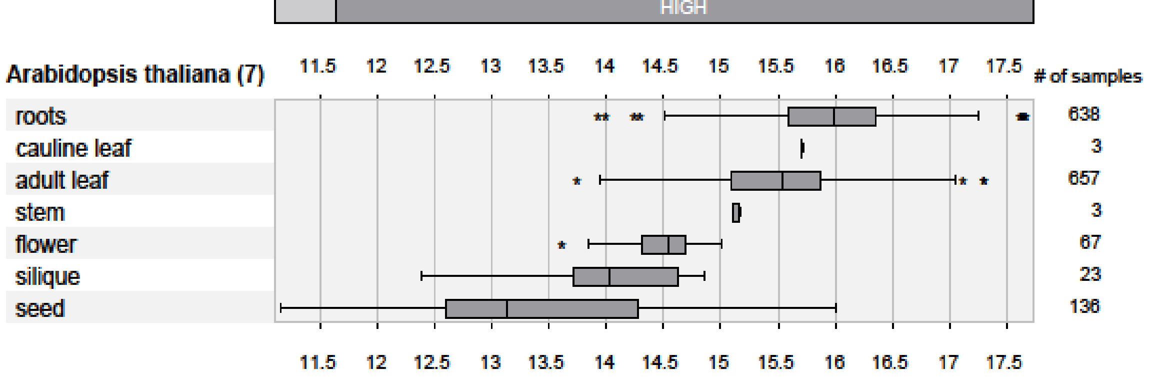

bZIP1

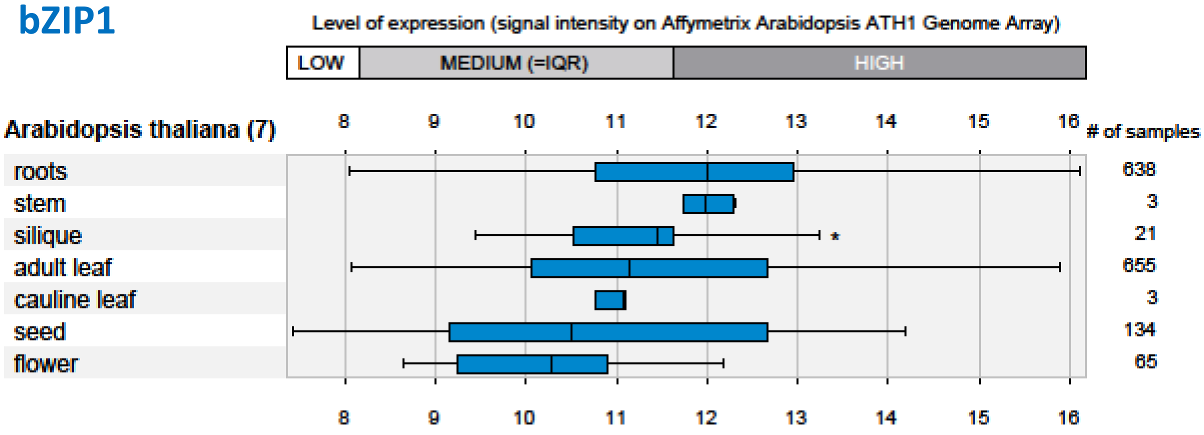

bZIP2

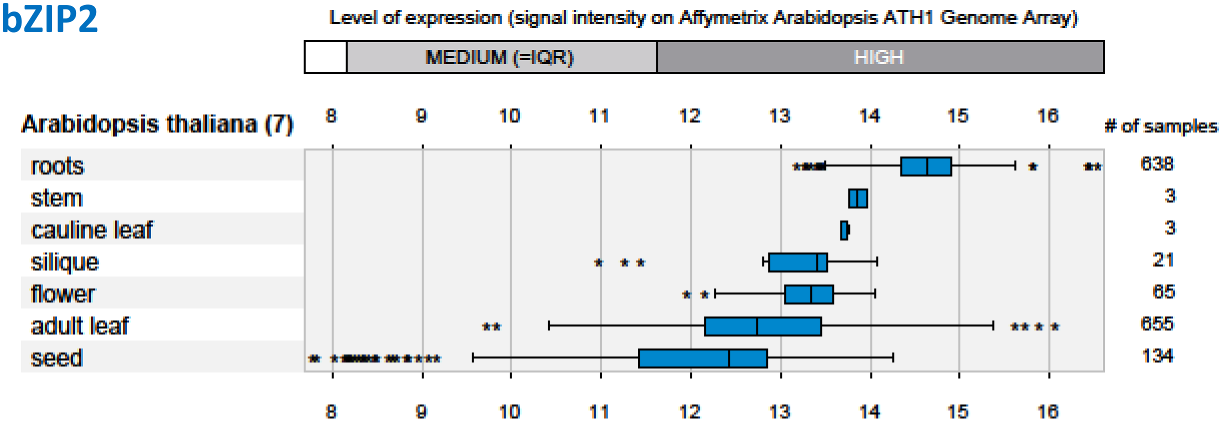

bZIP11

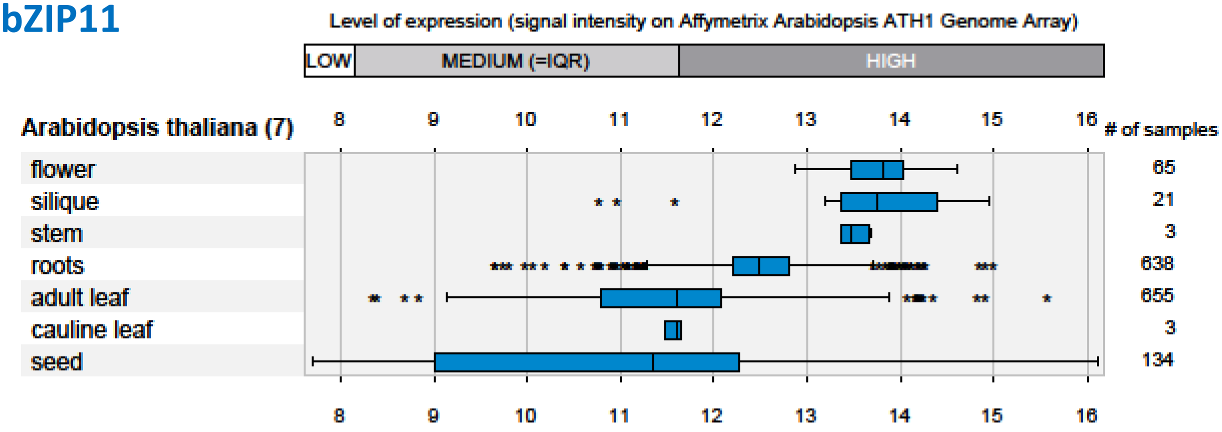

bZIP44

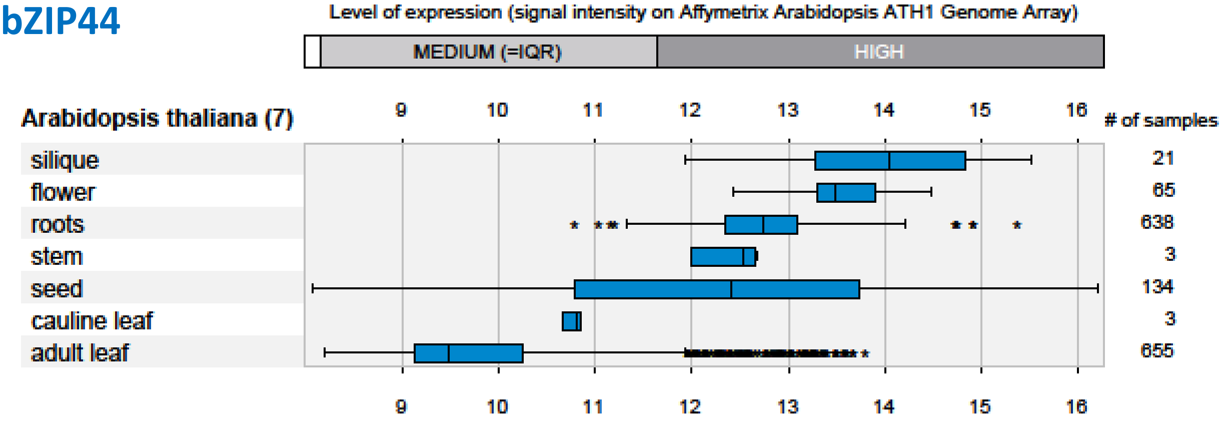

bZIP53

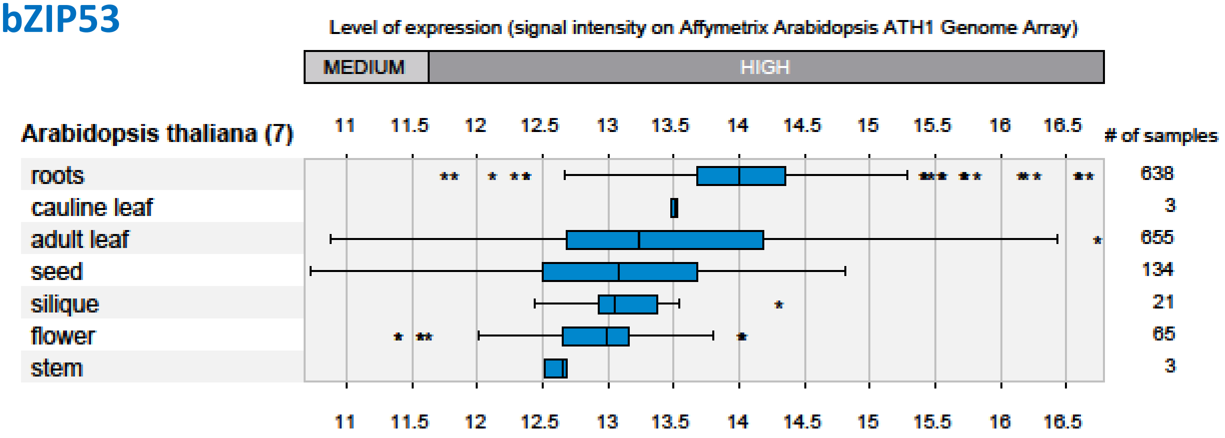

bZIP9

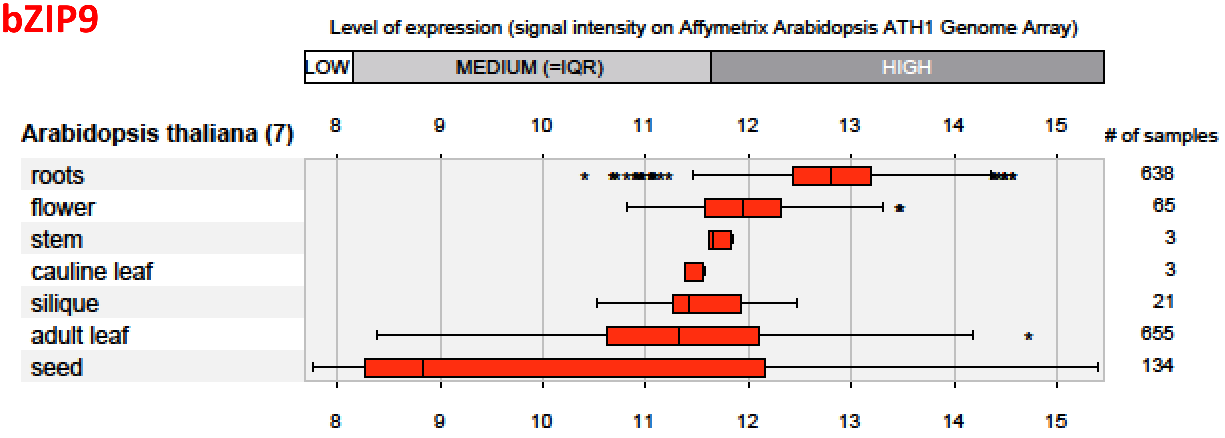

bZIP10

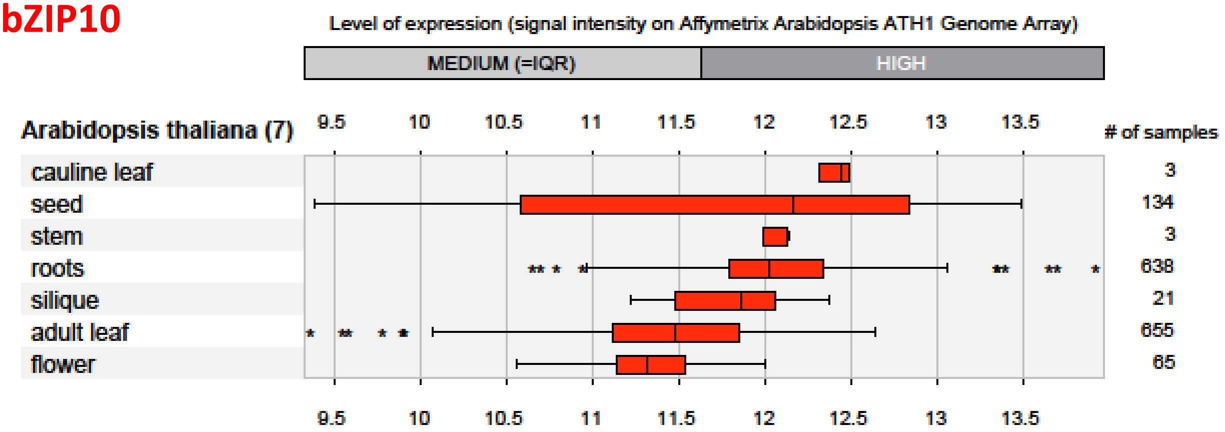

bZIP25

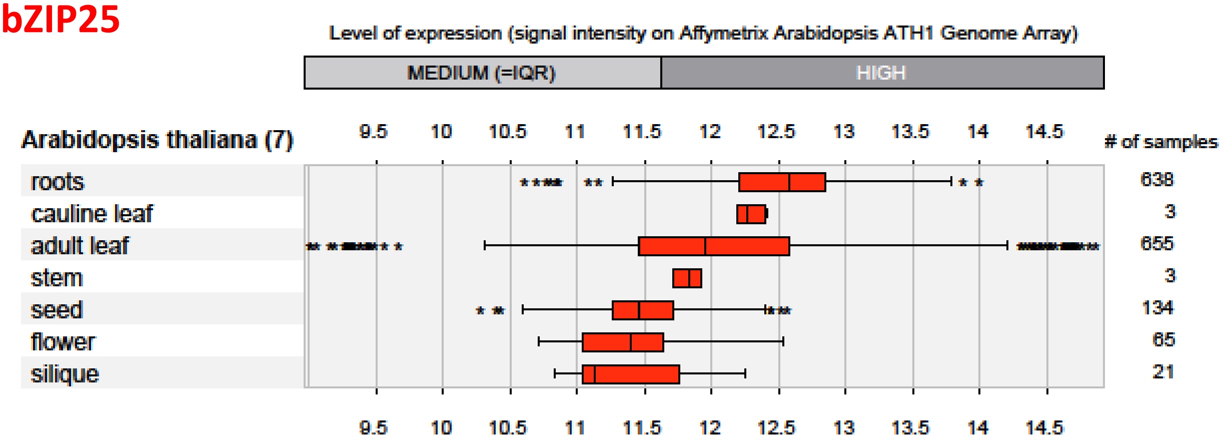

bZIP63

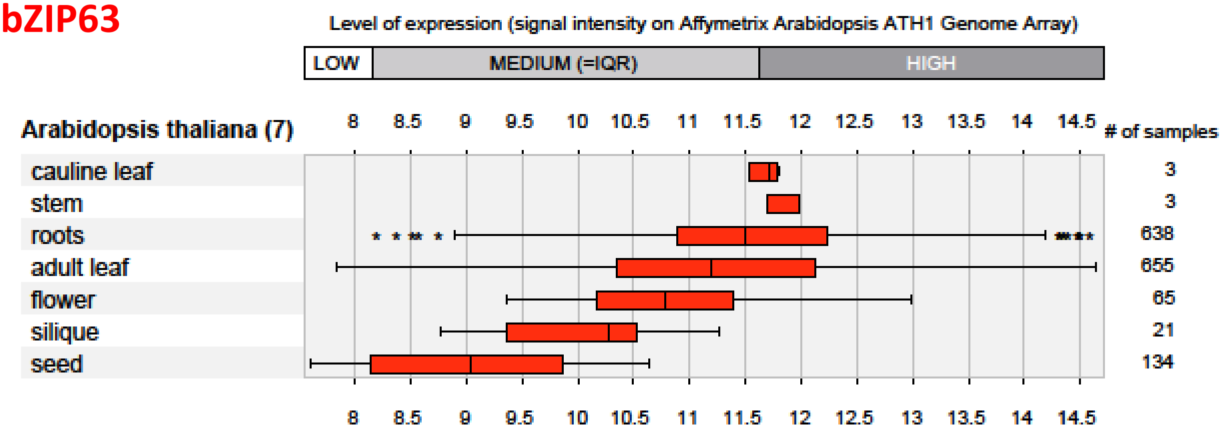

GBF1

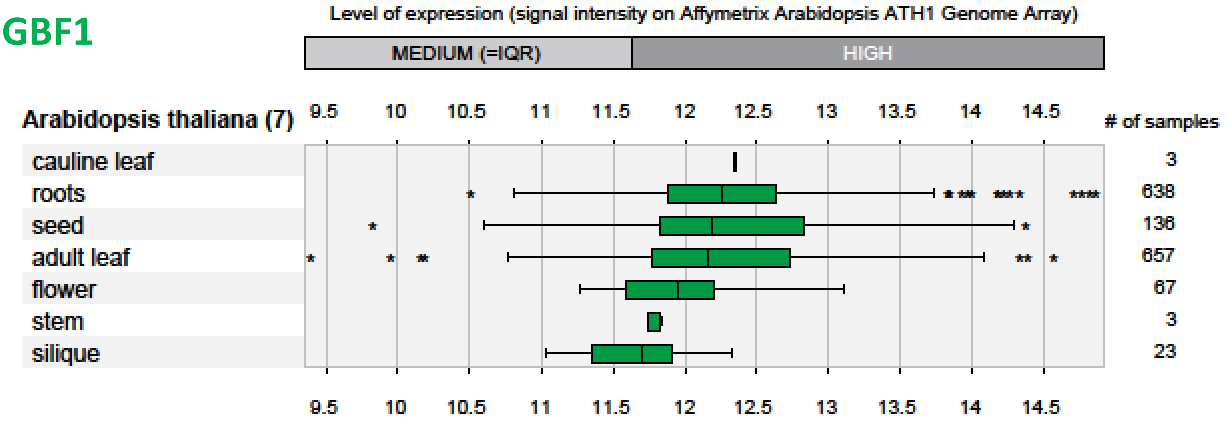

GBF2

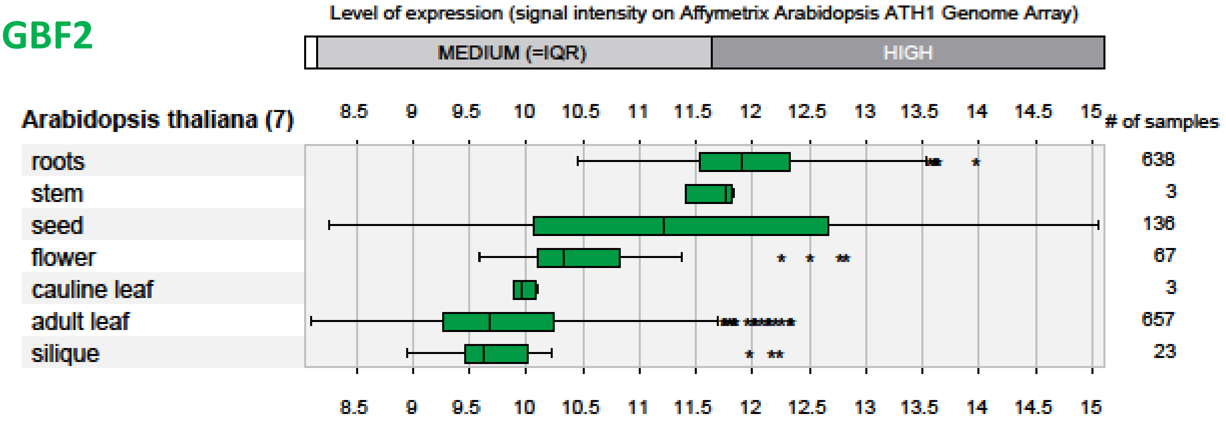

GBF3

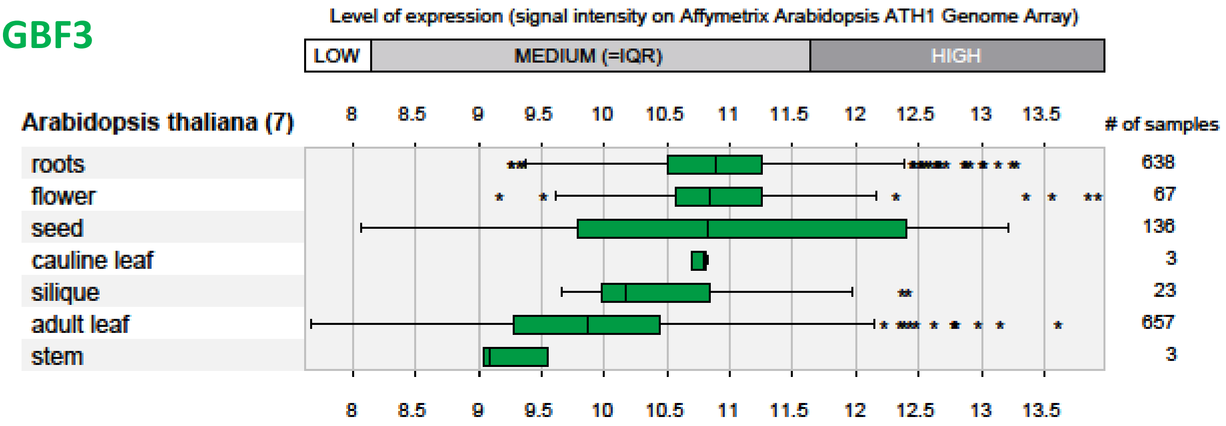

bZIP16

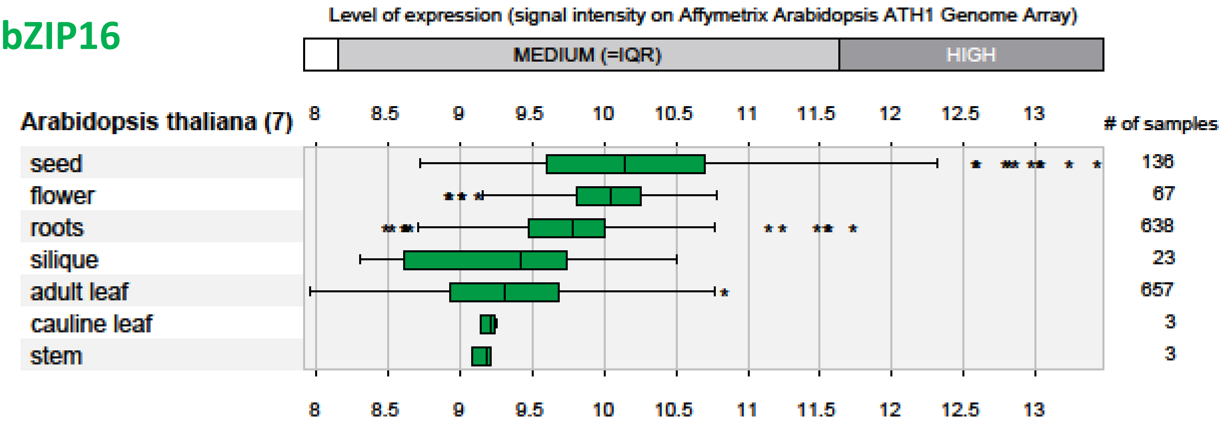

bZIP68

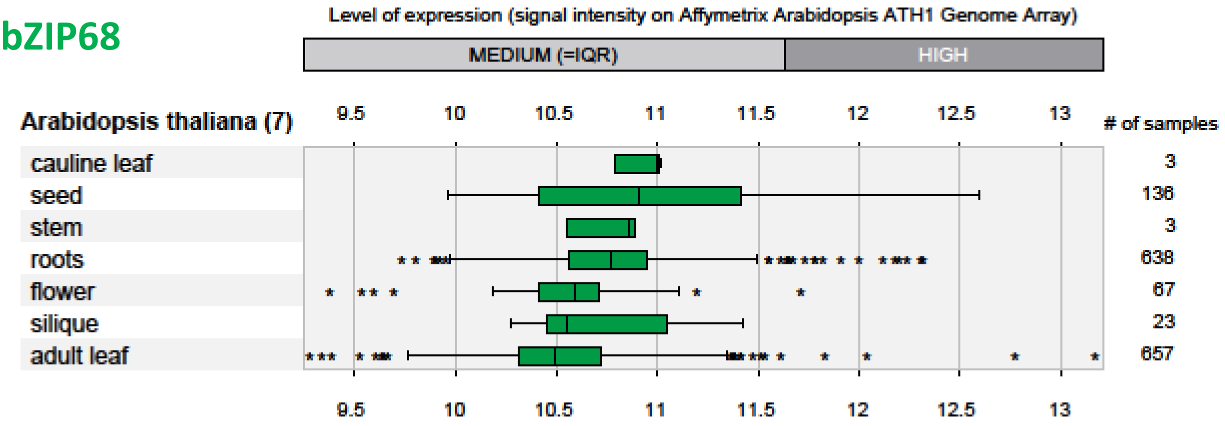

Hy5

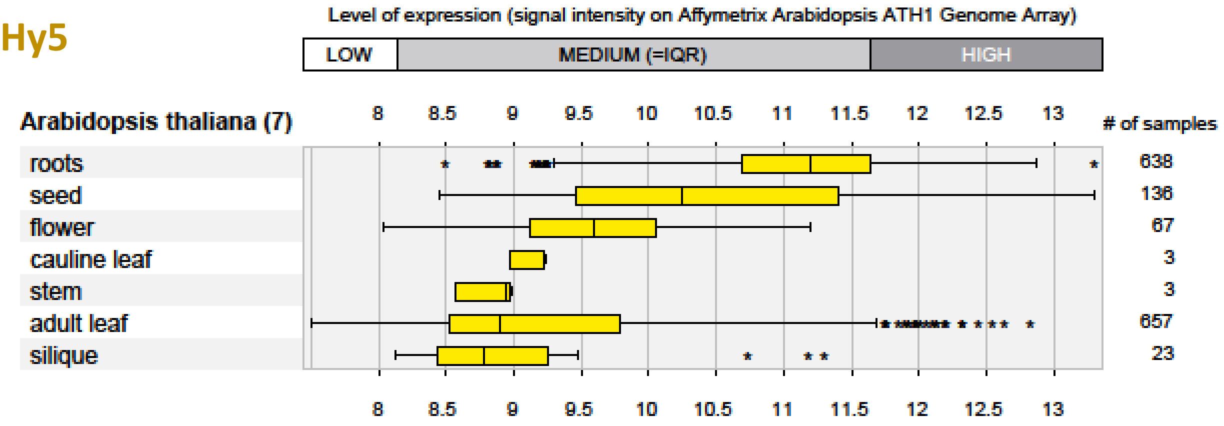

HyH

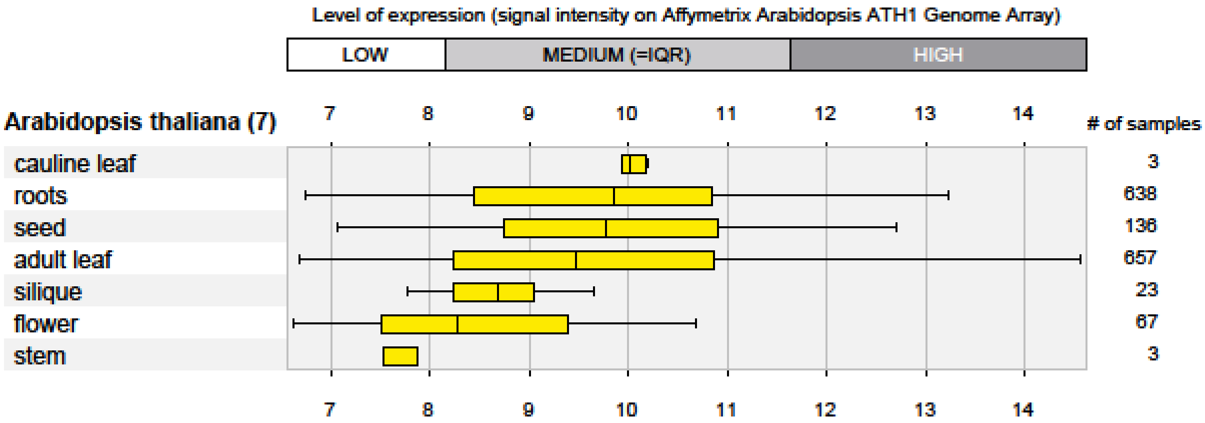
Level of expression (signal intensity on Affymetrix Arabidopsis ATH1 Genome Array)
LOW
MEDIUM (=IQR)
HIGH
Arabidopsis thaliana (7)
7
8
9
10
11
12
13
14
# of samples
cauline leaf
roots
seed
adult leaf
silique
flower
stem
3
638
136
657
23
67
3
