# Supplementary material for: The Elucidation of the Interactome of 16 Arabidopsis bZIP Factors Reveals Three Independent Functional Networks
Source: PLoS One. 2015 Oct 9;10(10):e0139884. doi: 10.1371/journal.pone.0139884 (PMC4599898; doi:10.1371/journal.pone.0139884)
Supplement: S3 Table — The groups were tested using the Estimate statement of Proc Glimmix. The resulting p-values were adjusted using the Scheffé method [82]. (PDF) [file pone.0139884.s009.pdf]

Estimates
Adjustment for Multiplicity: Scheffe

Comparisons	*Estimate	Standard Error	DF	t Value	Pr > |t|	Adj P
**HH-GG**	0.5692	0.1106	873	5.15	<.0001	<.0001
**HH-S1C**	1.8954	0.1287	873	14.73	<.0001	<.0001
**HH-S1S1**	2.2750	0.1340	873	16.98	<.0001	<.0001
**HH-CC**	2.6189	0.1506	873	17.39	<.0001	<.0001
**HH-Rest**	3.2624	0.1086	873	30.04	<.0001	<.0001
**GG-S1C**	1.3261	0.08236	873	16.10	<.0001	<.0001
**GG-S1S1**	1.7058	0.09067	873	18.81	<.0001	<.0001
**GG-CC**	2.0497	0.1131	873	18.13	<.0001	<.0001
**GG-Rest**	2.6932	0.05274	873	51.07	<.0001	<.0001
**S1C-S1S1**	0.2850	0.07586	873	3.76	0.0002	0.0002
**S1C-CC**	0.7236	0.1005	873	7.20	<.0001	<.0001
**S1C-Rest**	1.3670	0.07308	873	18.70	<.0001	<.0001
**S1S1-CC**	0.3439	0.1065	873	3.23	0.0013	0.0025
**S1S1-Rest**	0.9874	0.08224	873	12.01	<.0001	<.0001
**CC-Rest**	0.6434	0.1063	873	6.05	<.0001	<.0001

* estimates are on logit scale
